# The Impact of rAdo and ^OXO^dGuo on the Electronic Properties of Clustered DNA Lesions: A Theoretical Approach

**DOI:** 10.3390/molecules31203673

**Published:** 2026-10-14

**Authors:** Boleslaw T. Karwowski

**Affiliations:** Nucleic Acids Damage Laboratory of Food Science Department, Faculty of Pharmacy, Medical University of Lodz, ul. Muszyńskiego 1, 90-151 Lodz, Poland; boleslaw.karwowski@umed.lodz.pl

**Keywords:** ribonucleosides, 7,8-dihydro-8-oxo-2′-deoxyguanosine, clustered DNA lesions, vertical and adiabatic ionisation potential and electron affinity, Fukui functions, density functional theory

## Abstract

Ribonucleotides and 7,8-dihydro-8-oxo-2′-deoxyguanosine (^O^G) are frequently incorporated into genomic DNA as non-canonical moieties; their local co-occurrence can lead to the formation of clustered DNA lesions (CDLs) which may not be susceptible to repair. Their estimated co-occurrence within two helical turns may reach ~650 incidents per diploid genome and can increase in pathological states. The results presented in this article show the influence of riboadenosine (rAdo) on the structural and electronic properties of double-stranded (ds) DNA containing ^O^G. For this purpose, four ds-pentamers representing different mutual rAdo and ^O^G topology, i.e., d**[**A_1_G_2_rA_3_^O^G_4_A_5_]*d**[**T_5_C_4_T_3_C_2_T_1_**]** (GrA^O^G-DNA), d**[**A_1_^O^G_2_rA_3_G_4_A_5_]*d**[**T_5_C_4_T_3_C_2_T_1_**]** (^O^GrAG-DNA), d**[**A_1_G_2_rA_3_G_4_A_5_]*d**[**T_5_C_4_^O^G_3_C_2_T_1_**]** (rA^O^G-DNA) and d**[**A_1_G_2_rA_3_^O^G_4_A_5_]*d**[**T_5_C_4_T_3_A_2_T_1_**]** (^O^GA-DNA), have been selected and theoretically investigated at a M06-2X/6-31++G**//M06-2X/D95**:M06-2X/sto-3G level of theory in aqueous solution. The study examines their spatial geometry, stacking interactions, charge and spin distributions, Fukui functions, global reactivity descriptors, and vertical/adiabatic ionisation potentials and electron affinities. The above parameters of ds-oligonucleotides were found to be strongly dependent on topology. rA^O^G-DNA was the most resistant to charge-induced structural rearrangement, whereas ^O^GA-DNA was the most responsive and exhibited the highest electron-accepting tendency: its adiabatic electron affinity was 4.07 eV, compared to 2.01 eV for rA^O^G-DNA. Also, rA^O^G-DNA and ^O^GA-DNA demonstrated proton transfer accompanying relaxation of the radical cation state together with charge–spin separation. These findings indicate that rAdo can reshape the electronic landscape of ^O^G-containing CDLs and may influence DNA damage recognition and repair. Such topology-dependent effects may play important parts in genome instability and treatment-associated DNA damage responses.

## 1. Introduction

The seed of life, settled in the genome, is continuously exposed to different extra- and intracellular physical and chemical factors whose activity can lead to different types of DNA damage [1]. It has been estimated that, every hour, 3 × 10^17^ DNA lesions are produced in the human body [2]. Among them, single-strand breaks (SSBs), 7,8-dihydro-8-oxo-2′-deoxyguanosine (^O^G or ^OXO^dGuo) lesions, and apurinic/apyrimidinic sites (AP sites) are the most abundant [1,3]. This prevalence can be higher in some pathological states like cancers, injury or neurodegenerative diseases, or during chemo-, radio- or combined therapy [4]. In the genome of a single eukaryotic cell, it has been estimated that the number of ^O^G lesions ranges from 0.5 to 1.57 per 10^6^ base pairs [5,6] to 33.1 per 10^6^ dGuo [7]. Fortunately, the level of the formed DNA damage is guarded by repair systems: base/nucleotide excision repair (BER, NER), homologous recombination (HR), and non-homologous end joining (NHEJ) [8,9]. BER has been perceived as the first line of cell defence against unwanted damage to genetic information. However, all repair systems are highly effective at removing single or isolated ribonucleotides (rNs) or repairing DNA damage.

**Figure 1:**
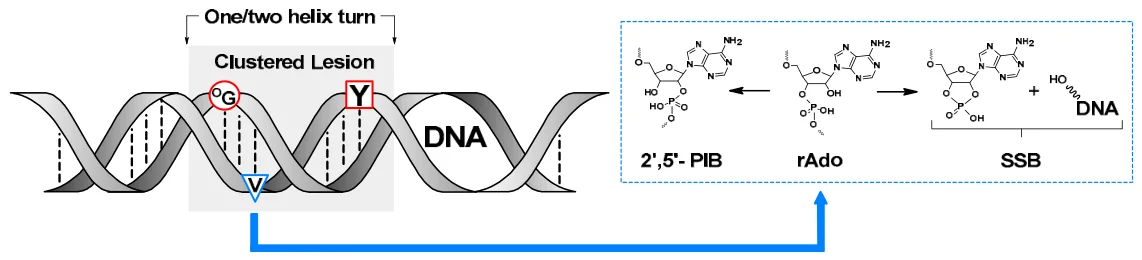
A schematic representation of clustered DNA lesions. The ribonucleotide (**V**) acts as a potential precursor of a single-strand break (**SSB**); **^O^G** indicates 7,8-dihydro-8-oxo-2′-deoxyguanosine; **Y** represents another DNA lesion. **2′,5′-PIB**—2′,5′-internucleotide phosphate bond; **rAdo**—riboAdenosine.

Also, ribonucleotides (rNs) can be inserted into DNA during lagging strand synthesis or during significant deficiency of dNTPs (2′-deoxynucleotide triphosphate) or deoxynucleosides (dNs) [10]. However, rNs can also appear as a result of polymerase errors during leading-strand synthesis [11]. Therefore, replicative polymerases on both leading and lagging strands can incorporate rNMPs because intracellular rNTP concentrations substantially exceed dNTP concentrations. In normal cells, the level of rNTPs (ribonucleosides triphosphate) is around two orders of magnitude higher than that of dNTPs [12]. Unwanted rNs are removed from the genome by ribonucleotide excision repair machinery (RER) [13]. This process is initiated by RNase H2, whose absence can initiate topoisomerase activity [14]. Simply in RNase H2-deficient cells, unrepaired genomic rNMPs can be processed by topoisomerase 1, an alternative pathway associated with mutagenic DNA intermediates [15]. Moreover, the 3′,5′-internucleotide linkage formed by a ribonucleoside is less resistant to changes in pH and temperature than the canonical one, resulting in a greater chance of DNA single-strand break (Figure 1) [16]. The frequency of rNs was estimated to be over 10^6^ moieties per genome in mouse embryonic fibroblast cells with an RNase H2 defect [17]. In the above case, no protein competition has been noted, e.g., MutYH versus RNase H2. A more complicated situation can be observed when complexes of unwanted nucleosides are present in the genome, which may be observed under pathological conditions or during radiotherapy or chemotherapy. The occurrence of at least two DNA lesions within a distance of 12–24 base pairs has been classified as a clustered DNA lesion (CDL) or multiple local DNA damage site (Figure 1) [18,19].

Considering the frequencies of ^O^G and rNs (non-canonical nucleotide for DNA and potential SSB precursor) in the genome, the expected number of their co-occurrences within two turns of the double helix can be estimated at 650 (for a diploid cell with a frequency of 10^6^ rNs per genome [17,20] and 33.1 ^O^G lesions per 10^6^ dGuo [7,21]). This number may reach 1950 in pathological states, when the frequency of rNs in the genome increases threefold [17,22]. This value was calculated using a random genomic point-distribution model based on the binomial distribution, with a Poisson approximation for rare events [23].

Therefore, the presence of rNs in double-stranded (ds-) DNA can influence other DNA damage recognition and repair processes, and vice versa. The aim of the present article is to determine the electronic properties of short ds-DNA segments containing rNs and ^O^G with different mutual distributions, as models of clustered DNA damage.

It should be mentioned that previous studies performed separate examinations of ^O^G-containing clustered oxidative lesions [24,25,26], the electronic consequences of a single embedded ribonucleotide [27], and alternative structural outcomes at a ribonucleotide-containing phosphodiester linkage [28]. To maintain consistency, the same computational formalism was performed in the present studies; however, it has been significantly extended. The method used herein adds the simultaneous placement of rAdo and ^O^G within the model short duplex, with a general sequence d[AGAGA]*d[TCTCT]. It also includes a comparison of four structurally distinct local arrangements, including ^O^G:C pairs, an rA:^O^G mispair, and a G:dA mispair, using one internally consistent set of descriptors.

## 2. Results

### 2.1. Influence of Different Distributions of rAdo and ^O^G on Double-Helix Spatial Geometry

The stability of the ds-DNA structure depends on the mutual spatial organisation of canonical nucleotides and their interaction energies. The double helix is organised by two antiparallel strands connected by hydrogen bonds (HBs) between complementary 2′-deoxynucleosides, linked by phosphodiester internucleotide bonds. Moreover, the π-π interaction between neighbouring base-pair heterocycles is the second “force” of structure stabilisation, equal to HB energy, denoted as stacking interaction [29,30]. Moreover, the ds-DNA in the form proposed by Watson–Crick is submerged in an aqueous solvation shell, with an estimated 27–44 water molecules per nucleoside pair [31]. This gentle geometry is sensitive to any structural changes, such as base modification, the rigidity of the sugar moiety, the number of hydroxyl groups, and the continuity of the sugar–phosphate backbone [32,33,34].

**Figure 2:**
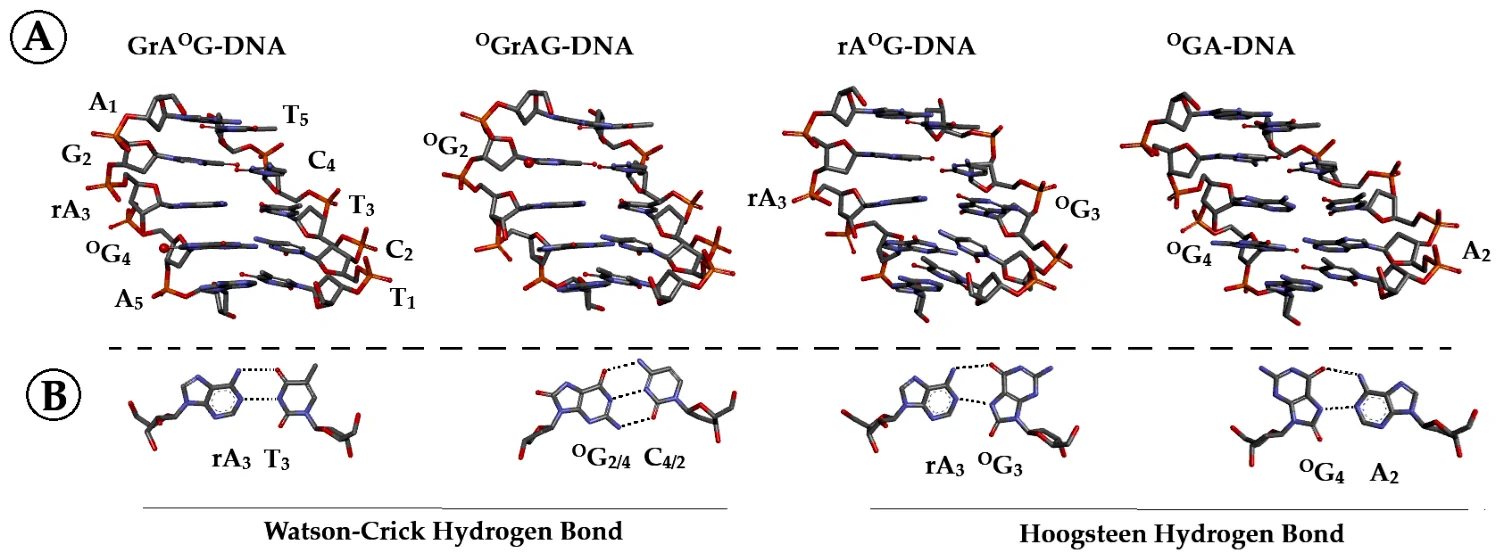
(**A**) Three-dimensional structures of ds-DNAs containing rAdo and ^O^G with different distributions: GrA^O^G-DNA, ^O^GrAG-DNA, rA^O^G-DNA and ^O^GA-DNA; (**B**) the spatial geometries of the nucleoside pairs rA_3_dT_3_ and ^O^G_2/4_C_4/2_ contain Watson-Crick (WC) hydrogen bonds (HBs), whereas rA_3_^O^G_3_ and ^O^G_4_dA_2_ contain Hoogsteen HBs.

Here, the presence of a simple ds-DNA modification, i.e., rAdo insertion (as a ribonucleotide representation), together with the most abundant DNA oxidative damage 7,8-dihydro-8-oxo-2′-deoxyguanosine (^O^G), has been taken into consideration. As rAdo is a non-canonical nucleotide in the DNA, its appearance next to ^O^G can be examined as a multi-damage site. The following notation for the investigated ds-oligonucleotides (ds-oligos) was applied, with the nucleoside sequences given in square brackets: GrA^O^G-DNA d**[**A_1_G_2_rA_3_^O^G_4_A_5_]*d**[**T_5_C_4_T_3_C_2_T_1_**]**, ^O^GrAG-DNA d**[**A_1_^O^G_2_rA_3_G_4_A_5_]*d**[**T_5_C_4_T_3_C_2_T_1_**]**, rA^O^G-DNA d**[**A_1_G_2_rA_3_G_4_A_5_]*d**[**T_5_C_4_^O^G_3_C_2_T_1_**]** and ^O^GA-DNA d**[**A_1_G_2_rA_3_^O^G_4_A_5_]*d**[**T_5_C_4_T_3_A_2_T_1_**]**. The 3D geometries of the ds-DNAs with clustered damage sites are presented in Figure 2. In two of them, rAdo forms a pair with complementary dThd and is surrounded at its 3′-end (GrA^O^G-DNA) or 5′-end site (^O^GrAG-DNA) by an ^O^GdC pair. Additionally, ^O^G may be located on the opposite strand, and may form a pair with rAdo via a Hoogsteen-type hydrogen bond (HB) (rA^O^G-DNA), leaving Watson–Crick HB for all remaining base pairs in the duplex (Figure 2B). Hence, rA^O^G-DNA contains an rA:^O^G Hoogsteen-type mispair, whereas ^O^GA-DNA contains an ^O^G:dA Hoogsteen HB, while GrA^O^G-DNA and ^O^GrAG-DNA contain ^O^G:C pairs adjacent to 5′ or 3′ end of rAdo. These models therefore differ in more than one local structural variable, a point that is explicitly considered in the interpretation below.

The study also examines the influence of rAdo on the properties of the ^O^GdAdo pair linked directly to its 3′-end (^O^GA-DNA), in which the base pair interacts via a Hoogsteen HB. As the ds-oligonucleotides (ds-oligos) are able to transfer charge via stacked heterocycles, the study also considers adiabatic radical cation form, after electron loss, or the radical-anion form, after electron adoption [35]. Based on a comparison of the ds-oligo geometries after electron gain or loss with their neutral forms, it was found that rA^O^G-DNA had the highest resistance to charge-induced changes, with the following root mean square deviation (RMSD [36]) values [Å]: 0.40 for neutral versus anion, 0.38 for neutral versus cation, and 0.49 for anion versus cation (Table 1). In contrast, the change that was most sensitive to charge was ^O^GA-DNA, with respective RMSD values of 1.32, 0.89, and 0.81 Å. Moreover, it has been found that ^O^GrAG-DNA is more sensitive to the addition of an extra electron (0.85 Å) compared to GrA^O^G-DNA (0.40 Å; Table 1).

Interestingly, while electron loss by the system, and the subsequent nuclear relaxation, leads to the formation of the adiabatic radical cation, RMSD analysis found ^O^GrAG-DNA (RMSD: 0.50 Å) to be more structurally resistant to electron loss than GrA^O^G-DNA (RMSD: 0.64 Å). It should be pointed out that the geometric comparison of the adiabatic anion/cation forms with the neutral form yielded RMSD values of 0.17/0.13 Å for native d**[**AGAGA]*d**[**TCTCT**]** and 0.36/0.31 Å for native d**[**AGA^O^GA]*d**[**TCTCT**]** [24]. Further, the comparative analysis between the adiabatic anion and adiabatic cation states shows the following order of structural differences: ^O^GrAG-DNA > ^O^GA-DNA > GrA^O^G-DNA > rA^O^G-DNA. Hence, ^O^GrAG-DNA undergoes the largest structural rearrangement/difference from anion to cation state, and vice versa, i.e., RMSD = 0.95 Å, while rA^O^G-DNA exhibits the easiest (RMSD = 0.49 Å). Therefore, it can be predicted that riboadenosine, as part of a CDL, induces more noticeable or unique structural changes in double-helix geometry than native dAdo.

The results mentioned above from global structural analysis have been derived from RMSD calculations among the cationic, anionic, and neutral forms of the ds-oligos. However, the charge transfer (CT) is highly dependent on the mutual position of nucleobase pairs (BPs) in the formed dimer and the distance between them. These restrictions can be described by two parameters from the DNA standard reference frame, *viz.*, *Rise* (*h*-rise: local base-pair helical parameters) and heterocycle overlap (*OL^BP^*) [37]. As the initiation point of CT depends on the neutral ds-oligo spatial structure, only the three-dimensional geometry of the uncharged ds-DNA in adiabatic neutral geometries was taken into consideration. To avoid nonspecific interactions and geometry fluctuations, the outermost base pairs (A_1_T_5_ and A_5_T_1_) were not considered.

The analysis of heterocycle ring overlap in the base-pair dimers found the following to have the highest *OL^BP^* [Å^2^] values: ^O^G_2_C_4_|rA_3_T_3_ of ^O^GrAG-DNA (11.25), rA_3_T_3_|^O^G_4_C_2_ of GrA^O^G-DNA (8.27), rA_3_^O^G_3_|G_4_C_2_ of rA^O^G-DNA (9.89) and rA_3_T_3_|^O^G_4_A_2_ of ^O^GA-DNA (12.42). It can be postulated that the appearance of 7,8-dihydro-8-oxo-2′-deoxyguanosine in the ds-DNA structure increases the area of heterocyclic-ring overlap with the BP formed by riboadenosine (Table 1). The *Rise* values, i.e., the distance between two neighbouring BPs, were as follows: ^O^G_2_C_4_|rA_3_T_3_ of ^O^GrAG-DNA (2.97 Å), rA_3_T_3_|^O^G_4_C_2_ of GrA^O^G-DNA (2.89 Å), rA_3_^O^G_3_|G_4_C_2_ of rA^O^G-DNA (2.94 Å) and rA_3_T_3_|^O^G_4_A_2_ of ^O^GA-DNA (3.05 Å; Table 1). It should be pointed out that, for the ideal structure of a DNA double helix, A and B forms adopt *Rise* parameters of 2.82 and 3.29 [Å], respectively [37]. This indicates that the observed Rise values remain within the range characteristic of canonical DNA geometries.

Hence, the BP dimers above demonstrated the highest stacking interaction energy (*E_ST_*) values. The *E_ST_* values were calculated for a nucleoside ladder where the phosphate group had been replaced by necessary protons. Importantly, the reported *E_ST_* values are not isolated canonical base-pair-step π-stacking energies. The presented results are in good agreement with previous data [28]. Systematic studies of similar ds-oligos indicate that the interaction energy increases when nucleoside pairs are considered in comparison to the nucleobase ladder [28]. More simply, the *E_ST_* was calculated for complete nucleoside dimers extracted from the duplex after phosphate removal and hydrogen capping; hence, the total interaction energy describes the *E_ST_* between the two nucleoside pairs, including base–base contacts and sugar-mediated contributions, particularly those involving the rAdo 2′-OH group. The highest *E_ST_* was noted when the ^O^G was attached to the 3′-OH group of rAdo [in kcal·mol^−1^]: 38.34 (rA_3_T_3_|G_4_C_2_ of ^O^GA-DNA) and 34.58 (rA_3_T_3_|G_4_C_2_ of GrA^O^G-DNA). Shifting ^O^G to the 5′-end of rAdo or changing the complementary base from T_3_ to ^O^G_3_ leads to an observable reduction in stacking interaction, i.e., 28.75 (rA_3_^O^G_3_|G_4_C_2_ of rA^O^G-DNA) and 28.64 (^O^G_2_C_4_|rA_3_T_3_ of ^O^GrAG-DNA), as shown in Table 1. Moreover, the presence of rAdo in a pair with 7,8-dihydro-8-oxo-2′-deoxyguanosine decreases the *E_ST_* by ~10 kcal·mol^−1^ compared with dAdo^O^G, indicating significant consequences for electron/hole transfer through ds-DNA.

**Figure 3:**
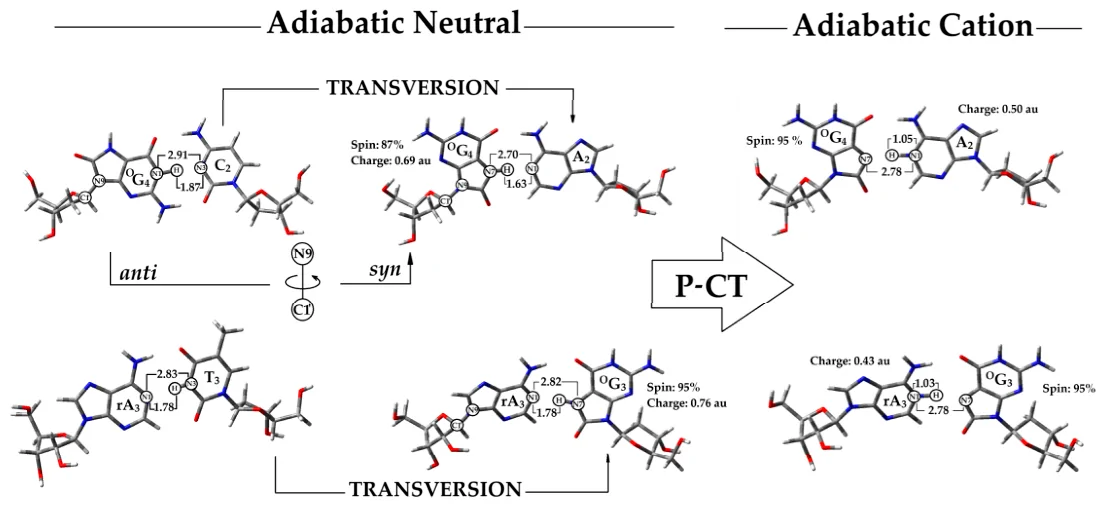
Graphical representation of proton charge transfer (P-CT) after geometry relaxation from the neutral/vertical state to the adiabatic radical cation state for the rA_3_^O^G_3_ nucleoside pair extracted from rA^O^G-DNA and for ^O^G_4_A_2_ as part of ^O^GA-DNA. The *syn* conformers of free 8-OXO-dGuo are more stable than the *anti* confirmers by 0.29 kcal·mol^−1^, and by 2.29 kcal·mol^−1^ when isolated from ^O^GA-DNA and GrA^O^G-DNA, respectively, calculated at the M06-2X/6-31++G** level of theory in the aqueous phase.

It is possible that ^O^G can become incorporated in the genome following post-synthetic modification, leading to GC→TA mutation, or by enzymatic insertion by polymerase, resulting in AT→CG conversion [38]. In both cases, the transversion process is observed during the replication cycle. The formation of the nucleoside pair between 2′-deoxyadenosine (dAdo)/riboadenosine (rAdo) and 7,8-dihydro-8-oxo-2′-deoxyguanosine (^O^G) requires rotation of the guanine moiety around glycosidic (C^1′^-N^9^) bond resulting in the formation of a *syn* conformation and thus a Hoogsteen-type hydrogen bond [39]; this is not possible in the *anti* dGuo form (Figure 3). For the rA_3_^O^G_3_ and ^O^G_4_A_2_ nucleoside pairs, the respective N^1^-N^7^ hydrogen-bond lengths were found to be 2.82 Å and 2.70 Å, while the H^7^-N^1^ distances were 1.78 Å and 1.63 Å. The formation of the adiabatic cation causes migration of proton H^7^ from N^7^ of ^O^G_4_ and ^O^G_3_ to N^1^ of complementary A_2_ or rA_3_, as has been shown in Figure 3. In both cases, the reorganised N^1^-N^7^ hydrogen bond extends over 2.78 Å, while the newly formed H^7^-N^1^ bond was found to be 1.05 Å for ^O^G_4_A_2_ and 1.03 Å for rA_3_^O^G_3_. Compared to the *anti* conformers of free nucleoside 7,8-dihydro-8-oxo-2′-deoxyguanosine (^O^G), the presented *syn* conformers were found to be 0.29 kcal·mol^−1^ more stable when isolated nucleoside was considered and 2.29 kcal·mol^−1^ more stable when taken from GrA^O^G-DNA and ^O^GA-DNA. These values were calculated at the M06-2X/6-31++G** level of theory in aqueous phase.

Two enzymes play key roles in preventing the mutagenesis of ^O^G: OGG1 (8-oxoguanine glycosylase), a bifunctional glycosylase which is on the front line of the BER repair system, and MutYH (adenine DNA glycosylase), which is able to remove the 2′-deoxyadenosine from its pair with ^O^G, but not riboadenosine [40,41,42]. However, while OGG1 and MutYH exhibit synergistic effects in preventing mutagenesis, the rA^O^G mispair may be particularly problematic for repair and may therefore increase mutagenicity [40]. Moreover, the investigated clustered DNA damage may interfere with electron transfer through the double helix, which is essential for DNA damage recognition by MutYH glycosylase. Therefore, the presence of DNA damage clusters containing various distributions of rA and ^O^G can change the ds-DNA electronic properties and therefore allow the appearance of undesirable events, which can lead to genetic material instability.

It is important to note that the influence of the rAdo moiety was assessed not only based on its electronic properties but also from its ribose conformation described by the pseudorotation phase and amplitude. These analyses indicate model- and charge-dependent puckering ranging from ^2^T_1_/^2^T_3_ to ^1^T_3_ (Table 1).

The spatial arrangement of the ribose 2′-OH group was additionally described using the N9–C1′–C2′–O2′ torsion (ε*), which varies substantially among the investigated neutral, cationic, and anionic structures (−66.9° to −115.9°). This data demonstrates that the 2′-OH is conformationally responsive rather than structurally invariant.

Moreover, the spatial orientation of adenine relative to the ribose (discussed for rAdo_3_) was characterised by the glycosidic C4–N9–C1′–O4′ dihedral angle (χ). In contrast, the *syn*/*anti* conformational changes involving 7,8-dihydro-8-oxo-2′-deoxyguanosine and their associated relative energies were independently evaluated, as shown in Figure 3.

Thus, the combined analysis of ribose puckering, ε*, χ, and the relative energies of the *syn*/*anti* ^O^G structures still provides an explicit structural basis for assessing the coupling between the 2′-OH-containing sugar environment and base orientation, despite their assignment to different ONIOM layers [27,28,43,44]. The results of the performed ONIOM studies, with the base-pair ladder treated at the M06-2X/D95** high level (HL) and the sugar–phosphate backbone, including the rAdo 2′-OH group, at the M06-2X/STO-3G low level (LL), are shown below. This represents a computationally tractable description of the electronically relevant π-stacked bases while retaining the structural response of the complete backbone [27,28]. The standard Gaussian ONIOM strategy was used. The HL and LL distributions are shown in Figure S1.

### 2.2. The Ds-DNA Charge and Spin Distribution Induced by the Presence of a Clustered DNA Lesion

The canonical DNA double helix is constructed from complementary nucleoside pairs, with each strand retaining integrity through internucleotide phosphodiester bonds. As noted by Barton et al., this structure of ds-DNA allows for charge transfer over long distances, although the presence of structural changes such as lesions can disrupt this flow. Moreover, DNA damage significantly alters the charge distribution along double-stranded oligonucleotides. To the author’s knowledge, the influence of clustered DNA damage containing ribonucleosides on charge and spin distribution has not been the subject of investigation. For this purpose, the charge model 5 (CM5), based on the Hirshfeld methodology, has been applied at the M06-2X/6-31++G** level of theory in the condensed phase (water, dielectric constant ε = 78.36). The CM5 correction increases the physically meaningful charge separation and was parameterised to improve the reproduction of molecular dipole moments. Consequently, CM5 charges are often more suitable for describing molecular polarity and electrostatic interactions while retaining the favourable numerical stability and relatively weak basis-set dependence associated with classical Hirshfeld population analysis [45]. Moreover, due to the nature of charge adoption by the oligonucleotide submerged in the aqueous shell, the nonequilibrated and equilibrated solvent–solute interactions were considered. This approach gives a more accurate view of the initial point of charge and spin formation, with their further rearrangement with regard to the spatial geometry of the ds-DNA.

**Figure 4:**
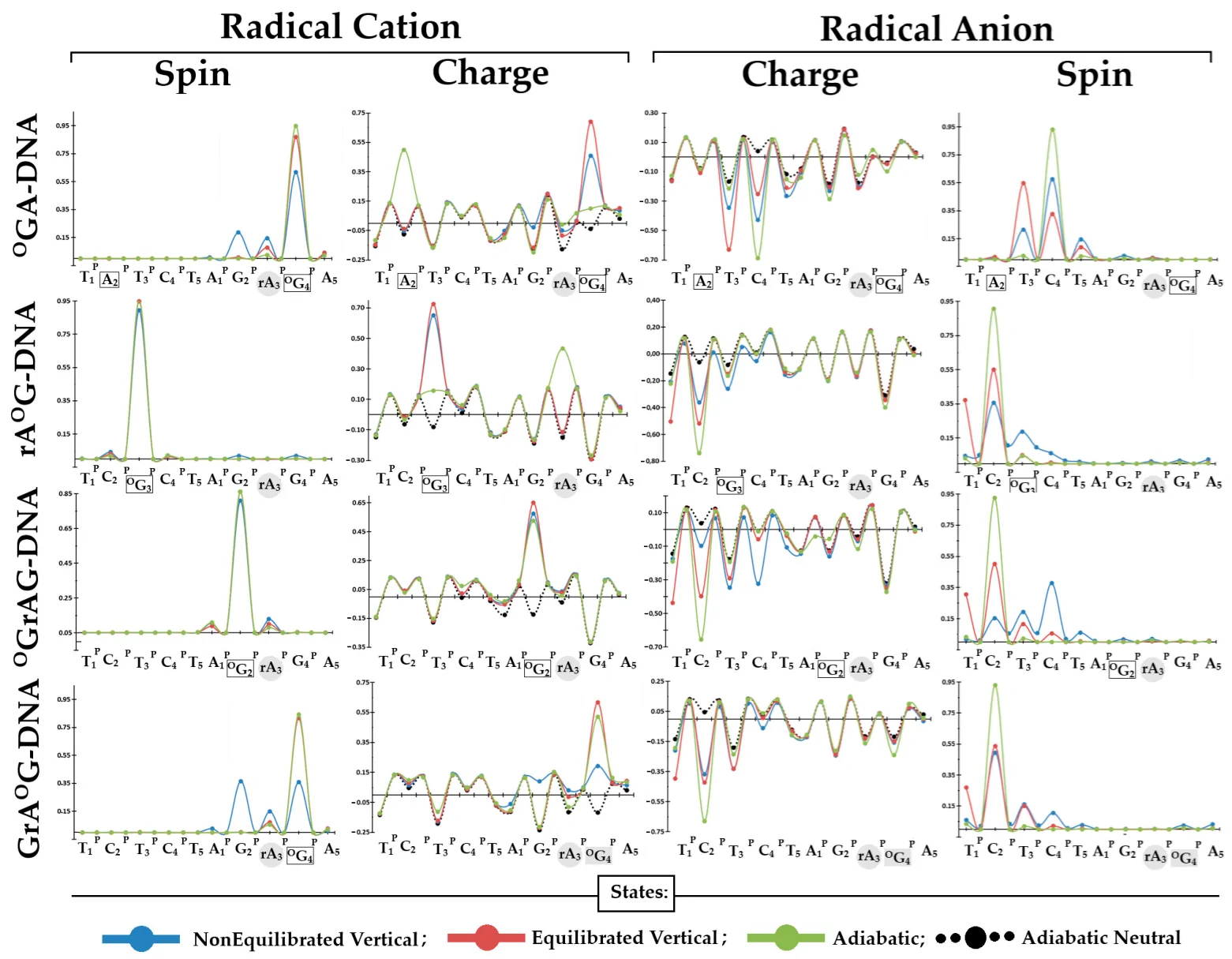
Spin and charge distribution in the discussed ds-oligos: ^O^GA-DNA, rA^O^G-DNA, ^O^GrAG-DNA and GrA^O^G-DNA obtained at the M06-2X/6-31++G** level of theory in the aqueous phase using Hirshfeld and charge model 5 methodology, respectively. The raw data are given in supplementary materials Table S1.

To obtain a holistic view of the above, the whole ds-DNA structure, including the sugar–phosphate backbone and base-pair ladder, has been subjected to analysis. The negative charges of the phosphate groups were formally neutralised by protonation. Electron loss by the ds-oligo resulted in the formation of a radical cation. Depending on solvent–solute interaction, the vertical state should be considered firstly as a nonequilibrated (NE) vertical state, which, after solvent reorganisation, yields the equilibrated (EQ) vertical state and, after further relaxation, the adiabatic cation state. Spin-density analysis of the discussed ds-oligos (Figure 1) found that, in the case of ^O^GrAG-DNA and rA^O^G-DNA, the unpaired electron is almost completely located on the ^O^G (~90%) moiety, regardless of the nonequilibrated or equilibrated solvent–solute interaction and molecular-geometry relaxation (Figure 4 and Table S1 in supplementary materials).

Surprisingly, in the nonequilibrated state, the appearance of 7,8-dihydro-8-oxo-2′-deoxyguanosine at the 3′-end side of rAdo disperses the spin density across the G_2_, A_3_ and ^O^G_4_ moieties, as follows: 40%, 16%, 39% (GrA^O^G-DNA) and 19%, 15%, 62% (^O^GA-DNA; Figure 4). A final accumulation of almost 90% was noted on the ^O^G_4_ nucleoside after solvent and molecular-geometry relaxation. The situation becomes similar when the radical cation distribution is considered. The positive charge was found mainly on the ^O^G_4_, ^O^G_3_, and ^O^G_2_ moieties when both vertical states, *viz.*, NE and EQ, were considered independently (Figure 4). The situation becomes different when the adiabatic radical cation state is achieved. In ^O^GrAG-DNA and GrA^O^G-DNA, the positive charge remains mainly on 7,8-dihydro-8-oxo-2′-deoxyguanosine, whereas in ^O^GA-DNA and rA^O^G-DNA, it is found on the complementary dAdo (A_2_: 0.5au) and rAdo (rA_3_: 0.43au). Relaxation from the vertical to the adiabatic radical cation state results in proton transfer (Figure 3) accompanied by a charge–spin separation process. Hence, it can significantly influence the electronic properties of the ds-oligonucleotide and thus damage recognition by glycosylases such as MutYH.

The appearance of the excess electron in the double helix causes the NE vertical anion formation, which, after solvent relaxation, gives the EQ vertical anion state. The further response of the molecule’s atoms (rearrangement) to the additional electron yields the adiabatic radical anion. Following the appearance of this negative charge, each of the discussed ds-DNA arrangements responded differently **(**Figure 4). In the ^O^GA-DNA, charge localisation initially occurred mainly on the C_4_ (58%/−0.43 au) nucleoside, followed by transfer to the T_3_ (55%/−0.63 au) subunit and finally localisation again on C_4_ (93%/−0.69 au). In ^O^GrAG-DNA, the attachment of ^O^G to the 5′ hydroxyl group of rAdo leads to electron localisation in the NE vertical state, mainly on C_4_ (38%/−0.32 au), with subsequent redundant electron transfer toward C_2_ (50%/−0.40 au) in the EQ vertical form, finally settling on the C_2_ (92%/−0.66 au) moiety after achieving an adiabatic radical-anion state (Figure 4). In both remaining ds-oligonucleotides, GrA^O^G-DNA and rA^O^G-DNA, the spin and negative charge are initially slightly dispersed over the nucleosides adjacent to the C_2_ moiety and, after solvent–solute relaxation and subsequent ds-oligo relaxation, become localised on C_2_. These results elucidate the pivotal role of ribonucleotides, in this case rAdo, on the electron transfer through the double helix with a CDL unit. This can further influence DNA damage recognition processes by glycosylases and also other protein communication via the ds-DNA nanowire.

It should be pointed out that physiological DNA is a polyanion. Hence, the protonation of the internucleotide phosphate bond used in the current studies should be regarded as a formal computational neutralisation of the finite DNA model. The above is a representation of the physiological protonation state or as an equivalent of explicit Na^+^ counterion neutralisation. However, protonated phosphate/nucleotide models have a well-established place in quantum-chemical (theoretical) studies of DNA. It should be mentioned here that Leszczynski investigated differently protonated deoxyribonucleotides in their calculations of electron attachment and DNA strand chemistry, followed by protonated nucleotide models [46,47]. In contrast, similar calculations on phosphate-containing cytidine oligomers demonstrated the formation of base-centred radical anions [48]. It has been extensively demonstrated (Kumar, Sevilla and co-workers) [27,43,49] that the electronic states associated with oxidation/reduction and the resulting hole/electron or spin density are predominantly localised within the nucleobase/base-pair subsystem, particularly at guanine-containing sites. In contrast, the phosphate backbone is not the principal electronic pathway governing charge migration. However, the negative charges of the internucleotide phosphate bond and explicit counterions modify the electrostatic environment and may consequently affect absolute orbital energies, ionisation potentials and electron affinities. Therefore, the above approach was used to analyse relative changes in charge localisation and CT within our consistently treated series, rather than to quantitatively reproduce the complete electrostatics of physiological, solvated polyanionic DNA [28,50].

### 2.3. Fukui Reactivity Index of ^O^GrAG-DNA, GrA^O^G-DNA, ^O^GA-DNA and rA^O^G-DNA

The spin and charge analyses presented above disclose the variety of their distribution patterns in the light of clustered DNA damage composition (Figure 2). Due to the plethora of reactive molecules present in the genome environment, including radicals, nucleophiles/electrophiles, the identification of the most reactive atoms in the investigated ds-DNA containing a multi-damage site built around rAdo is of particular interest. For this purpose, the Fukui reactivity index has been considered.

As given by conceptual density functional theory (CDFT), the Fukui function *f*(r) provides a local quantum-chemical measure of the response of the electron density to a change in electron number at a fixed external potential [51]. In the present study, condensed Fukui functions were evaluated at the fixed neutral geometry using the equilibrated CPCM representation [52]. The function is formally defined as the derivative of the electron density with respect to the number of electrons, with ρ(r) denoting the electron density and N the total electron number. The above can be noted as follows:

*f*(r) = (∂ρ(r)/∂N)_v_(r).

As the derivative is evaluated from opposite sides at an integer N, practical calculations usually employ finite-difference expressions for electron addition and electron removal to and from the discussed systems, as follows: *f^+^*(r) = ρ_n+1_(r) − ρ_n_(r) and *f^−^*(r) = ρ_n_(r) − ρ_n−1_(r). The Fukui function with a positive index, *f^+^*, predicts the atoms/fragments that preferentially accept additional electron density, i.e., electrophilic sites that may be susceptible to nucleophilic attack. In contrast, the Fukui function, noted as *f^−^*, indicates the part of the molecule from which an electron is preferentially removed, i.e., nucleophilic sites susceptible to electrophilic attack. Within the frontier molecular orbital approximation, *f^+^* and *f^−^* can be related approximately to the LUMO and HOMO densities, respectively, providing a direct orbital interpretation of local reactivity, i.e.,

*f^+^*(r) ≈ |ψLUMO(r)|^2^ and *f^−^*(r) ≈ |ψHOMO(r)|^2^

The radical Fukui function *f*^0^ can be defined as the average of *f^+^* and f^−^ and describes the relevance of a point of a molecule to radical processes, noted as follows:

*f*^0^(r) = ½[*f^+^*(r) + *f^−^*(r)].

The dual-descriptor Δ*f*(r), i.e., the difference between the electrophilic and nucleophilic Fukui functions, combines both tendencies in a single local quantity and can be expressed as a second finite difference in the electron density [53].

Δ*f*(r) = *f^+^*(r) − *f*^−^(r) = ρ_n+1_(r) − 2ρ_n_(r) + ρ_n−1_(r).

For atom-condensed analyses, the Fukui indices may be evaluated from net atomic charges Q_k_ obtained for the N − 1 (cation), N (neutral), and N + 1 (anion) electron vertical states, i.e., at the same nuclear spatial geometry, using the following charge-difference notation [54].

*f_k_^+^* = Q_k_(N) − Q_k_(N + 1)

*f_k_^−^* = Q_k_(N − 1) − Q_k_(N)

*f_k_*^0^ = ½[Q_k_(N − 1) − Q_k_(N + 1)]

Δ*f_k_* = *f_k_^+^* − *f_k_^−^* = 2Q_k_(N) − Q_k_(N + 1) − Q_k_(N − 1)

Accordingly, Δ*f_k_* > 0 denotes a predominantly electrophilic atomic site, whereas Δ*f_k_* < 0 denotes a predominantly nucleophilic site, and Δ*f_k_* ≈ 0 indicates neutral character. Condensed values depend on the population scheme used to define the Q_k_ charge model. In this article, the charge distribution was calculated at the M06-2X/6-31++G** level of theory in the aqueous phase (C-PCM solvation model) using charge model 5 based on Hirshfeld population methodology [45]. The calculated values of the Fukui functions are presented in Table 2. Only results with absolute values higher than or equal 0.05 were taken into consideration. Due to the large number of heavy atoms in the discussed system, absolute values lower than 0.05 are not shown; the crude data are presented in Table S2.

Under careful analysis, the different structures exhibit differing abilities to undergo nucleophilic attack depending on CDL structure. For GrA^O^G-DNA, the highest positive Δ*f_k_* value (i.e., 0.11) was found for the C^6^ carbon of 2′-deoxycytidine (dC_2_), whereas negative values were found for N^2^ (−0.10) and C^5^ (−0.09) of 7,8-dihydro-8-oxo-2′-deoxyguanosine ^O^dG_4_. The ^O^G shift from the 3′-end of rAdo (rA3) to the 5′-end yields ^O^GrAG-DNA where the electrophilic centre is located at the C^6^ carbon (0.16) of the dC_2_ nucleoside, while the highest nucleophilic value was assigned to N^2^ (−0.13), with a moderate value at C^5^ (−0.10) of the ^O^dG_2_ lesion.

The formation of a non-canonical base pair between rAdo (rA_3_) and ^O^G_3_ containing Hoogsteen hydrogen bonds gives the following dual-descriptor (Δ*f_k_*) for rA^O^G-DNA: the highest positive values were found for N^4^ (0.15) and C^6^ (0.14) of the dC_2_ subunit, with equal negative values (−0.09) observed for N^2^, O^6^, C^5^, and O^8^ of ^O^G_3_ complementary to rAdo (rA_3_). In ^O^GA-DNA, the formation of the ^O^G_4_A_2_ 2′-deoxynucleoside pair below the 3′-end of riboadenosine results in a Δ*f_k_* value of 0.11 for C^6^ of thymidine T_3_, the atom most susceptible to nucleophilic attack, whereas O^8^ (−0.12) and N^2^ (−0.09) of ^O^dG_4_ were found to be most susceptible to electrophilic attack.

These findings are supported by the *f**_k_***^0^ calculation, indicating a tendency toward radical attack. The following highest values were assigned to: GrA^O^G-DNA, i.e., C^6^ of dC_2_ (0.06) and N2 (0.06) of ^O^dG_4_; ^O^GrAG-DNA—C^6^ of dC2 (0.05) and N_2_ (0.06) of ^O^dG_2_; rA^O^G-DNA—C^6^ (0.06) of dC_2_ and 0.06 for O^8^, N^2^ of ^O^dG_3_; and ^O^GA-DNA—C^6^ (0.06) of T_3_ and O^8^ (0.06) of ^O^dG_4_ (Table 2). It can be seen that the distribution of 7,8-dihydro-8-oxo-2′-deoxyguanosine around riboadenosine yields different results and can influence charge transfer in different ways. Moreover, it can influence further ^O^G rearrangement toward subsequent lesions, such as spiroiminodihydantoin. Additionally, the discussed clustered lesion shows the ability to predict the directionality of pyrimidine lesion formation (it may identify sites potentially susceptible to subsequent chemical reactions), as shown in (Table 2).

### 2.4. The Global Reactivity Descriptors of ^O^GrAG-DNA, GrA^O^G-DNA, ^O^GA-DNA and rA^O^G-DNA

Due to number of atoms in the investigated ds-pentamers, the values of the Fukui descriptors presented above assigned for each atom have been found to be small, making the interpretation of the results difficult. Hence, many studies have used short ds-DNA systems, e.g., double-stranded trimers or pentamers. The stability and reactivity of the macromolecules can be well-predicted by the energy of frontier orbitals, HOMO (highest occupied molecular orbital) and LUMO (lowest unoccupied molecular orbital), according to Koopmans’ theorem approximation. These properties are closely related to the ability of the molecule to undergo electron loss or gain.

According to the above theory, the vertical ionisation potential (VIP) and electron affinity (VEA) can be estimated from the energies of the frontier orbitals. Thus, the electronic properties and global reactivity descriptors of a molecule can be estimated in the vertical radical-anion or cation mode. This calculation should also consider the nonequilibrated (NE) and equilibrated (EQ) solvent–solute interactions, due to the interaction between the orbital and the electrostatic potential. This strategy was possible and reasonable because the geometry of the investigated macromolecule was frozen. The positions of the atoms did not change during the rearrangement from the adiabatic neutral state to the vertical radical cation/anion form. It should be pointed out that only ds-DNA orbital rearrangement took place during the formation of the nonequilibrium vertical state.

Therefore, the following global reactivity descriptors can be calculated in the following three modes based on the vertical energies: chemical hardness (η = 0.5[VIP − VEA] or η = 0.5[E^LUMO^ − E^HOMO^]) and softness (S = 1/η), global electrophilicity index (ω = μ2/2η), and electronic chemical potential (μ = −0.5[VEA + VIP] or μ = 0.5[E^LUMO^ + E^HOMO^]). All results are given in Table 3.

Based on Koopmans’ theorem, the highest hardness and the lowest softness were found for rA^O^G-DNA. Surprisingly, ^O^GA-DNA demonstrated the lowest electronic chemical potential (−3.84 eV) among the discussed short ds-DNAs and the highest global electrophilicity index (2.38 eV). It should be pointed out that these findings were determined based on the HOMO and LUMO energies assigned for the geometry of the neutral macromolecule in the ground state [55]. Therefore, this calculation does not consider any possible rearrangement of the remaining orbitals.

The situation becomes different when the vertical ionisation potential and electron affinity are considered, according to density functional theory, described in the Materials and Method section. In the nonequilibrated and equilibrated states after electron loss or gain, the molecular orbitals adapt to the new electronic situation (electrostatic potential) and change their energy landscape. In this mode, which is more related to the “natural” situation, rA^O^G-DNA exhibits the highest chemical hardness *η* = 3.11 eV. Opposite to the above, ^O^GA-DNA was the most reactive (highest softness), S = 0.35 eV^−1^, with the lowest electronic chemical potential (μ = −3.82) and the highest global electrophilicity index (ω = 2.53). Hence, ^O^GA-DNA has a stronger tendency to accept an electron than rA^O^G-DNA, which had the highest value, i.e., μ = −3.65 eV.

Following solvent environment relaxation, the system enters an equilibrated solvent–solute integration state. However, the same patterns for hardness and softness were observed as for the nonequilibrated mode: *η*: rA^O^G-DNA > GrA^O^G-DNA > ^O^GrAG-DNA > ^O^GA-DNA; *S*: ^O^GA-DNA > ^O^GrAG-DNA ≥ GrA^O^G-DNA > rA^O^G-DNA. Interestingly, a different order of electronic chemical potential was observed, i.e., *μ*: ^O^GrAG-DNA > rA^O^G-DNA > GrA^O^G-DNA > ^O^GA-DNA. It should be pointed out that the solvent–solute [56] equilibration increased rA^O^G-DNA electrophilicity by 0.8 eV up to 2.95 eV. According to Pérez’s prediction, strong electrophiles are characterised by an ω value above 1.5 eV, moderate ones between 1.5 and 0.8 eV and weak ones below 0.8 eV [28,38,39]. On the empirical electrophilicity scale commonly used for organic molecules, these ω values would fall in the range classified as strong electrophilicity; this classification should be regarded as qualitative for oligonucleotides [57]. As all studied ds-DNAs have ω values greater than or equal to 2.15 eV irrespective of calculation mode, they appear to be strong electrophiles. Hence, it can be postulated that rAdo can modulate the reactivity of short parts of the genome, changing the molecular properties of attached DNA damage (Table 3).

### 2.5. The Influence of Different Mutual Distributions of rAdo and OG on the Electronic Properties of Ds-DNA

Charge transfer (electron or hole) through the double helix yields a local radical-anion or cation dispersed area in the initial step, which then migrates and settles at the predisposed point. During this period, the spin and charge distributions are influenced by solvent–solute interactions (Figure 4). The one haploid human genome contains 3 × 10^9^ nucleoside pairs, which can form a specific mosaic of the most predisposed sequences for one-electron oxidation or reduction, depending on their structure. The above can be predicted by vertical ionisation potential (VIP) and vertical electron affinity (VEA) in two modes: nonequilibrated (NE) and equilibrated (EQ) solvent–solute interaction. The subsequent [58] nuclear rearrangement forced by electron gain or loss allowed the final adiabatic electron affinity (AEA) or ionisation potential (AIP) calculation.

**Figure 5:**
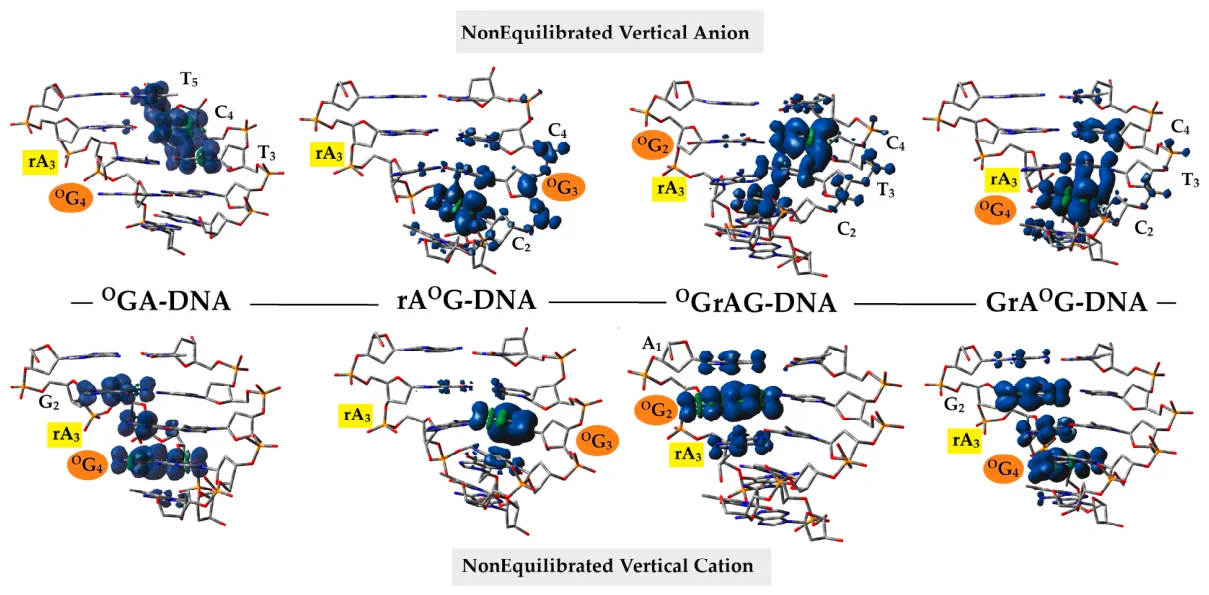
Graphical representation of the spin distribution in the vertical anion and vertical cation forms of the discussed ds-DNA in the nonequilibrated solvent-solute interaction mode (NE), calculated at the M06-2X/6-31++G** level of theory in the aqueous phase.

The charge-transfer properties of the DNA influence the activity of the different proteins involved in the repair and replication processes [56,58,59,60,61], e.g., DNA lesion identification. This has been well-investigated experimentally for isolated DNA lesions [62,63,64], like ^O^G, and theoretically [65]. However, the situation becomes complicated when the CDL has been taken into consideration. Here, for the first time, the ribonucleoside, containing the additional 2′-OH group, was considered as non-canonical DNA modification (lesion), i.e., a moiety of a clustered DNA lesion. The electronic parameters mentioned above were calculated at the M06-2X/6-31++G** level of theory in the aqueous phase for complete ds-oligonucleotides containing a sugar–phosphate backbone and nucleobase-pair ladder, and for extracted nucleoside pairs, instead of base pairs; this approach allowed the influence of the 2′-OH group on the electronic parameters to be estimated. As shown in Figure 4 and Figure 5, the spin distribution within the ds-DNA structure, when the NE vertical radical-anion and cation states are considered, depends on the relative positions of rAdo and ^O^G. Hence, the discussed ds-oligos have shown the different influences on initial electron gain or loss. The lowest ^NE^VIP was found for ^O^GrAG-DNA (6.60 eV), where the ^O^G is attached to the 5′-end of rAdo, while, surprisingly, shifting the ^O^G to the 3′-end causes the ^NE^VIP to increase to 6.78 eV. The highest ^NE^VEA was assigned to ^O^GA-DNA (0.94 eV), which contained the ^O^G_4_A_2_, non-canonical nucleoside pair, below the 3′-OH of riboadenosine. The lowest ^NE^VEA was noted for rA^O^G-DNA (0.55 eV); this indicates the significant influence of rAdo on electron gain by the ds-oligo. The solvent relaxation and achievement of an equilibrated solvent–solute interaction state led to the following order of ^EQ^VIP: ^O^GrAG-DNA < ^O^GA-DNA < rA^O^G-DNA < GrA^O^G-DNA and ^EQ^VEA: ^O^GA-DNA > GrA^O^G-DNA > rA^O^G-DNA > ^O^GrAG-DNA (Table 4). Further structural relaxation leads to proton charge transfer in ^O^GA-DNA and rA^O^G-DNA (Figure 3), which reduces AIP to 3.39 eV and 5.21 eV, respectively.

Most importantly, the presence of rAdo decreases the ability of ds-oligo to lose an electron compared with dAdo, as reflected by an AIP value higher by 1.82 eV. Moreover, the ^O^G position with regard to rAdo also influences the result: the AIP of ^O^GrAG-DNA (5.95 eV) is lower than that of GrA^O^G-DNA (6.11 eV). However, the above is in good agreement with Sevilla’s studies, performed for native short ds-DNA [66,67,68].

Following structure relaxation to the ground state, after electron adoption, the highest AEA value was observed for ^O^GA-DNA (4.07 eV), which is twice that for rA^O^G-DNA (2.01 eV). Taken together, i.e., these results and the AIP analysis predict that rAdo has a modulated role on the electronic properties of the double helix and, therefore, on charge transfer when the clustered DNA lesion is considered.

These findings are supported by “nucleoside pair by nucleoside pair” analysis for each of the discussed ds-oligonucleotides. However, the lack of a solvation layer in the intramolecular double-helix part limited the usefulness of the nonequilibrated mode for this study. Therefore, only the equilibrated (EQ) mode was used for further calculations.

The comparative analysis of vertical ionisation potentials found that the nucleoside pairs (NPs) containing the ^O^G moiety exhibited almost identical ^EQ^VIP values, i.e., in the range 5.96–5.99 eV, which are the lowest among all considered NPs. This is in good agreement with previous data of dGuo and its oxidised derivative: 7,8-dihydro-8-oxo-2′-deoxyguanosine, found by Schuster and Saito [25,27,63,65]. Moreover, the spin concentration was mainly observed on the ^O^G moiety (Figure 3) of the discussed ds-oligos, independent of hydrogen-bond type and complementary nucleoside.

The geometry relaxation to the cation ground state emphasises the influence of the presence of rAdo at the clustered DNA lesion site. The lowest AIP was found for the ^O^G_4_A_2_ pair in ^O^GA-DNA, and was 0.16 eV lower than that assigned to rA_3_^O^G_3_ in rA^O^G-DNA. It should be pointed out that, in both cases, proton charge transfer was observed (Figure 3 and Figure 4). Moreover, the AIP of the ^O^G_2_C_4_ nucleoside pair attached to the 5′-end of rAdo (^O^GrAG-DNA; 5.61 eV) was 0.37 eV higher than that assigned to ^O^G_4_C_2_ located on the opposite 3′-end side (GrA^O^G-DNA; 5.24 eV).

The excess electron attachment to the ds-DNA has varying consequences on the ^EQ^VEA of the nucleoside pairs present in the discussed ds-DNA. Among all NPs derived from the ds-oligonucleotides, the highest ^EQ^VEA value was found for rA_3_T_3_ of ^O^GA-DNA (1.65 eV), with values ranging from 1.57 to 1.60 eV noted for G_4_C_2_ (^O^GrAG-DNA), ^O^G_4_C_2_ (GrA^O^G-DNA), and G_4_C_2_ (rA^O^G-DNA). It should be mentioned here that no predisposed site for the migrating electron has been identified in the ds-oligo at the 5′-end of rAdo. Further, relaxation of the ^O^GA-DNA structure shifts the point of highest electron affinity from rA_3_T_3_ in the vertical state to G_2_C_4_ in the adiabatic state; this NP also demonstrated the highest adiabatic electron affinity, i.e., 2.33 eV, among all discussed nucleoside pairs (Table 4). Additionally, for the remaining ds-oligonucleotides, increases in electron affinity were observed after the adiabatic radical-anion state achievement, which was reached for the same nucleoside pairs as those discussed in the equilibrated vertical mode. The following AEA values have been calculated: 2.07 eV for G_4_C_2_ (^O^GrAG-DNA), 2.01 eV for G_4_C_2_ (rA^O^G-DNA), and 1.96 eV for ^O^G_4_C_2_ (GrA^O^G-DNA). It should be pointed out that, contrary to ^O^GA-DNA, all the discussed NPs are located at the 3′-end of rAdo.

It should be pointed out that careful structural analysis of the neutral, anion and cation forms of ^O^GA-DNA confirmed no additional molecular dissociation or further proton migration beyond the protonation/proton-transfer pattern (Figure 3) in the geometry ground state. The ionisation potential of adiabatic ^O^GA-DNA was 3.39 eV and the electron affinity 4.07 eV. These are interpreted as the combined consequence of lesion position, base-pairing identity and local sequence context, and are consistent with the fact that DNA charge transfer is influenced by stacking and sequence [44,69]. Due to their structural differences, the obtained results should be interpreted in terms of the corresponding fully relaxed ionised structures rather than by direct comparison with the vertical ionisation or electron-attachment parameters of canonical DNA. Structural relaxation, protonation state and charge localisation can substantially influence the calculated adiabatic electronic properties (AIP, AEA), as demonstrated previously for modified ds-oligonucleotides [27,28,43,44,70,71].

## 3. Discussion

Ribonucleotides are the most abundant non-canonical nucleotides transiently incorporated into genomic DNA, and under normal conditions, their incision is initiated predominantly by RNase H2-dependent ribonucleotide excision repair [56]. Their biological consequences, however, become considerably more complex when a ribonucleotide occurs in the vicinity of other DNA lesion (Figure 1). This distinction between an isolated rN and a DNA damage site is particularly relevant for 7,8-dihydro-8-oxo-2′-deoxyguanosine: previous biochemical studies disclosed that the simultaneous presence of an incorrect sugar and an oxidised base can alter the interaction between repair pathways [60]. Cilli et al. demonstrated that RNase H2 can incise rAdo paired with ^O^G, whereas MutYH retains binding to the ^O^GrA nucleoside pair but loses its glycosylase activity toward rAdo; importantly, MutYH binding also reduces subsequent RNase H2 incision [40]. Thus, the biological behaviour of a genome site containing ^O^G and rN cannot be predicted simply from the repair properties of these two isolated modifications. The results presented here extend this concept from the effect of such damage on enzymatic processing to include the underlying structural and electronic properties of the lesioned DNA double helix.

The most significant finding is that the effect of rAdo and ^O^G is strongly dependent on their mutual spatial distribution/arrangement (Figure 2). The four investigated ds-oligonucleotides did not respond uniformly to electron attachment or removal. rA^O^G-DNA showed the smallest charge-induced structural rearrangement, with RMSD values of 0.40 Å for the neutral versus anion comparison and 0.38 Å for the neutral versus cation comparison. In contrast, ^O^GA-DNA was substantially more sensitive, with corresponding values of 1.32 and 0.89 Å. The orientation of ^O^G relative to rAdo has been found also important: ^O^GrAG-DNA underwent considerably greater reorganisation following electron attachment than GrA^O^G-DNA (0.85 versus 0.40 Å). The opposite tendency was observed following electron loss (Table 1). Such position dependence is consistent with the general behaviour of clustered DNA lesions, for which the biological consequences of the secondary lesion depend not only on its chemical nature [60,61,62,63].

The theoretical results presented above are well-supported by experimental studies of cluster DNA damage containing ^O^G as the substrates, which demonstrated that the presence and position of a neighbouring lesion can alter OGG1-dependent processing, e.g., BER [62,64]. Therefore, rAdo should not be considered merely as an additional chemically labile nucleotide within a CDL, but as a structural component capable of modifying the properties of the entire local damage site.

This interpretation is supported by the presented stacking interaction energies (*E_ST_*); see Table 1. The largest *E_ST_* (given in kcal·mol^−1^) values were found when the relevant ^O^G-containing moiety was positioned at the 3′ side of rAdo, reaching 38.34 in ^O^GA-DNA and 34.58 in GrA^O^G-DNA, compared with 28.75 for rA^O^G-DNA and 28.64 for ^O^GrAG-DNA. Most importantly, the stacking interaction for the rA_3_^O^G_3_ nucleoside pair was 10 kcal·mol^−1^ lower than for the corresponding ^O^G_4_dA_2_. This difference is potentially relevant for DNA-mediated charge transport because efficient migration of an electron hole through the double helix requires effective electronic coupling between stacked nucleobases and is highly sensitive to perturbation of the π-π stack. DNA-mediated charge transport has been demonstrated to play a role in lesion detection and in communication between DNA repair enzymes for proteins containing redox-active [4Fe–4S] clusters [65,69,72]. Consequently, replacement of dAdo by rAdo within, or adjacent to, an 7,8-dihydro-8-oxo-2′-deoxyguanosine containing lesion may alter charge migration, even in the absence of a gross disruption of the overall double-helical structure.

The calculated spin and charge distributions demonstrate that these structural changes are accompanied by substantial electronic consequences (Figure 4, Table S1). In ^O^GrAG-DNA and rA^O^G-DNA, approximately 90% of the unpaired electron (represented by spin density) following one-electron oxidation is ultimately localised on the ^O^G moiety. This observation is consistent with previous theoretical analyses indicating that 7,8-dihydro-8-oxo-2′-deoxyguanosine constitutes an energetically preferred site for radical cation (hole) localisation in DNA [43]. In contrast, in GrA^O^G-DNA and ^O^GA-DNA, the initial nonequilibrated spin distribution is considerably more delocalised over G, A or rA and ^O^G before solvent and nuclear relaxation; it eventually becomes localised predominantly on ^O^G. Thus, 7,8-dihydro-8-oxo-2′-deoxyguanosine remains an efficient terminal hole trap. However, the initial distribution and migration pathway of the positive charge depend strongly on the position of rAdo within the clustered lesion. This distinction may be biologically relevant because DNA-mediated redox signalling by **[**4Fe–4S**]** repair proteins is particularly sensitive to base stacking perturbations and charge transport infection [65,69].

The present results of theoretical studies have not directly demonstrated altered activity of MutYH or other DNA repair protein. Nevertheless, they provide a physicochemical basis for the hypothesis that different vicinal distribution of rAdo and ^O^G may yield electronically distinguishable DNA substrates. An additional and particularly striking effect appears after structure relaxation of the vertical radical cation state. In rA^O^G-DNA and ^O^GA-DNA, proton migration from ^O^G toward the complementary rAdo or dAdo is accompanied by charge–spin separation (Figure 3). Proton-transfer and proton-coupled charge-transfer processes are well-established determinants of the stability and subsequent chemistry of one-electron-oxidised DNA base pairs [67]. The present results predict that this process can be significantly modified by the insertion of rAdo into the local environment of ^O^G. This is particularly evident from the identified adiabatic ionisation potentials (Table 4). AIP decreases to 3.39 eV for ^O^GA-DNA, compared with 5.21 eV for rA^O^G-DNA, 5.95 eV for ^O^GrAG-DNA and 6.11 eV for GrA^O^G-DNA. Moreover, comparison of the ^O^G_4_:A_2_ and rA_3_^O^G_3_ environments shows that the presence of rAdo increases the AIP by 1.82 eV, indicating that the rAdo-containing system is far less likely to undergo electron loss. Importantly, this difference cannot be attributed solely to the intrinsic red-ox properties of ^O^G. It results from the combined effects of base pairing, the additional 2′-OH group and the topology of the cluster DNA damage site.

Electron attachment was also dependent on ds-DNA sequence and its local spatial structure [68]. The highest ^NE^VEA value was observed for ^O^GA-DNA (0.94 eV), while complete structural relaxation produced an AEA of 4.07 eV (Table 4). This value is approximately twice that obtained for rA^O^G-DNA (2.01 eV) and markedly higher than those calculated for GrA^O^G-DNA (1.46 eV) and ^O^GrAG-DNA (1.34 eV). The same tendency is reflected in the global reactivity descriptors. rA^O^G-DNA is characterised by the highest chemical hardness, whereas ^O^GA-DNA shows the highest softness and thus the greatest predicted electronic reactivity. All four investigated ds-oligonucleotides containing CDL systems remain strong electrophiles, but their reactivity is not equivalent.

The above is well-supported by the Fukui-function analysis, concerning atom-resolved reactivity (Table 2). The positions with the largest positive dual-descriptor (Δ*f_k_*) values differ depending on the arrangement of rAdo versus ^O^G, whereas the principal nucleophilic sites are predominantly associated with the atoms of the ^O^G moiety, including N^2^, O^8^ and, depending on the system, O^6^ and C^5^. Thus, relocation of the ^O^G lesion with respect to rAdo changes not only the energy required for electron addition or removal, but also the local susceptibility to subsequent nucleophilic, electrophilic and radical action. This observation may be particularly relevant for ^O^G because one-electron oxidation can initiate further transformation toward secondary lesions. Consequently, the chemical fate of an ^O^G lesion occurring near an embedded ribonucleotide may depend on its exact position within the clustered site rather than simply on the presence of the two non-canonical DNA nucleosides.

The presented findings should also be considered in the context of previous theoretical investigations [25]. Isolated, tandem and clustered oxidative DNA damage have already been found to induce electronic changes for systems containing ^O^G and other lesions. In particular, theoretical comparison of single ^O^G, tandem 5′,8-cyclo-2′-deoxyadenosine and their clustered combination showed that CDLs substantially modify the electronic properties of the double helix [25]. Subsequent analysis of isolated and clustered ^O^G lesions further demonstrated that the position of ^O^G within a short sequence determines radical cation localisation and charge-transfer nature [24].

Conversely, recent calculations demonstrated that even a single embedded ribonucleotide can modify the structural and electronic properties of otherwise canonical ds-DNA, including its ionisation potential, electron affinity and charge-transfer characteristics [27]. Further analysis showed that cleavage or rearrangement of the phosphodiester linkage associated with an embedded ribonucleotide can also influence charge-transfer properties, and alter proton-coupled electron transfer in selected structures [28]. While these studies established the capacity of an individual rN to modulate DNA electronics, they did not address its behaviour as a single subunit of a clustered oxidative damage site.

In conclusion, the present study therefore connects two previously investigated but largely separate problems: the influence of ribonucleotide incorporation on DNA electronics and the influence of ^O^G-containing clustered lesions on charge transport and reactivity [24,27]. Its novelty lies in the systematic demonstration that an embedded, chemically unmodified riboadenosine can act as an integral component of an ^O^G-containing clustered lesion, and that the resulting electronic phenotype depends strongly on the precise mutual organisation of rAdo and ^O^G. Structures where rAdo is directly paired with ^O^G, or where an ^O^GC pair is positioned either side of rAdo, or where rAdo is positioned adjacent to an ^O^G_4_A_2_ mispair, are closely related chemically. However, they have been found to differ with regard to geometry, stacking interactions, charge and spin localisation, ionisation potential, electron affinity and atom-resolved reactivity. In selected topologies, these changes also include proton-coupled charge redistribution. This data provides a molecular context for the biochemical observations reported for the rA^O^G mispair [40], which also established that the co-occurrence of an oxidised base and ribonucleotide can alter the normal functional separation between BER and RER.

## 4. Materials and Methods

The double-stranded oligonucleotides were subjected to ground-state geometry optimisation, as previously described [27].

The following notation for the investigated ds-oligonucleotides (ds-oligos) was applied, with the nucleoside sequences given in square brackets: GrA^O^G-DNA d[A_1_G_2_rA_3_^O^G_4_A_5_]*d[T_5_C_4_T_3_C_2_T_1_], ^O^GrAG-DNA d[A_1_^O^G_2_rA_3_G_4_A_5_]*d[T_5_C_4_T_3_C_2_T_1_], rA^O^G-DNA d[A_1_G_2_rA_3_G_4_A_5_]*d[T_5_C_4_^O^G_3_C_2_T_1_] and ^O^GA-DNA d[A_1_G_2_rA_3_^O^G_4_A_5_]*d[T_5_C_4_T_3_A_2_T_1_]. The 3D geometries of the ds-DNAs with clustered damage sites are presented in Figure 2.

### 4.1. Geometry Optimisation of the Ds-Oligonucleotides

All geometry optimisation was performed using the global hybrid functional (Minnesota M06-2X with 54% of Hartree-Fock exchange).

The benchmarks performed for the M06-2X functional disclose mean unsigned errors and balanced unsigned mean errors at the level of 1–3 kcal·mol^−1^, indicating high accuracy for the main group [73]. The solvent environment was described using Tomasi’s Conductor-like Polarizable Continuum Model (C-PCM), with a water dielectric constant ε = 78.4 [74]. The short double-stranded DNA systems were divided into two levels of calculation using the ONIOM (**O**ur own **N**-layered **I**ntegrated Molecular **O**rbital and Molecular **M**echanics) method, i.e., high level, M06-2X/D95**-HL (all the nucleobases), and low level, M06-2X/sto-3G-LL (sugar–phosphate backbones) [75,76]. Please see Figure S1. (Due to the high time consumption of theoretical experiments, the D95** Dunning–Huzinaga full double-zeta basis set was used for base-pair ladder geometry optimisation in the aqueous phase [77,78].) The above strategy allowed for significantly reduced the computational cost to a reasonable level [24].

Following this, the phosphate groups were removed from the ground-state DNA double-helix structure, leaving a suitable nucleoside-pair framework. Hydrogen atoms were added as necessary to saturate the atoms. The spatial positions of the additional hydrogen atoms were optimised at the same level of theory (M06-2X/D95**) in the aqueous phase.

### 4.2. Energy Calculations of Ds-Oligonucleotides

All energy calculations were conducted in the aqueous phase using density functional theory (DFT) with the M06-2X functional and an augmented polarised valence double-ζ basis set: 6-31++G** in the aqueous phase [73,79]. The calculations were performed using Tomasi’s Conductor-like Polarizable Continuum Model [80]. This was applied to all discussed ds-oligonucleotides, including complete oligonucleotides, nucleoside-pair skeletons without phosphate groups, and nucleoside pairs and their suitable dimers.

### 4.3. Calculation of the Electronic Properties of Ds-Oligonucleotides

The molecular electronic properties presented in this article, i.e., vertical (VIP) and adiabatic (AIP) ionisation potentials and vertical (VEA) and adiabatic (AEA) electron affinities, were obtained as described previously [79]. The solvent–solute interaction was analysed in two scenarios, *viz.*, nonequilibrated (**NE**) and equilibrated (**EQ**), using the Conductor-like Polarizable Continuum Model [81].

The following energy notation was used [82]: the *E*_Geometry_^Charge^ of the molecule (neutral form) is described as *E*_0_^0^, the vertical cation *E*_0_^+^, vertical anion *E*_0_^−^, the adiabatic cation *E*_+_^+^, and the adiabatic anion as *E*_−_^−^.

The differences between the aforementioned energy states correspond to the following electronic properties: ^NE^VIP = ^(NE)^*E*_0_^+^ − *E*_0_^0^ (vertical ionisation potential in the NE state); ^EQ^VIP = ^(EQ)^*E*_0_^+^ − *E*_0_^0^ (vertical ionisation potential in the EQ state); AIP = *E_+_*^+^ − *E*_0_^0^ (adiabatic ionisation potential); ^NE^VEA = *E*_0_^0^ − ^(NE)^*E*_0_^−^ (vertical electron affinity in the NE state); ^EQ^VEA = *E*_0_^0^ − ^(EQ)^*E*_0_^−^ (vertical electron affinity in the EQ state); AEA = *E*_0_^0^ − *E_−_* (adiabatic electron affinity).

### 4.4. Software

The initial ds-DNA structures were constructed using BioVia Discovery Studio v20.1.0.19295 software [83]. All theoretical calculations (geometries and energies) were performed with the Gaussian G16 (version C.01) program suite [84]. The DNA standard reference frame analyses were performed using W3DNA (version 2.0) software via the web 3DNA online interface [85].

## 5. Conclusions

The present results of theoretical studies suggest that this particular mispair may only be a single member of a broader group of topology-dependent rN/^O^G-containing sites. Different mutual arrangements of rAdo and ^O^G generate distinct structural and electronic landscapes and therefore may, in principle, be recognised or processed differently by RNase H2, OGG1 or MutYH. However, further direct biochemical analysis of the four tested arrangements is needed to confirm this. Nevertheless, the present results demonstrate that the biological potential of an rAdo and ^O^G clustered lesion cannot be defined solely by its chemical composition. Moreover, it appears that the spatial architecture of the discussed damage site plays an important role in determining its electronic properties and, potentially, its recognition and removal by the proteins of DNA repair machinery.

## Figures and Tables

**Table 1:** (a) The structural parameters of heterocycle overlap (*OL^BP^*) in the nucleoside-pair dimers, in [Å^2^], and the distance between them denoted as *Rise*, in [Å]. The geometries of the neutral ds-oligos were calculated at the M06-2X/D95**:M06-2X/sto-3G level of theory in the aqueous phase using the ONIOM strategy. Differences in geometry among the neutral, anionic, and cationic forms of the investigated ds-DNAs are presented as the root mean square deviation (RMSD) of the atomic positions of the complete ds-oligonucleotide structures; (b) stacking interaction energies, *E_ST_*, given in kcal·mol^−1^, between nucleoside pairs in the dimers of the investigated ds-oligonucleotides in their adiabatic neutral forms, optimised at the M06-2X/6-31++G** level of theory in the aqueous phase; (c) the phase, amplitude and puckering of the ribose moiety of riboadenosine (rA_3_), the dihedral angle C4-N9-C1′-O4′ (χ) around the glycosidic bond and the dihedral angle N9-C1′-C2′-O2′(ε*) were used to indicate the 2′-OH group position. Neu—neutral, Cat—Cation, Ani—Anion.

Comparison	^O^GrAG-DNA (G_2_ = ^O^G)			GrA^O^G-DNA (G_4_ = ^O^G)			rA^O^G-DNA (T_3_ = ^O^G)			^O^GA-DNA (G_4_ = ^O^G T_2_ = A)		
	Root Mean Square Deviation [Å]											
Neutral vs. Anion	0.85			0.40			0.40			1.32		
Neutral vs. Cation	0.50			0.64			0.38			0.89		
Anion vs. Cation	0.95			0.57			0.49			0.81		
Base-Pair Dimer	^O^GrAG-DNA (G_2_ = ^O^G)			GrA^O^G-DNA (G_4_ = ^O^G)			rA^O^G-DNA (T_3_ = ^O^G)			^O^GA-DNA (G_4_ = ^O^G T_2_ = A)		
	*E_ST_*	*OL^BP^*	*Rise*	*E_ST_*	*OL^BP^*	*Rise*	*E_ST_*	*OL^BP^*	*Rise*	*E_ST_*	*OL^BP^*	*Rise*
A_1_T_5_|G_2_C_4_	27.82	6.95	2.74	26.55	3.78	2.90	26.98	8.88	2.98	26.83	7.37	2.98
G_2_C_4_|rA_3_T_3_	28.64	11.25	2.97	22.74	7.63	2.92	22.41	6.35	3.06	27.29	1.93	3.17
rA_3_T_3_|G_4_C_2_	21.76	7.28	3.16	34.58	8.27	2.89	28.75	9.89	2.94	38.34	12.42	3.05
G_4_C_2_|A_5_T_1_	27.97	8.07	3.14	27.85	7.92	3.11	26.78	9.69	3.09	25.82	3.91	2.46
	^O^GrAG-DNA (G_2_ = ^O^G)			GrA^O^G-DNA (G_4_ = ^O^G)			rA^O^G-DNA (T_3_ = ^O^G)			^O^GA-DNA (G_4_ = ^O^G T_2_ = A)		
ryboAdenosien (**rA_3_**)	**Neu**	**Cat**	**Ani**	**Neu**	**Cat**	**Ani**	**Neu**	**Cat**	**Ani**	**Neu**	**Cat**	**Ani**
Phase [^O^]	146.1	145.3	142.2	169.9	169.1	161.1	142.9	153.6	144.8	207.2	181.2	158.3
Amplitude [^O^]	45.5	49.1	48.8	37.5	41.6	43.1	48.2	45.7	48.7	35.8	38.53	41.3
Puckering	^2^ **T**_1_	^2^ **T**_1_	^2^ **T**_1_	^2^ **T**_3_	^2^ **T**_3_	^2^ **T**_3_	^2^ **T**_1_	^2^ **T**_1_	^2^ **T**_1_	^1^ **T**_3_	^2^ **T**_3_	^2^ **T**_1_
C4-N9-C1′-O4′ (χ) [^O^]	−99.1	−97.1	−79.4	−90.2	−85.1	−92.1	−105.3	−103.0	−102.8	−98.4	−90.6	−98.6
N9-C1′-C2′-O2′(ε*) [^O^]	−72.5	−72.7	−115.9	−87.9	−84.9	−83.0	−66.9	−73.0	−69.0	−106.2	−84.6	−76.0

**Table 2:** Calculated Fukui reactivity index of short ds-DNA containing riboadenosine and 7,8-dihydro-8-oxo-2′-deoxyguanosine in different distributions, as shown in Figure 2. The charge distributions used to estimate the Fukui functions were calculated at the M06-2X/6-31++G** level of theory in the aqueous phase, using charge model 5 based on Hirshfeld population methodology. *f_k_^+^* = Q_k_(N) − Q_k_(N + 1); *f_k_^−^* = Q_k_(N − 1) − Q_k_(N); *f_k_*^0^ = ½[Q_k_(N − 1) − Q_k_(N + 1)]; Δ*f_k_* = *f_k_^+^* − *f_k_^−^* = 2Q_k_(N) − Q_k_(N + 1) − Q_k_(N − 1); N—number of electrons in the system; Q_k_—atom charge (N + 1 anion, N − 1 cation). Nucleo.—nucleoside in ds-DNA structure. The raw data are given in Table S2. CM^5^ indicates the methyl group in thymidine structure.

Nucleo./Atom	*f_k_^+^*	*f_k_^−^*	*f_k_* ^0^	Δ*f_k_*	Nucleo./Atom	*f_k_^+^*	*f_k_^−^*	*f_k_* ^0^	Δ*f_k_*	Nucleo. Structure
	GrA^O^G-DNA					^O^GrAG-DNA				
**C_2_**/N^4^	0.08		0.05	0.07	**C_2_**/N^4^	0.07			0.13	* 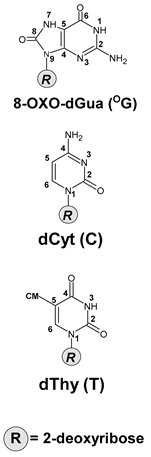 *
**C_2_**/C^5^	0.06			0.06	**C_2_/**C^4^	0.05			0.11	
**C_2_**/C^6^	0.12		0.06	0.11	**C_2_**/C^6^	0.11		0.05	0.16	
					**C_4/_**C^6^	0.07			0.06	
**^O^G_4_**/N^2^		0.11	0.06	−0.10	**^O^G_2_**/N^2^		0.11	0.06	−0.13	
**^O^G_4_**/O^6^		0.09	0.05	−0.08	**^O^G_2_/**O^6^		0.08		−0.09	
**^O^G_4_**/C^5^		0.10	0.05	−0.09	**^O^G_2_/**C^5^		0.10	0.05	−0.10	
**^O^G_4_/**N^7^		0.09		−0.08	**^O^G_2_/**N^7^		0.10	0.05	−0.11	
**^O^G_4_/**O^8^		0.08		−0.08	**^O^G_2_**/O^8^		0.10	0.05	−0.12	
**^O^G_4_**/N^3^		0.07		−0.06	**^O^G_2_**/N^3^		0.07		−0.07	
	**rA^O^G-DNA**					**^O^** **GA-DNA**				
**C_2_**/N^4^	0.07			0.15	**T_3_**/O^4^	0.07			0.07	
**C_2_/**C^4^	0.05			0.11	**T_3_**/C^4^	0.06			0.06	
**C_2_**/C^6^	0.12		0.06	0.14	**T_3_**/C^6^	0.11		0.06	0.11	
**C_2_**/C^5^	0.07			0.06	**T_3_**/CM^5^	0.05			0.05	
**^O^G_3_/**N^2^		0.11	0.06	−0.09	**C_4_**/C^6^	0.08			0.08	
**^O^G_3_**/O^6^		0.09	0.05	−0.09	**^O^G_4_**/N^2^		0.10	0.05	−0.09	
**^O^G_3_/**C^5^		0.09		−0.09	**^O^G_4_**/O^6^		0.08		−0.08	
**^O^G_3_/**N^7^		0.08		−0.08	**^O^G_4_**/C^5^		0.08		−0.08	
**^O^G_3_**/O^8^		0.10	0.06	−0.09	**^O^G_4_**/N^7^		0.07		−0.07	
					**^O^G_4_**/O^8^		0.12	0.06	−0.12	

**Table 3:** The reactivity descriptors, in eV (except softness in eV^−1^), of rA^O^G-DNA, ^O^GA-DNA, ^O^GrAG-DNA and GrA^O^G-DNA calculated at the M06-2X/6-31++G** level of theory in the aqueous phase. The valence molecular orbital energies have been used according to Koopmans’ theorem (Koopmans’ Th.). The energy differences between vertical ionisation potential and vertical electron affinity were calculated in nonequilibrated (NE)/equilibrated (EQ) solvent–solute interactions. The HOMO and LUMO energies are given in Hartree.

	Molecular Orbitals		Koopmans’ Theorem				^NE^Vertical				^EQ^Vertical			
*ds-oligo*	HOMO	LUMO	*η*	*S*	*μ*	*ω*	*η*	*S*	*μ*	*ω*	*η*	*S*	*μ*	*ω*
**rA^O^G-DNA**	−0.2576	−0.0239	3.18	0.31	−3.83	2.31	3.11	0.32	−3.65	2.15	2.37	0.42	−3.74	2.95
**^O^** **GA-DNA**	−0.2546	−0.0273	3.09	0.32	−3.84	2.38	2.89	0.35	−3.82	2.53	2.27	0.44	−3.78	3.15
**^O^** **GrAG-DNA**	−0.2518	−0.0251	3.08	0.32	−3.77	2.30	2.94	0.34	−3.66	2.28	2.30	0.43	−3.65	2.89
**GrA^O^G-DNA**	−0.2558	−0.0248	3.14	0.32	−3.82	2.32	2.99	0.33	−3.78	2.39	2.35	0.43	−3.77	3.01

**Table 4:** The electronic parameters in **[**eV**]** of the double-stranded oligonucleotides (complete double helix) denoted as rA^O^G-DNA, ^O^GA-DNA, ^O^GrAG-DNA, GrA^O^G-DNA and extracted nucleoside pairs calculated at the M06-2X/6-31++G** level of theory in the aqueous phase. VIP—vertical ionisation potential, AIP—adiabatic ionisation potential, VEA—vertical electron affinity, AEA—adiabatic electron affinity, EQ indicates the equilibrated solvent–solute interaction mode. N. Pair—nucleoside pair. The raw data are given in Tables S3 and S4.

N. Pair	rA^O^G-DNA				N. Pair	^O^GA-DNA				Double-Stranded Oligonucleotide					
	^EQ^VIP	AIP	^EQ^VEA	AEA		^EQ^VIP	AIP	^EQ^VEA	AEA	^NE^VIP	^EQ^VIP	AIP	^NE^VEA	^EQ^VEA	AEA
**A_1_T_5_**	6.59	6.60	1.35	1.36	**A_1_T_5_**	6.60	6.43	1.61	1.64	**rA^O^G-DNA**					
**G_2_C_4_**	6.16	6.18	1.47	1.46	**G_2_C_4_**	6.17	5.99	1.63	2.33	6.76	6.10	5.21	0.55	1.37	2.01
**rA_3_^O^G_3_**	5.99	5.15	1.33	1.45	**rA_3_T_3_**	6.68	6.46	1.65	1.85	**^O^** **GA-DNA**					
**G_4_C_2_**	6.33	6.26	1.60	2.01	**^O^** **G_4_A_2_**	5.97	4.99	1.28	1.47	6.71	6.05	3.39	0.94	1.51	4.07
**A_5_T_1_**	6.69	6.69	1.39	1.36	**A_5_T_1_**	6.69	6.55	1.54	1.59	**^O^** **GrAG-DNA**					
	**^O^** **GrAG-DNA**					**GrA^O^G-DNA**				6.60	6.00	5.95	0.73	1.35	1.34
**A_1_T_5_**	6.61	6.42	1.50	1.38	**A_1_T_5_**	6.61	5.72	1.50	1.51	**GrA^O^G-DNA**					
**^O^** **G_2_C_4_**	5.96	5.61	1.55	1.28	**G_2_C_4_**	6.15	5.81	1.55	1.59	6.78	6.12	6.11	0.79	1.41	1.46
**rA_3_G_3_**	6.69	6.87	1.48	1.12	**rA_3_G_3_**	6.67	6.63	1.48	1.45						
**G_4_C_2_**	6.25	6.15	1.57	2.07	**^O^** **G_4_C_2_**	5.98	5.24	1.58	1.96						
**A_5_T_1_**	6.77	6.74	1.38	1.31	**A_5_T_1_**	6.73	5.79	1.38	1.33						

## Data Availability

The original contributions presented in this study are included in the article/Supplementary Material. Further inquiries can be directed to the corresponding author.

## Supplementary Materials

The following supporting information can be downloaded at: https://www.mdpi.com/article/10.3390/molecules31203673/s1, Table S1. Hirshfeld spin (S-H) and the charge model 5 based on the Hirshfeld methodology (Q-CM5), distribution of nucleosides extracted from complete ds-DNAs, calculated at the M06-2X/6-31++G** level of theory in the aqueous phase. Vertical cation (VC^NE^) (NE—nonequilibrated), vertical cation (VC^EQ^) (EQ—equilibrated), adiabatic cation (AC) and vertical anion (VA^NE^), vertical anion (VA^EQ^), adiabatic anion (AA); Table S2. Atom-condensed Fukui reactivity indices. For net atomic charges Qk evaluated at the fixed neutral geometry, the charge-based convention used throughout the manuscript and Supplementary Materials is the following: f_k_^+^ = Q_k_(N) − Q_k_(N + 1); f_k_^−^ = Q_k_(N − 1) − Q_k_(N); f_k_^0^ = ½[Q_k_(N − 1) − Q_k_(N + 1)]; Δf_k_ = f_k_^+^ − f_k_^−^ = 2Q_k_(N) − Q_k_(N + 1) − Q_k_(N − 1). Here, N is the electron number of the neutral system; N + 1 denotes the anion and N−1 the cation. With this charge convention, Δfk > 0 denotes a predominantly electrophilic atomic site (susceptible to nucleophilic attack), whereas Δfk < 0 denotes a predominantly nucleophilic atomic site (susceptible to electrophilic attack). The Q-CM5 charge distribution was calculated at the M06-2X/6-31++G** level in aqueous continuum solvent using charge model 5 based on Hirshfeld population analysis; Table S3. The energies (in Hartree) of neutral, vertical cation (VC^NE^) (NE—nonequilibrated), vertical cation (VC^EQ^) (EQ—equilibrated), vertical anion (VA^NE^), vertical anion (VA^EQ^), adiabatic cation (AC), adiabatic anion (AA) of complete DNA double helix calculated at the M06-2x/6-31++G** level of theory in the aqueous phase; Table S4. The energies (in Hartree) of neutral, vertical cation (VC^EQ^) (EQ—equilibrated), vertical anion (VA^EQ^), adiabatic cation (AC), adiabatic anion (AA) of nucleoside pairs extracted from complete DNA double helix calculated at the M06-2x/6-31++G** level of theory in the aqueous phase; Table S5. Hirshfeld spin (S-H) and the charge model 5 based on the Hirshfeld methodology (Q-CM5), distribution of all have atoms presence in complete ds-DNAs, calculated at the M06-2X/6-31++G** level of theory in the aqueous phase. Vertical cation (VC^NE^) (NE—nonequilibrated), vertical cation (VC^EQ^) (EQ—equilibrated), adiabatic cation (AC) and vertical anion (VA^NE^), vertical anion (VA^EQ^), adiabatic anion (AA); Table S6. Local riboadenosine sugar-pucker and conformational descriptors for the neutral, adiabatic cation, and adiabatic anion structures. ν0–ν4 are the five endocyclic furanose torsions angles; glicosidic torsion angle χ = C4–N9–C1′–O4′; dihedral angle indicated 2′-OH position denoted as ε* = N9–C1′–C2′–O2′; pseudorotation phase and amplitude are reported in degrees[^O^]; Figure S1. Graphical representation of investigated d[AGrAGA]*d[TCTCT] duplex (example) was divided into two levels of calculation using the ONIOM (our own N-layered Integrated Molecular Orbital and Molecular Mechanics) method, i.e., high level, M06-2X/D95**-HL (all the base-pair ladder), and low level, M06-2X/sto-3G-LL (sugar–phosphate backbones) in the aqueous phase. The zip file (PDB Structures.zip) of optimised PDB structure of discussed structures (neutral, cation, anion) ds-oligonucleotides.
