# Supplementary material for: The Impact of rAdo and OXOdGuo on the Electronic Properties of Clustered DNA Lesions: A Theoretical Approach
Source: Molecules. 2026 Oct 14;31(20):3673. doi: 10.3390/molecules31203673 (PMC13707549; doi:10.3390/molecules31203673)

Supplementary Materials

The impact of rAdo and ^{OXO}dGuo on the electronic properties of clustered DNA lesions. A theoretical approach.

Boleslaw Karwowski

Nucleic Acids Damage Laboratory of Food Science Department, Faculty of Pharmacy, Medical University of Lodz, ul. Muszyńskiego 1, 90-151 Lodz, Poland; e-mail@e-mail.com

Table S1. Hirshfeld spin (S-H) and the charge model 5 based on the Hirshfeld methodology (Q-CM5), distribution of nucleosides extracted from complete ds-DNAs, calculated at the M06-2x/6-31++G** level of theory in the aqueous phase. Vertical Cation (VC^{NC}) (NE-non-equilibrated), Vertical Cation (VC^{EQ}) (EQ-equilibrated), Adiabatic Cation (AC) and Vertical Anion (VA^{NE}), Vertical Anion (VA^{EQ}), Adiabatic Anion (AA)

^{O}GA-DNA Nucleoside	Neutral	^{NE}Vertical Anion		^{EQ}Vertical Anion		Adiabatic Anion		^{NE}Vertical Cation		^{EQ}Vertical Cation		Adiabatic Cation	
	Q-CM5	S-H	Q-CM5	S-H	Q-CM5	S-H	Q-CM5	S-H	Q-CM5	S-H	Q-CM5	S-H	Q-CM5
T_1	-0.156343	0.000115	-0.165234	0.000668	-0.162132	0.000005	-0.125555	0.000028	-0.143937	7E-06	-0.14779	0.000012	-0.115531
P	0.133424	0.000025	0.130278	0.000015	0.130956	-0.000001	0.134357	0	0.137999	-0.000002	0.133686	-0.000002	0.137737
A_2	-0.076472	0.006835	-0.091501	0.018803	-0.10703	0.000303	-0.081673	0.000897	-0.05109	0.00019	-0.039507	0.002292	0.499986
P	0.113563	0.000802	0.105738	0.00065	0.106348	0.000006	0.121304	0	0.119179	-0.000001	0.114164	0.000003	0.122375
T_3	-0.166948	0.214346	-0.344536	0.546332	-0.628934	0.027662	-0.214971	0.000078	-0.152566	0.000071	-0.1553	0.000147	-0.165976
P	0.135544	0.006464	0.116662	0.000436	0.125676	0.000359	0.121695	0	0.142315	0	0.136146	-1E-06	0.133751
C_4	0.040421	0.575027	-0.426839	0.326026	-0.252682	0.931103	-0.687451	0.000722	0.049993	0.000065	0.04733	0.00001	0.048441
P	0.11823	0.003801	0.100813	0.000238	0.111455	0.000296	0.11476	0	0.125491	0	0.118652	-0.000002	0.130324
T_5	-0.117361	0.145129	-0.264087	0.087502	-0.207963	0.0238	-0.149932	0.001189	-0.105935	0.000052	-0.114111	0.000008	-0.102931
A_1	-0.078248	0.006055	-0.104732	0.001867	-0.089265	0.005601	-0.140849	0.00964	-0.051461	0.000168	-0.074719	0.000009	-0.098913
P	0.115483	0.000002	0.111451	0	0.113918	0.000001	0.113253	0	0.120732	-0.000003	0.115437	0.000003	0.11441
G_2	-0.181881	0.026444	-0.229635	0.007505	-0.204393	0.008448	-0.287238	0.187122	-0.02708	0.009559	-0.165984	0.000796	-0.199034
P	0.192972	0.000015	0.186857	0	0.19067	0	0.14617	0.000114	0.200687	0.000135	0.197661	0.000021	0.161943
rA_3	-0.175576	0.013637	-0.198227	0.00946	-0.211963	0.002362	-0.121348	0.14709	-0.048316	0.079267	-0.08278	0.02646	-0.009039
P	0.0041	0.000004	-0.001806	0.000001	0.001638	0	0.049387	0.000073	0.017819	0.000076	0.011482	0	0.069158
$^{O}G_4$	-0.038982	0.000849	-0.047273	0.000299	-0.04647	0.000047	-0.097377	0.616994	0.459097	0.869198	0.689546	0.948523	0.09979
P	0.107605	0.000017	0.102978	0.000001	0.105813	-0.000001	0.104796	0.000014	0.121623	0.000003	0.114689	-0.000008	0.115189
A_5	0.030736	0.00044	0.019354	0.000198	0.024631	0.000009	0.000617	0.036038	0.085711	0.041216	0.101661	0.021725	0.058196

rA^{O}G-DNA Nucleoside	Table S1 continued												
	Neutral	^{NE}Vertical Anion		^{EQ}Vertical Anion		Adiabatic Anion		^{NE}Vertical Cation		^{EQ}Vertical Cation		Adiabatic Cation	
	Q-CM5	S-H	Q-CM5	S-H	Q-CM5	S-H	Q-CM5	S-H	Q-CM5	S-H	Q-CM5	S-H	Q-CM5
T_1	-0.190679	-0.147012	0.044815	-0.207875	0.372878	-0.503336	0.031351	-0.222546	0.002277	-0.12835	0.001202	-0.134911	0.000522
P	0.148689	0.129029	0.049363	0.079537	0.001022	0.118649	0.000216	0.1167	0.000017	0.134548	0.000008	0.130076	0.000008
C_2	-0.072043	-0.062812	0.355868	-0.361682	0.550044	-0.518405	0.907976	-0.740472	0.042009	-0.019965	0.030611	-0.015226	0.020083
P	0.12477	0.114946	0.107817	0.010613	0.000272	0.106179	0.000187	0.10705	0.000008	0.12814	-0.000002	0.120231	0
$^{O}G_3$	-0.09288	-0.08192	0.187545	-0.259394	0.050325	-0.147508	0.046736	-0.163165	0.893149	0.652401	0.948783	0.725727	0.952477
P	0.164876	0.142299	0.096698	0.053125	0.000027	0.139413	0.000012	0.135633	0.000021	0.156289	-0.000002	0.14906	-4E-06
C_4	0.03881	0.011224	0.062904	-0.052159	0.007083	-0.000774	0.000381	0.00176	0.020411	0.036467	0.016512	0.049068	0.020875
P	0.218486	0.180694	0.018919	0.16131	0.000017	0.179693	0	0.178821	0.000007	0.188615	0.000004	0.182326	0.000015
T_5	-0.165342	-0.133002	0.012389	-0.155401	0.000145	-0.134756	0.000001	-0.107721	0.00048	-0.11764	0.000174	-0.124705	0.000234
A_1	-0.138322	-0.10975	0.001514	-0.11688	0.00002	-0.110544	0.000002	-0.114682	0.001645	-0.099303	0.000014	-0.106237	0.000002
P	0.125097	0.115012	0.000043	0.113223	0.000001	0.114462	0	0.115776	0	0.118121	0.000003	0.114103	0
G_2	-0.21038	-0.188055	0.004616	-0.200049	0.000211	-0.190579	0.000105	-0.198499	0.018796	-0.159648	0.000723	-0.175727	0.000344
P	0.21061	0.165418	0.000064	0.163845	0.000004	0.1649	0.000001	0.165493	0	0.169261	-0.000003	0.165371	-0.000001
rA_3	-0.187079	-0.149088	0.012781	-0.170616	0.004023	-0.162837	0.00273	-0.140091	0.001485	-0.116714	-0.000195	-0.114302	0.003004
P	0.246008	0.17321	0.000243	0.168915	0.00001	0.171517	0.000004	0.165737	0.000012	0.179069	0	0.172971	0.000001
G_4	-0.4166	-0.310004	0.019233	-0.337662	0.008011	-0.343175	0.007087	-0.398127	0.019302	-0.29142	0.002062	-0.293953	0.002372
P	0.124283	0.112437	0.0004	0.107384	0	0.111271	0	0.107919	0	0.118086	0	0.111731	0
A_5	0.071088	0.03677	0.02486	0.003072	0.005914	0.005219	0.003222	-0.009885	0.00037	0.051447	0.000106	0.043805	0.000063

GrA^{O}G-DNA Nucleoside	Table S1 continued												
	Neutral	^{NE}Vertical Anion		^{EQ}Vertical Anion		Adiabatic Anion		^{NE}Vertical Cation		^{EQ}Vertical Cation		Adiabatic Cation	
	Q-CM5	S-H	Q-CM5	S-H	Q-CM5	S-H	Q-CM5	S-H	Q-CM5	S-H	Q-CM5	S-H	Q-CM5
T_1	-0.176737	-0.134361	0.059616	-0.209867	0.268856	-0.395505	0.035209	-0.192443	0.000019	-0.125062	-0.000025	-0.125377	0.000106
P	0.152469	0.132509	0.021429	0.103265	0.001027	0.124210	0.000383	0.118506	0.000000	0.136028	-0.000003	0.132117	-0.000002
C_2	0.069206	0.046375	0.494990	-0.366943	0.536105	-0.423246	0.930602	-0.680361	0.001002	0.063989	0.001301	0.080850	0.000312
P	0.133934	0.122370	0.033850	0.080760	0.000566	0.114564	0.000233	0.111204	0.000000	0.127347	0.000000	0.122405	-0.000001
T_3	-0.221715	-0.190507	0.160172	-0.330210	0.149700	-0.331122	0.019606	-0.235777	0.002082	-0.178124	0.002799	-0.173481	0.002734
P	0.151351	0.132895	0.025163	0.102603	0.000178	0.129773	0.000000	0.131592	0.000000	0.139290	-0.000002	0.132975	-0.000002
C_4	0.049918	0.030517	0.104624	-0.060214	0.021949	0.006627	0.000026	0.036981	0.000510	0.047939	0.000099	0.036743	0.000053
P	0.130962	0.120030	0.007719	0.106782	0.000025	0.118626	0.000000	0.130039	0.000000	0.126333	0.000000	0.119380	-0.000001
T_5	-0.070499	-0.071583	0.028393	-0.108068	0.004789	-0.078480	-0.000001	-0.105932	0.001550	-0.057567	0.000005	-0.068907	0.000004
A_1	-0.141012	-0.112006	0.001704	-0.120201	0.000105	-0.113200	0.000007	-0.104736	0.030993	-0.059647	0.000090	-0.108478	0.000028
P	0.125768	0.115005	0.000030	0.113076	0.000000	0.114445	0.000000	0.114631	0.000003	0.122589	0.000004	0.114106	0.000001
G_2	-0.268152	-0.234198	0.001434	-0.243872	0.000446	-0.237912	0.000059	-0.212630	0.396105	0.091187	0.004089	-0.220230	0.001485
P	0.156610	0.136378	0.000007	0.134236	0.000000	0.135811	0.000000	0.149643	0.000008	0.146119	-0.000004	0.137347	-0.000002
rA_3	-0.129204	-0.115753	0.002826	-0.130592	0.001131	-0.127697	0.001464	-0.162874	0.163176	0.029583	0.079535	-0.012796	0.061949
P	0.044435	0.036951	0.000021	0.032849	0.000001	0.036534	-0.000001	0.035594	0.000026	0.048095	0.000037	0.041822	0.000041
$^{O}G_4$	-0.143947	-0.119024	0.025215	-0.158100	0.007862	-0.148617	0.007963	-0.240691	0.388777	0.192660	0.881820	0.617012	0.908086
P	0.075768	0.074284	0.000477	0.068425	0.000003	0.073255	0.000000	0.100908	0.000005	0.084255	0.000006	0.079863	-0.000012
A_5	0.060589	0.029873	0.032297	-0.014140	0.007282	0.001679	0.004421	0.006259	0.015747	0.064746	0.030250	0.094401	0.025225

^{O}GrAG-DNA Nucleoside	Table S1 continued												
	Neutral	^{NE}Vertical Anion		^{EQ}Vertical Anion		Adiabatic Anion		^{NE}Vertical Cation		^{EQ}Vertical Cation		Adiabatic Cation	
	Q-CM5	S-H	Q-CM5	S-H	Q-CM5	S-H	Q-CM5	S-H	Q-CM5	S-H	Q-CM5	S-H	Q-CM5
T_1	-0.188880	-0.143889	0.017217	-0.174146	0.304478	-0.436849	0.032170	-0.188545	0.000006	-0.139667	0.000002	-0.143386	0.000000
P	0.150271	0.130497	0.012994	0.114693	0.001083	0.122442	0.000293	0.117251	0.000000	0.132823	-0.000002	0.130801	0.000000
C_2	0.060285	0.037911	0.152863	-0.096571	0.500282	-0.397586	0.924731	-0.655211	0.000359	0.042015	0.000095	0.040889	0.000014
P	0.135655	0.123174	0.054238	0.069487	0.000549	0.116115	0.000187	0.106888	0.000000	0.126692	0.000003	0.123827	-0.000001
T_3	-0.199747	-0.176651	0.191730	-0.345718	0.116028	-0.290590	0.021409	-0.191085	0.000589	-0.166714	0.000450	-0.167437	0.000470
P	0.151141	0.132856	0.057303	0.073020	0.000195	0.130427	-0.000001	0.135437	0.000000	0.138341	-0.000003	0.133810	-0.000002
C_4	0.000085	-0.007213	0.377267	-0.321604	0.055081	-0.057279	0.000096	-0.011469	0.001538	0.020415	0.000745	0.021996	0.000068
P	0.120315	0.110795	0.019816	0.084861	0.000038	0.109851	-0.000001	0.111370	0.000000	0.117498	0.000003	0.111908	-0.000001
T_5	-0.021359	-0.028437	0.060625	-0.105502	0.003929	-0.035898	0.000002	-0.021496	0.001936	-0.010945	0.001303	-0.015878	0.002313
A_1	-0.157231	-0.125451	0.007977	-0.144081	0.000557	-0.128242	0.000007	-0.132140	0.062007	-0.039228	0.041675	-0.051524	0.064771
P	0.076222	0.074649	0.000008	0.072089	0.000000	0.074791	0.000006	-0.040381	0.000063	0.088439	0.000031	0.081920	-0.000002
$^{O}G_2$	-0.123018	-0.124362	0.018264	-0.160379	0.001456	-0.131532	0.000143	-0.055133	0.843104	0.573919	0.899978	0.650653	0.899060
P	0.098882	0.086449	0.000026	0.083943	0.000000	0.086568	0.000002	0.084601	0.000006	0.099232	0.000001	0.092138	-0.000003
rA_3	-0.038422	-0.040056	0.018495	-0.069524	0.004892	-0.056766	0.010634	-0.116370	0.086852	0.037639	0.054567	0.028356	0.032665
P	0.203760	0.145995	0.000055	0.143040	0.000003	0.145814	0.000002	0.122202	0.000001	0.153405	-0.000004	0.148184	-0.000001
G_4	-0.429203	-0.320717	0.004714	-0.330366	0.005048	-0.347457	0.007393	-0.370448	0.003432	-0.313581	0.001126	-0.314380	0.000645
P	0.117856	0.108298	0.000053	0.104349	0.000000	0.107933	-0.000001	0.109103	0.000000	0.114000	0.000001	0.109035	0.000001
A_5	0.043426	0.016188	0.006353	0.002452	0.006399	-0.011727	0.002911	-0.004423	0.000100	0.025748	0.000017	0.019113	0.000005

Table S2. Atom-condensed Fukui reactivity indices. For net atomic charges Q_k evaluated at fixed neutral geometry, the charge-based convention used throughout the manuscript and Supplementary Materials is:

$f_k^+ = Q_k(N) - Q_k(N+1)$; $f_k^- = Q_k(N-1) - Q_k(N)$; $f_k^0 = ½[Q_k(N-1) - Q_k(N+1)]$; $\Delta f_k = f_k^+ - f_k^- = 2Q_k(N) - Q_k(N+1) - Q_k(N-1)$

Here, N is the electron number of the neutral system; N+1 denotes the anion and N−1 the cation. With this charge convention, $\Delta f_k > 0$ denotes a predominantly electrophilic atomic site (susceptible to nucleophilic attack), whereas $\Delta f_k < 0$ denotes a predominantly nucleophilic atomic site (susceptible to electrophilic attack).

The Q-CM5 charge distribution was calculated at the M06-2X/6-31++G** level in aqueous continuum solvent using Charge Model 5 based on Hirshfeld population analysis.

**GrA^{O}G-DNA**						**^{O}GrAG-DNA**					
**Atom number**	**Nucleotide**	f_k^+	f_k^-	f_k^0	Δf_k	**Atom number**	**Nucleotide**	f_k^+	f_k^-	f_k^0	Δf_k
O5'	DT_1	0.00	0.00	0.00	0.01	O5'	DT_1	0.01	0.00	0.00	0.00
O3'	DT_1	0.00	0.00	0.00	0.00	O3'	DT_1	0.00	0.00	0.00	0.00
C1'	DT_1	0.01	0.00	0.00	0.01	C1'	DT_1	0.01	0.00	0.00	0.00
C2'	DT_1	0.01	0.00	0.00	0.01	C2'	DT_1	0.01	0.00	0.00	0.00
C3'	DT_1	0.01	0.00	0.00	0.01	C3'	DT_1	0.01	0.00	0.00	0.00
C4'	DT_1	0.01	0.00	0.00	0.01	C4'	DT_1	0.01	0.00	0.00	0.00
C5'	DT_1	0.01	0.00	0.00	0.01	C5'	DT_1	0.01	0.00	0.00	0.00
O4'	DT_1	0.01	0.00	0.00	0.01	O4'	DT_1	0.01	0.00	0.00	0.00
C4	DT_1	0.03	0.00	0.02	0.03	C4	DT_1	0.03	0.00	0.02	0.00
N3	DT_1	0.01	0.00	0.01	0.01	N3	DT_1	0.01	0.00	0.01	0.00
C2	DT_1	0.00	0.00	0.00	0.00	C2	DT_1	0.00	0.00	0.00	0.00
O2	DT_1	0.02	0.00	0.01	0.02	O2	DT_1	0.02	0.00	0.01	0.00
N1	DT_1	0.00	0.00	0.00	0.00	N1	DT_1	0.01	0.00	0.00	0.00
C6	DT_1	0.05	0.00	0.03	0.05	C6	DT_1	0.06	0.00	0.03	0.00
C5	DT_1	0.02	0.00	0.01	0.02	C5	DT_1	0.03	0.00	0.01	0.00
O4	DT_1	0.04	0.00	0.02	0.05	O4	DT_1	0.05	0.00	0.02	0.01

CM5	DT1	0.03	0.00	0.02	0.03	CM5	DT1	0.04	0.00	0.02	0.01
		0.00	0.00	0.00	0.00						
P1	DC2	0.00	0.00	0.00	0.00	P1	DC2	0.00	0.00	0.00	0.00
PO2	DC2	0.00	0.00	0.00	0.01	PO2	DC2	0.00	0.00	0.00	0.00
PO1	DC2	0.00	0.00	0.00	0.00	PO1	DC2	0.00	0.00	0.00	0.00
O5'	DC2	0.00	0.00	0.00	0.00	O5'	DC2	0.00	0.00	0.00	0.00
O3'	DC2	0.00	0.00	0.00	0.00	O3'	DC2	0.00	0.00	0.00	0.00
C1'	DC2	0.02	0.00	0.01	0.02	C1'	DC2	0.02	0.00	0.01	0.02
C2'	DC2	0.02	0.00	0.01	0.02	C2'	DC2	0.01	0.00	0.01	0.02
C3'	DC2	0.01	0.00	0.00	0.01	C3'	DC2	0.01	0.00	0.00	0.01
C4'	DC2	0.01	0.00	0.00	0.01	C4'	DC2	0.01	0.00	0.00	0.01
C5'	DC2	0.01	0.00	0.00	0.01	C5'	DC2	0.01	0.00	0.00	0.01
O4'	DC2	0.00	0.00	0.00	0.00	O4'	DC2	0.00	0.00	0.00	0.00
N4	DC2	0.08	0.01	0.05	0.07	N4	DC2	0.07	0.00	0.04	0.13
C4	DC2	0.05	0.00	0.03	0.05	C4	DC2	0.05	0.00	0.02	0.11
N3	DC2	0.03	0.00	0.01	0.03	N3	DC2	0.03	0.00	0.01	0.04
C2	DC2	0.01	0.00	0.01	0.01	C2	DC2	0.01	0.00	0.01	0.02
O2	DC2	0.03	0.00	0.01	0.03	O2	DC2	0.03	0.00	0.01	0.05
N1	DC2	0.02	0.01	0.01	0.02	N1	DC2	0.02	0.00	0.01	0.03
C6	DC2	0.12	0.01	0.06	0.11	C6	DC2	0.11	0.00	0.05	0.16
C5	DC2	0.06	0.00	0.03	0.06	C5	DC2	0.06	0.00	0.03	0.08
P2	DT3	0.00	0.00	0.00	0.00	P2	DT3	0.00	0.00	0.00	0.01
PO2	DT3	0.00	0.00	0.00	0.01	PO2	DT3	0.00	0.00	0.00	0.00
PO1	DT3	0.00	0.00	0.00	0.00	PO1	DT3	0.00	0.00	0.00	0.01
O5'	DT3	0.00	0.00	0.00	0.00	O5'	DT3	0.00	0.00	0.00	0.01
O3'	DT3	0.00	0.00	0.00	0.00	O3'	DT3	0.00	0.00	0.00	0.00

C1'	DT_3	0.00	0.00	0.00	0.00	C1'	DT_3	0.00	0.00	0.00	0.00
C2'	DT_3	0.00	0.00	0.00	0.00	C2'	DT_3	0.00	0.00	0.00	-0.01
C3'	DT_3	0.00	0.00	0.00	0.00	C3'	DT_3	0.00	0.00	0.00	-0.01
C4'	DT_3	0.00	0.00	0.00	0.00	C4'	DT_3	0.00	0.00	0.00	0.00
C5'	DT_3	0.00	0.00	0.00	0.00	C5'	DT_3	0.00	0.00	0.00	0.00
O4'	DT_3	0.00	0.00	0.00	0.00	O4'	DT_3	0.00	0.00	0.00	0.00
O4	DT_3	0.02	0.00	0.01	0.02	O4	DT_3	0.02	0.00	0.01	0.00
C4	DT_3	0.01	0.00	0.01	0.01	C4	DT_3	0.01	0.00	0.01	0.00
N3	DT_3	0.01	0.00	0.00	0.01	N3	DT_3	0.01	0.00	0.00	0.00
C2	DT_3	0.00	0.00	0.00	0.00	C2	DT_3	0.00	0.00	0.00	0.00
O2	DT_3	0.01	0.00	0.01	0.02	O2	DT_3	0.01	0.00	0.01	-0.01
N1	DT_3	0.00	0.00	0.00	0.00	N1	DT_3	0.00	0.00	0.00	0.01
C6	DT_3	0.03	0.00	0.02	0.03	C6	DT_3	0.02	0.00	0.01	0.01
C5	DT_3	0.01	0.00	0.00	0.01	C5	DT_3	0.00	0.00	0.00	0.00
CM5	DT_3	0.03	0.00	0.02	0.02	CM5	DT_3	0.02	0.00	0.01	0.00
P3	DC_4	0.00	0.00	0.00	0.00	P3	DC_4	0.00	0.00	0.00	0.00
PO2	DC_4	0.00	0.00	0.00	0.00	PO2	DC_4	0.00	0.00	0.00	0.00
PO1	DC_4	0.00	0.00	0.00	0.00	PO1	DC_4	0.00	0.00	0.00	0.00
O5'	DC_4	0.00	0.00	0.00	0.00	O5'	DC_4	0.00	0.00	0.00	0.00
O3'	DC_4	0.00	0.00	0.00	0.00	O3'	DC_4	0.00	0.00	0.00	0.00
C1'	DC_4	0.00	0.00	0.00	0.00	C1'	DC_4	0.00	0.00	0.00	0.00
C2'	DC_4	0.00	0.00	0.00	0.00	C2'	DC_4	0.00	0.00	0.00	0.00
C3'	DC_4	0.00	0.00	0.00	0.00	C3'	DC_4	0.00	0.00	0.00	0.00
C4'	DC_4	0.00	0.00	0.00	0.00	C4'	DC_4	0.00	0.00	0.00	0.00
C5'	DC_4	0.00	0.00	0.00	0.00	C5'	DC_4	0.00	0.00	0.00	0.00
O4'	DC_4	0.00	0.00	0.00	0.00	O4'	DC_4	0.00	0.00	0.00	0.00
N4	DC_4	0.01	0.00	0.00	0.00	N4	DC_4	0.01	0.01	0.01	-0.01

C4	DC_4	0.00	0.00	0.00	0.00	C4	DC_4	0.00	0.00	0.00	0.00
N3	DC_4	0.00	0.00	0.00	0.00	N3	DC_4	0.00	0.00	0.00	0.00
C2	DC_4	0.00	0.00	0.00	0.00	C2	DC_4	0.00	0.00	0.00	0.00
O2	DC_4	0.00	0.00	0.00	0.01	O2	DC_4	0.00	0.00	0.00	0.00
N1	DC_4	0.00	0.00	0.00	0.00	N1	DC_4	0.00	0.00	0.00	0.00
C6	DC_4	0.00	0.00	0.00	0.00	C6	DC_4	0.01	0.00	0.01	0.00
C5	DC_4	0.00	0.00	0.00	0.00	C5	DC_4	0.01	0.01	0.01	0.00
P4	DT_5	0.00	0.00	0.00	0.00	P4	DT_5	0.00	0.00	0.00	0.00
PO2	DT_5	0.00	0.00	0.00	0.00	PO2	DT_5	0.00	0.00	0.00	0.00
PO1	DT_5	0.00	0.00	0.00	0.00	PO1	DT_5	0.00	0.00	0.00	0.00
O5'	DT_5	0.00	0.00	0.00	0.00	O5'	DT_5	0.00	0.00	0.00	0.00
O3'	DT_5	0.00	0.00	0.00	0.00	O3'	DT_5	0.00	0.00	0.00	0.00
C1'	DT_5	0.00	0.00	0.00	0.00	C1'	DT_5	0.00	0.00	0.00	0.00
C2'	DT_5	0.00	0.00	0.00	0.00	C2'	DT_5	0.00	0.00	0.00	0.00
C3'	DT_5	0.00	0.00	0.00	0.00	C3'	DT_5	0.00	0.00	0.00	0.00
C4'	DT_5	0.00	0.00	0.00	0.00	C4'	DT_5	0.00	0.00	0.00	0.00
C5'	DT_5	0.00	0.00	0.00	0.00	C5'	DT_5	0.00	0.00	0.00	0.00
O4'	DT_5	0.00	0.00	0.00	0.00	O4'	DT_5	0.00	0.00	0.00	0.00
O4	DT_5	0.00	0.00	0.00	0.01	O4	DT_5	0.00	0.00	0.00	0.00
C4	DT_5	0.00	0.00	0.00	0.00	C4	DT_5	0.00	0.00	0.00	0.00
N3	DT_5	0.00	0.00	0.00	0.00	N3	DT_5	0.00	0.00	0.00	0.00
C2	DT_5	0.00	0.00	0.00	0.00	C2	DT_5	0.00	0.00	0.00	0.00
O2	DT_5	0.00	0.00	0.00	0.01	O2	DT_5	0.00	0.00	0.00	0.00
N1	DT_5	0.00	0.00	0.00	-0.01	N1	DT_5	0.00	0.00	0.00	0.00
C6	DT_5	0.00	0.00	0.00	0.00	C6	DT_5	0.00	0.00	0.00	0.00
C5	DT_5	0.00	0.00	0.00	0.00	C5	DT_5	0.00	0.00	0.00	0.00
C6	DT_5	0.00	0.00	0.00	0.00	C6	DT_5	0.00	0.00	0.00	-0.01

O5'	DA_1	0.00	0.00	0.00	0.00	O5'	DA_1	0.00	0.00	0.00	0.00
O3'	DA_1	0.00	0.00	0.00	0.00	O3'	DA_1	0.00	0.00	0.00	0.00
C1'	DA_1	0.00	0.00	0.00	0.00	C1'	DA_1	0.00	0.00	0.00	-0.01
C2'	DA_1	0.00	0.00	0.00	0.00	C2'	DA_1	0.00	0.00	0.00	0.00
C3'	DA_1	0.00	0.00	0.00	0.00	C3'	DA_1	0.00	0.00	0.00	0.00
C4'	DA_1	0.00	0.00	0.00	0.00	C4'	DA_1	0.00	0.00	0.00	0.00
C5'	DA_1	0.00	0.00	0.00	0.00	C5'	DA_1	0.00	0.00	0.00	0.00
O4'	DA_1	0.00	0.00	0.00	0.00	O4'	DA_1	0.00	0.00	0.00	-0.01
N6	DA_1	0.00	0.00	0.00	0.00	N6	DA_1	0.00	0.02	0.01	-0.02
C6	DA_1	0.00	0.00	0.00	0.00	C6	DA_1	0.00	0.00	0.00	0.00
C5	DA_1	0.00	0.00	0.00	0.00	C5	DA_1	0.00	0.00	0.00	0.00
N7	DA_1	0.00	0.00	0.00	0.00	N7	DA_1	0.00	0.01	0.01	-0.01
C8	DA_1	0.00	0.00	0.00	0.00	C8	DA_1	0.00	0.01	0.01	-0.01
N9	DA_1	0.00	0.00	0.00	0.00	N9	DA_1	0.00	0.00	0.00	0.00
C4	DA_1	0.00	0.00	0.00	0.00	C4	DA_1	0.00	0.00	0.00	0.00
N3	DA_1	0.00	0.00	0.00	0.00	N3	DA_1	0.00	0.00	0.00	0.00
C2	DA_1	0.00	0.00	0.00	0.00	C2	DA_1	0.00	0.01	0.00	0.00
N3	DA_1	0.00	0.00	0.00	0.00	N3	DA_1	0.00	0.00	0.00	0.00
P5	DG_2	0.00	0.00	0.00	0.00	P5	$^{o}G_2$	0.00	0.00	0.00	0.02
PO2	DG_2	0.00	0.00	0.00	0.00	PO2	$^{o}G_2$	0.00	0.00	0.00	0.02
PO1	DG_2	0.00	0.00	0.00	0.00	PO1	$^{o}G_2$	0.00	0.00	0.00	0.07
O5'	DG_2	0.00	0.00	0.00	0.00	O5'	$^{o}G_2$	0.00	0.00	0.00	0.01
O3'	DG_2	0.00	0.00	0.00	0.00	O3'	$^{o}G_2$	0.00	0.00	0.00	0.00
C1'	DG_2	0.00	0.00	0.00	0.00	C1'	$^{o}G_2$	0.00	0.02	0.01	-0.01
C2'	DG_2	0.00	0.00	0.00	0.00	C2'	$^{o}G_2$	0.00	0.01	0.01	-0.02
C3'	DG_2	0.00	0.00	0.00	0.00	C3'	$^{o}G_2$	0.00	0.01	0.00	-0.01

C4'	DG_2	0.00	0.00	0.00	0.00	C4'	$^{O}G_2$	0.00	0.01	0.00	-0.01
C5'	DG_2	0.00	0.00	0.00	0.00	C5'	$^{O}G_2$	0.00	0.01	0.00	0.03
O4'	DG_2	0.00	0.00	0.00	0.00	O4'	$^{O}G_2$	0.00	0.00	0.00	0.00
N2	DG_2	0.00	0.00	0.00	0.00	N2	$^{O}G_2$	0.00	0.11	0.06	-0.13
O6	DG_2	0.00	0.00	0.00	0.00	O6	$^{O}G_2$	0.00	0.08	0.04	-0.09
C6	DG_2	0.00	0.00	0.00	0.00	C6	$^{O}G_2$	0.00	0.03	0.01	-0.04
C5	DG_2	0.00	0.00	0.00	0.00	C5	$^{O}G_2$	0.00	0.10	0.05	-0.10
N7	DG_2	0.00	0.00	0.00	0.00	N7	$^{O}G_2$	0.00	0.10	0.05	-0.11
C8	DG_2	0.00	0.00	0.00	0.00	C8	$^{O}G_2$	0.00	0.03	0.02	-0.05
N9	DG_2	0.00	0.00	0.00	0.00	O8	$^{O}G_2$	0.00	0.10	0.05	-0.12
C4	DG_2	0.00	0.00	0.00	0.00	N9	$^{O}G_2$	0.00	0.02	0.01	-0.02
N3	DG_2	0.00	0.00	0.00	0.00	C4	$^{O}G_2$	0.00	0.04	0.02	-0.04
C2	DG_2	0.00	0.00	0.00	0.00	N3	$^{O}G_2$	0.00	0.07	0.03	-0.07
N1	DG_2	0.00	0.00	0.00	0.00	C2	$^{O}G_2$	0.00	0.03	0.02	-0.03
						N1	$^{O}G_2$	0.00	0.02	0.01	-0.02
P6	rA_3	0.00	0.00	0.00	0.00						
PO2	rA_3	0.00	0.00	0.00	0.00	P6	rA_3	0.00	0.00	0.00	0.00
PO1	rA_3	0.00	0.00	0.00	0.00	PO2	rA_3	0.00	0.00	0.00	0.00
						PO1	rA_3	0.00	0.00	0.00	0.00
O5'	rA_3	0.00	0.00	0.00	0.00						
O3'	rA_3	0.00	0.00	0.00	0.00	O5'	rA_3	0.00	0.00	0.00	0.00
C1'	rA_3	0.00	0.00	0.00	0.00	O3'	rA_3	0.00	0.00	0.00	0.00
C2'	rA_3	0.00	0.00	0.00	0.00	C1'	rA_3	0.00	0.00	0.00	0.00
O2'	rA_3	0.00	0.01	0.01	-0.01	C2'	rA_3	0.00	0.00	0.00	-0.01
C3'	rA_3	0.00	0.00	0.00	0.00	O2'	rA_3	0.00	0.00	0.00	-0.02
C4'	rA_3	0.00	0.00	0.00	0.00	C3'	rA_3	0.00	0.00	0.00	0.02
C5'	rA_3	0.00	0.00	0.00	0.00	C4'	rA_3	0.00	0.00	0.00	0.00
O4'	rA_3	0.00	0.00	0.00	0.00	C5'	rA_3	0.00	0.00	0.00	0.00
N6	rA_3	0.00	0.02	0.01	-0.02	O4'	rA_3	0.00	0.00	0.00	0.00

C6	rA_3	0.00	0.00	0.00	0.00	N6	rA_3	0.00	0.01	0.01	0.06
C5	rA_3	0.00	0.00	0.00	0.00	C6	rA_3	0.00	-0.01	0.00	0.01
N7	rA_3	0.00	0.01	0.00	0.00	C5	rA_3	0.00	0.00	0.00	0.00
C8	rA_3	0.00	0.02	0.01	-0.02	N7	rA_3	0.00	0.00	0.00	-0.01
N9	rA_3	0.00	0.00	0.00	0.00	C8	rA_3	0.00	0.02	0.01	-0.02
C4	rA_3	0.00	0.00	0.00	0.00	N9	rA_3	0.00	0.00	0.00	0.00
N3	rA_3	0.00	0.01	0.01	-0.01	C4	rA_3	0.00	0.00	0.00	0.00
C2	rA_3	0.00	0.01	0.01	-0.01	N3	rA_3	0.00	0.01	0.01	-0.01
N1	rA_3	0.00	0.00	0.00	0.00	C2	rA_3	0.00	0.01	0.01	-0.01
						N1	rA_3	0.00	0.00	0.00	0.00
P7	$^{O}G_4$	0.00	0.00	0.00	0.00						
PO2	$^{O}G_4$	0.00	0.00	0.00	0.00	P7	DG_4	0.00	0.00	0.00	0.01
PO1	$^{O}G_4$	0.00	0.00	0.00	0.00	PO1	DG_4	0.00	0.00	0.00	-0.01
		0.00	0.00	0.00	0.00	PO2	DG_4	0.00	0.00	0.00	0.01
O5'	$^{O}G_4$	0.00	0.00	0.00	0.00						
O3'	$^{O}G_4$	0.00	0.00	0.00	0.00	O5'	DG_4	0.00	0.00	0.00	0.01
C1'	$^{O}G_4$	0.00	0.01	0.01	-0.01	O3'	DG_4	0.00	0.00	0.00	0.00
C2'	$^{O}G_4$	0.00	0.01	0.01	-0.01	C1'	DG_4	0.00	0.00	0.00	0.00
C3'	$^{O}G_4$	0.00	0.00	0.00	0.00	C2'	DG_4	0.00	0.00	0.00	0.00
C4'	$^{O}G_4$	0.00	0.01	0.00	-0.01	C3'	DG_4	0.00	0.00	0.00	0.00
C5'	$^{O}G_4$	0.00	0.01	0.00	-0.01	C4'	DG_4	0.00	0.00	0.00	0.00
O4'	$^{O}G_4$	0.00	0.00	0.00	0.00	C5'	DG_4	0.00	0.00	0.00	0.00
N2	$^{O}G_4$	0.01	0.11	0.06	-0.10	O4'	DG_4	0.00	0.00	0.00	-0.02
O6	$^{O}G_4$	0.00	0.09	0.05	-0.08	N2	DG_4	0.01	0.00	0.00	0.02
C6	$^{O}G_4$	0.00	0.03	0.01	-0.03	O6	DG_4	0.00	0.00	0.00	0.00
C5	$^{O}G_4$	0.00	0.10	0.05	-0.09	C6	DG_4	0.00	0.00	0.00	0.00
N7	$^{O}G_4$	0.00	0.09	0.04	-0.08	C5	DG_4	0.00	0.00	0.00	0.01
O8	$^{O}G_4$	0.00	0.08	0.04	-0.08	N7	DG_4	0.00	0.00	0.00	0.01
C8	$^{O}G_4$	0.00	0.03	0.02	-0.03	C8	DG_4	0.00	0.00	0.00	0.00

N9	$^{O}G_4$	0.00	0.01	0.01	-0.01	N9	DG_4	0.00	0.00	0.00	0.00
C4	$^{O}G_4$	0.00	0.04	0.02	-0.04	C4	DG_4	0.00	0.00	0.00	0.01
N3	$^{O}G_4$	0.00	0.07	0.04	-0.06	N3	DG_4	0.00	0.00	0.00	0.01
C2	$^{O}G_4$	0.00	0.03	0.01	-0.03	C2	DG_4	0.00	0.00	0.00	0.00
N1	$^{O}G_4$	0.00	0.02	0.01	-0.01	N1	DG_4	0.00	0.00	0.00	0.01
P8	DA_5	0.00	0.00	0.00	0.00	P8	DA_5	0.00	0.00	0.00	0.00
PO2	DA_5	0.00	0.00	0.00	0.00	PO2	DA_5	0.00	0.00	0.00	0.00
PO1	DA_5	0.00	0.00	0.00	0.00	PO1	DA_5	0.00	0.00	0.00	0.00
	DA_5	0.00	0.00	0.00	0.00						
O5'	DA_5	0.00	0.00	0.00	0.00	O5'	DA_5	0.00	0.00	0.00	0.00
O3'	DA_5	0.00	0.00	0.00	0.00	O3'	DA_5	0.00	0.00	0.00	0.00
C1'	DA_5	0.00	0.00	0.00	0.00	C1'	DA_5	0.00	0.00	0.00	0.00
C2'	DA_5	0.00	0.01	0.00	0.00	C2'	DA_5	0.00	0.00	0.00	0.00
C3'	DA_5	0.00	0.00	0.00	0.00	C3'	DA_5	0.00	0.00	0.00	0.00
C4'	DA_5	0.00	0.00	0.00	0.00	C4'	DA_5	0.00	0.00	0.00	0.00
C5'	DA_5	0.00	0.00	0.00	0.00	C5'	DA_5	0.00	0.00	0.00	0.00
O4'	DA_5	0.00	0.00	0.00	0.00	O4'	DA_5	0.00	0.00	0.00	0.00
C6	DA_5	0.00	0.00	0.00	0.00	C6	DA_5	0.00	0.00	0.00	0.00
C5	DA_5	0.00	0.00	0.00	0.00	C5	DA_5	0.00	0.00	0.00	0.00
N7	DA_5	0.00	0.00	0.00	0.00	N7	DA_5	0.00	0.00	0.00	0.00
C8	DA_5	0.00	0.01	0.01	0.00	C8	DA_5	0.00	0.00	0.00	0.00
N9	DA_5	0.00	0.00	0.00	0.00	N9	DA_5	0.00	0.00	0.00	0.00
C4	DA_5	0.00	0.00	0.00	0.00	C4	DA_5	0.00	0.00	0.00	0.00
N3	DA_5	0.00	0.00	0.00	0.00	N3	DA_5	0.00	0.00	0.00	0.00
C2	DA_5	0.00	0.01	0.00	-0.01	C2	DA_5	0.00	0.00	0.00	0.00
N1	DA_5	0.00	0.00	0.00	0.00	N1	DA_5	0.00	0.00	0.00	0.00
N6	DA_5	0.01	0.02	0.02	-0.01	N6	DA_5	0.01	0.00	0.01	0.01

Table S2 continued											
^{O}GA-DNA						rA^{O}G-DNA					
**Atom number**	**Nucleotide**	f_k^+	f_k^-	f_k^0	Δf_k	**Atom number**	**Nucleotide**	f_k^+	f_k^-	f_k^0	Δf_k
O5'	DT_1	0.00	0.00	0.00	0.00	O5'	DT_1	0.01	0.00	0.00	0.00
O3'	DT_1	0.00	0.00	0.00	0.00	O3'	DT_1	0.00	0.00	0.00	0.00
C1'	DT_1	0.00	0.00	0.00	0.00	C1'	DT_1	0.01	0.00	0.00	0.00
C2'	DT_1	0.00	0.00	0.00	0.00	C2'	DT_1	0.01	0.00	0.01	0.00
C3'	DT_1	0.00	0.00	0.00	0.00	C3'	DT_1	0.01	0.00	0.00	0.00
C4'	DT_1	0.00	0.00	0.00	0.00	C4'	DT_1	0.01	0.00	0.00	0.00
O4	DT_1	0.00	0.00	0.00	0.00	C5'	DT_1	0.01	0.00	0.01	0.00
C5'	DT_1	0.00	0.00	0.00	0.00	O4'	DT_1	0.01	0.00	0.00	0.01
O4'	DT_1	0.00	0.00	0.00	0.00	C4	DT_1	0.04	0.00	0.02	0.00
C4	DT_1	0.00	0.00	0.00	0.00	N3	DT_1	0.02	0.00	0.01	0.00
N3	DT_1	0.00	0.00	0.00	0.00	C2	DT_1	0.00	0.00	0.00	-0.01
C2	DT_1	0.00	0.00	0.00	0.00	O2	DT_1	0.02	0.00	0.01	0.01
O2	DT_1	0.00	0.00	0.00	0.00	N1	DT_1	0.01	0.00	0.01	-0.01
N1	DT_1	0.00	0.00	0.00	0.00	C6	DT_1	0.07	0.00	0.04	0.00
C6	DT_1	0.00	0.00	0.00	0.00	C5	DT_1	0.03	0.00	0.02	0.01
C5	DT_1	0.00	0.00	0.00	0.00	O4	DT_1	0.06	0.00	0.03	0.02
CM5	DT_1	0.00	0.00	0.00	0.00	CM5	DT_1	0.05	0.00	0.02	0.01
P1	DA_2	0.00	0.00	0.00	0.00	P1	DC_2	0.00	0.00	0.00	0.00
PO2	DA_2	0.00	0.00	0.00	0.00	PO2	DC_2	0.01	0.00	0.00	0.01
PO1	DA_2	0.00	0.00	0.00	0.00	PO1	DC_2	0.00	0.00	0.00	0.00

O5'	DA$_2$	0.00	0.00	0.00	0.00	O5'	DC$_2$	0.00	0.00	0.00	0.00
O3'	DA$_2$	0.00	0.00	0.00	0.00	O3'	DC$_2$	0.00	0.00	0.00	0.00
C1'	DA$_2$	0.00	0.00	0.00	0.00	C1'	DC$_2$	0.02	0.00	0.01	0.02
C2'	DA$_2$	0.00	0.00	0.00	0.00	C2'	DC$_2$	0.01	0.00	0.01	0.01
C3'	DA$_2$	0.00	0.00	0.00	0.00	C3'	DC$_2$	0.01	0.00	0.01	0.00
C4'	DA$_2$	0.00	0.00	0.00	0.00	C4'	DC$_2$	0.01	0.00	0.01	0.01
C5'	DA$_2$	0.00	0.00	0.00	0.00	C5'	DC$_2$	0.01	0.00	0.01	0.01
O4'	DA$_2$	0.00	0.00	0.00	0.00	O4'	DC$_2$	0.00	0.00	0.00	0.00
N6	DA$_2$	0.01	0.01	0.01	0.00	N4	DC$_2$	0.07	0.01	0.04	0.15
C6	DA$_2$	0.00	0.00	0.00	0.00	C4	DC$_2$	0.05	-0.01	0.02	0.11
C5	DA$_2$	-0.01	0.00	0.00	-0.01	N3	DC$_2$	0.03	0.00	0.02	0.04
N7	DA$_2$	0.00	0.00	0.00	0.00	C2	DC$_2$	0.01	0.00	0.01	0.02
C8	DA$_2$	0.01	0.01	0.01	0.00	O2	DC$_2$	0.03	0.01	0.02	0.04
N9	DA$_2$	0.00	0.00	0.00	0.00	N1	DC$_2$	0.02	0.01	0.01	0.02
C4	DA$_2$	0.00	0.00	0.00	0.00	C6	DC$_2$	0.12	0.01	0.06	0.14
N3	DA$_2$	0.01	0.01	0.01	0.00	C5	DC$_2$	0.07	0.00	0.04	0.06
C2	DA$_2$	0.01	0.01	0.01	0.00						
N1	DA$_2$	0.00	0.00	0.00	0.00	P2	$^{O}G_3$	0.00	0.00	0.00	0.00
						PO2	$^{O}G_3$	0.00	0.00	0.00	0.00
P2	DT$_3$	0.00	0.00	0.00	0.00	PO1	$^{O}G_3$	0.00	0.00	0.00	0.00
PO2	DT$_3$	0.00	0.00	0.00	0.00						
PO1	DT$_3$	0.00	0.00	0.00	0.00	O5'	$^{O}G_3$	0.00	0.00	0.00	0.00
						O3'	$^{O}G_3$	0.00	0.00	0.00	0.00
O5'	DT$_3$	0.00	0.00	0.00	0.00	C1'	$^{O}G_3$	0.00	0.01	0.01	-0.01
O3'	DT$_3$	0.00	0.00	0.00	0.00	C2'	$^{O}G_3$	0.00	0.01	0.01	-0.01
C1'	DT$_3$	0.01	0.00	0.01	0.01	C3'	$^{O}G_3$	0.00	0.01	0.00	-0.01
C2'	DT$_3$	0.02	0.00	0.01	0.02	C4'	$^{O}G_3$	0.00	0.01	0.01	-0.01
C3'	DT$_3$	0.01	0.00	0.00	0.01	C5'	$^{O}G_3$	0.00	0.01	0.01	0.00

C4'	DT_3	0.01	0.00	0.00	0.01	O4'	$^{O}G_3$	0.00	0.00	0.00	0.01
C5'	DT_3	0.01	0.00	0.00	0.01	N2	$^{O}G_3$	0.02	0.11	0.06	-0.09
O4'	DT_3	0.00	0.00	0.00	0.00	O6	$^{O}G_3$	0.01	0.09	0.05	-0.09
O4	DT_3	0.07	0.00	0.03	0.07	C6	$^{O}G_3$	0.00	0.04	0.02	-0.04
C4	DT_3	0.06	0.00	0.03	0.06	C5	$^{O}G_3$	0.00	0.09	0.04	-0.09
N3	DT_3	0.02	0.00	0.01	0.02	N7	$^{O}G_3$	0.00	0.08	0.04	-0.08
C2	DT_3	0.01	0.00	0.00	0.01	C8	$^{O}G_3$	0.00	0.03	0.02	-0.03
O2	DT_3	0.02	0.00	0.01	0.02	N9	$^{O}G_3$	0.00	0.02	0.01	-0.02
N1	DT_3	0.03	0.00	0.01	0.03	C4	$^{O}G_3$	0.00	0.05	0.02	-0.05
C6	DT_3	0.11	0.00	0.06	0.11	N3	$^{O}G_3$	0.00	0.05	0.03	-0.05
C5	DT_3	0.03	0.00	0.02	0.03	C2	$^{O}G_3$	0.01	0.04	0.02	-0.04
CM5	DT_3	0.05	0.00	0.03	0.05	N1	$^{O}G_3$	0.01	0.04	0.02	-0.04
						O8	$^{O}G_3$	0.01	0.10	0.06	-0.09
P3	DC_4	0.00	0.00	0.00	0.00						
PO2	DC_4	0.00	0.00	0.00	0.00	P3	DC_4	0.00	0.00	0.00	0.00
PO1	DC_4	0.00	0.00	0.00	0.00	PO2	DC_4	0.00	0.00	0.00	0.00
						PO1	DC_4	0.00	0.00	0.00	0.00
O5'	DC_4	0.00	0.00	0.00	0.00						
O3'	DC_4	0.00	0.00	0.00	0.00	O5'	DC_4	0.00	0.00	0.00	0.00
C1'	DC_4	0.01	0.00	0.00	0.01	O3'	DC_4	0.00	0.00	0.00	0.00
C2'	DC_4	0.01	0.00	0.01	0.01	C1'	DC_4	0.00	0.00	0.00	0.00
C3'	DC_4	0.01	0.00	0.00	0.00	C2'	DC_4	0.00	0.00	0.00	0.00
C4'	DC_4	0.01	0.00	0.00	0.01	C3'	DC_4	0.00	0.00	0.00	0.00
C5'	DC_4	0.00	0.00	0.00	0.00	C4'	DC_4	0.00	0.00	0.00	0.00
O4'	DC_4	0.00	0.00	0.00	0.00	C5'	DC_4	0.00	0.00	0.00	0.00
N4	DC_4	0.05	0.00	0.03	0.05	O4'	DC_4	0.00	0.00	0.00	0.00
C4	DC_4	0.03	0.00	0.01	0.03	N4	DC_4	0.00	0.01	0.01	-0.01
N3	DC_4	0.02	0.00	0.01	0.02	C4	DC_4	0.00	-0.01	0.00	0.01
C2	DC_4	0.01	0.00	0.00	0.01	N3	DC_4	0.00	0.00	0.00	0.00

O2	DC_4	0.02	0.00	0.01	0.02	C2	DC_4	0.00	0.00	0.00	0.00
N1	DC_4	0.02	0.00	0.01	0.02	O2	DC_4	0.00	0.01	0.00	0.00
C6	DC_4	0.08	0.00	0.04	0.08	N1	DC_4	0.00	0.00	0.00	0.00
C5	DC_4	0.04	0.00	0.02	0.04	C6	DC_4	0.00	0.00	0.00	-0.01
						C5	DC_4	0.00	0.00	0.00	0.00
P4	DT_5	0.00	0.00	0.00	0.00						
PO2	DT_5	0.00	0.00	0.00	0.00	P4	DT_5	0.00	0.00	0.00	0.00
PO1	DT_5	0.00	0.00	0.00	0.00	PO2	DT_5	0.00	0.00	0.00	0.00
						PO1	DT_5	0.00	0.00	0.00	0.00
O5'	DT_5	0.00	0.00	0.00	0.00						
O3'	DT_5	0.00	0.00	0.00	0.00	O5'	DT_5	0.00	0.00	0.00	0.00
C1'	DT_5	0.00	0.00	0.00	0.00	O3'	DT_5	0.00	0.00	0.00	0.00
C2'	DT_5	0.00	0.00	0.00	0.00	C1'	DT_5	0.00	0.00	0.00	0.00
C3'	DT_5	0.00	0.00	0.00	0.00	C2'	DT_5	0.00	0.00	0.00	0.00
C4'	DT_5	0.00	0.00	0.00	0.00	C3'	DT_5	0.00	0.00	0.00	0.00
C5'	DT_5	0.00	0.00	0.00	0.00	C4'	DT_5	0.00	0.00	0.00	0.00
O4'	DT_5	0.00	0.00	0.00	0.00	C5'	DT_5	0.00	0.00	0.00	0.00
O4	DT_5	0.01	0.00	0.00	0.01	O4'	DT_5	0.00	0.00	0.00	0.00
C4	DT_5	0.01	0.00	0.00	0.01	O4	DT_5	0.00	0.00	0.00	0.00
N3	DT_5	0.01	0.00	0.00	0.01	C4	DT_5	0.00	0.00	0.00	0.00
C2	DT_5	0.01	0.00	0.00	0.01	N3	DT_5	0.00	0.00	0.00	-0.01
O2	DT_5	0.01	0.00	0.00	0.00	C2	DT_5	0.00	0.00	0.00	-0.01
N1	DT_5	0.00	0.00	0.00	0.00	O2	DT_5	0.00	0.00	0.00	0.00
C6	DT_5	0.02	0.00	0.01	0.02	N1	DT_5	0.00	0.00	0.00	-0.01
C5	DT_5	0.00	0.00	0.00	0.00	C6	DT_5	0.00	0.00	0.00	-0.01
CM5	DT_5	0.02	0.00	0.01	0.02	C5	DT_5	0.00	0.00	0.00	0.00
						CM5	DT_5	0.00	0.00	0.00	-0.01
O5'	DA_1	0.00	0.00	0.00	0.00						
O3'	DA_1	0.00	0.00	0.00	0.00	O5'	DA_1	0.00	0.00	0.00	0.00

C1'	DA_1	0.00	0.00	0.00	0.00	O3'	DA_1	0.00	0.00	0.00	0.00
C2'	DA_1	0.00	0.00	0.00	0.00	C1'	DA_1	0.00	0.00	0.00	0.00
C3'	DA_1	0.00	0.00	0.00	0.00	C2'	DA_1	0.00	0.00	0.00	0.00
C4'	DA_1	0.00	0.00	0.00	0.00	C3'	DA_1	0.00	0.00	0.00	0.00
C5'	DA_1	0.00	0.00	0.00	0.00	C4'	DA_1	0.00	0.00	0.00	0.00
O4'	DA_1	0.00	0.00	0.00	0.00	C5'	DA_1	0.00	0.00	0.00	0.00
N6	DA_1	0.00	0.00	0.00	0.00	O4'	DA_1	0.00	0.00	0.00	0.00
C6	DA_1	0.00	0.00	0.00	0.00	N6	DA_1	0.00	0.00	0.00	0.00
C5	DA_1	0.00	0.00	0.00	0.00	C6	DA_1	0.00	0.00	0.00	0.00
N7	DA_1	0.00	0.00	0.00	0.00	C5	DA_1	0.00	0.00	0.00	0.00
C8	DA_1	0.00	0.00	0.00	0.00	N7	DA_1	0.00	0.00	0.00	0.00
N9	DA_1	0.00	0.00	0.00	0.00	C8	DA_1	0.00	0.00	0.00	0.00
C4	DA_1	0.00	0.00	0.00	0.00	N9	DA_1	0.00	0.00	0.00	0.00
N3	DA_1	0.00	0.00	0.00	0.00	C4	DA_1	0.00	0.00	0.00	0.00
C2	DA_1	0.00	0.00	0.00	0.00	N3	DA_1	0.00	0.00	0.00	0.00
N1	DA_1	0.00	0.00	0.00	0.00	C2	DA_1	0.00	0.00	0.00	0.00
						N1	DA_1	0.00	0.00	0.00	0.00
P5	DG_2	0.00	0.00	0.00	0.00						
PO2	DG_2	0.00	0.00	0.00	0.00	P5	DG_2	0.00	0.00	0.00	0.00
PO1	DG_2	0.00	0.00	0.00	0.00	PO2	DG_2	0.00	0.00	0.00	0.00
						PO1	DG_2	0.00	0.00	0.00	0.00
O5'	DG_2	0.00	0.00	0.00	0.00						
O3'	DG_2	0.00	0.00	0.00	0.00	O5'	DG_2	0.00	0.00	0.00	0.00
C1'	DG_2	0.00	0.00	0.00	0.00	O3'	DG_2	0.00	0.00	0.00	0.00
C2'	DG_2	0.00	0.00	0.00	0.00	C1'	DG_2	0.00	0.00	0.00	0.00
C3'	DG_2	0.00	0.00	0.00	0.00	C2'	DG_2	0.00	0.00	0.00	0.00
C4'	DG_2	0.00	0.00	0.00	0.00	C3'	DG_2	0.00	0.00	0.00	0.00
C5'	DG_2	0.00	0.00	0.00	0.00	C4'	DG_2	0.00	0.00	0.00	0.00
O4'	DG_2	0.00	0.00	0.00	0.00	C5'	DG_2	0.00	0.00	0.00	0.00

N2	DG_2	0.00	0.00	0.00	0.00	O4'	DG_2	0.00	0.00	0.00	0.00
O6	DG_2	0.00	0.00	0.00	0.00	N2	DG_2	0.00	0.00	0.00	0.00
C6	DG_2	0.00	0.00	0.00	0.00	O6	DG_2	0.00	0.00	0.00	0.01
C5	DG_2	0.00	0.00	0.00	0.00	C6	DG_2	0.00	0.00	0.00	0.00
N7	DG_2	0.00	0.00	0.00	0.00	C5	DG_2	0.00	0.00	0.00	0.00
C8	DG_2	0.01	0.00	0.00	0.00	N7	DG_2	0.00	0.00	0.00	0.01
N9	DG_2	0.00	0.00	0.00	0.00	C8	DG_2	0.00	0.00	0.00	0.00
C4	DG_2	0.00	0.00	0.00	0.00	N9	DG_2	0.00	0.00	0.00	0.00
N3	DG_2	0.00	0.00	0.00	0.00	C4	DG_2	0.00	0.00	0.00	0.00
C2	DG_2	0.00	0.00	0.00	0.00	N3	DG_2	0.00	0.00	0.00	0.00
N1	DG_2	0.00	0.00	0.00	0.00	C2	DG_2	0.00	0.00	0.00	0.00
						N1	DG_2	0.00	0.00	0.00	0.00
P6	rA_3	0.00	0.00	0.00	0.00						
PO2	rA_3	0.00	0.00	0.00	0.00	P6	rA_3	0.00	0.00	0.00	0.00
PO1	rA_3	0.00	0.00	0.00	0.00	PO2	rA_3	0.00	0.00	0.00	0.00
						PO1	rA_3	0.00	0.00	0.00	0.00
O5'	rA_3	0.00	0.00	0.00	0.00						
O3'	rA_3	0.00	0.00	0.00	0.00	O5'	rA_3	0.00	0.00	0.00	0.00
C1'	rA_3	0.00	0.00	0.00	0.00	O3'	rA_3	0.00	0.00	0.00	-0.01
C2'	rA_3	0.00	0.00	0.00	0.00	C1'	rA_3	0.00	0.00	0.00	0.00
O2'	rA_3	0.00	0.01	0.00	-0.01	C2'	rA_3	0.00	0.00	0.00	0.00
C3'	rA_3	0.00	0.00	0.00	0.00	O2'	rA_3	0.00	0.00	0.00	-0.02
C4'	rA_3	0.00	0.01	0.00	0.00	C3'	rA_3	0.00	0.00	0.00	0.00
C5'	rA_3	0.00	0.00	0.00	0.00	C4'	rA_3	0.00	0.00	0.00	0.00
O4'	rA_3	0.00	0.00	0.00	0.00	C5'	rA_3	0.00	0.00	0.00	0.00
N6	rA_3	0.01	0.02	0.02	-0.01	O4'	rA_3	0.00	0.00	0.00	0.00
C6	rA_3	0.00	0.00	0.00	0.00	N6	rA_3	0.00	0.01	0.01	-0.02
C5	rA_3	0.00	0.01	0.00	-0.01	C6	rA_3	0.00	0.00	0.00	0.00
N7	rA_3	0.00	0.01	0.01	-0.01	C5	rA_3	0.00	0.00	0.00	0.00

C8	rA_3	0.01	0.02	0.01	-0.01	N7	rA_3	0.00	0.00	0.00	0.00
N9	rA_3	0.01	0.00	0.00	0.01	C8	rA_3	0.00	0.01	0.00	0.00
C4	rA_3	0.00	0.00	0.00	0.01	N9	rA_3	0.00	0.00	0.00	0.00
N3	rA_3	0.01	0.00	0.01	0.01	C4	rA_3	0.00	0.00	0.00	0.00
C2	rA_3	0.00	0.01	0.00	0.00	N3	rA_3	0.00	0.01	0.00	0.00
N1	rA_3	-0.01	0.00	0.00	-0.01	C2	rA_3	0.00	0.00	0.00	0.00
						N1	rA_3	0.00	0.00	0.00	0.01
P7	$^{O}G_4$	0.00	0.00	0.00	0.00						
PO2	$^{O}G_4$	0.00	0.00	0.00	0.00	P7	DG_4	0.00	0.00	0.00	0.00
PO1	$^{O}G_4$	0.00	0.00	0.00	0.00	PO2	DG_4	0.00	0.00	0.00	0.01
						PO1	DG_4	0.00	0.00	0.00	0.00
O5'	$^{O}G_4$	0.00	0.00	0.00	0.00						
O3'	$^{O}G_4$	0.00	0.00	0.00	0.00	O5'	DG_4	0.00	0.00	0.00	0.00
C1'	$^{O}G_4$	0.00	0.01	0.01	-0.01	O3'	DG_4	0.00	0.00	0.00	0.00
C2'	$^{O}G_4$	0.00	0.01	0.00	-0.01	C1'	DG_4	0.00	0.00	0.00	-0.02
C3'	$^{O}G_4$	0.00	0.01	0.00	-0.01	C2'	DG_4	0.00	0.00	0.00	0.00
C4'	$^{O}G_4$	0.00	0.01	0.00	-0.01	C3'	DG_4	0.00	0.00	0.00	0.00
C5'	$^{O}G_4$	0.00	0.01	0.00	-0.01	C4'	DG_4	0.00	0.00	0.00	0.00
O4'	$^{O}G_4$	0.00	0.00	0.00	0.00	C5'	DG_4	0.00	0.00	0.00	0.00
N2	$^{O}G_4$	0.00	0.10	0.05	-0.09	O4'	DG_4	0.00	0.00	0.00	0.00
O6	$^{O}G_4$	0.00	0.08	0.04	-0.08	N2	DG_4	0.01	0.00	0.01	0.02
C6	$^{O}G_4$	0.00	0.03	0.02	-0.03	O6	DG_4	0.00	0.00	0.00	0.02
C5	$^{O}G_4$	0.00	0.08	0.04	-0.08	C6	DG_4	0.00	0.00	0.00	0.01
N7	$^{O}G_4$	0.00	0.07	0.04	-0.07	C5	DG_4	0.00	0.00	0.00	0.00
C8	$^{O}G_4$	0.00	0.03	0.02	-0.03	N7	DG_4	0.00	0.00	0.00	0.01
O8	$^{O}G_4$	0.00	0.12	0.06	-0.12	C8	DG_4	0.00	0.00	0.00	0.00
N9	$^{O}G_4$	0.00	0.02	0.01	-0.02	N9	DG_4	0.00	0.00	0.00	0.00
C4	$^{O}G_4$	0.00	0.05	0.02	-0.04	C4	DG_4	0.00	0.00	0.00	0.00
N3	$^{O}G_4$	0.00	0.04	0.02	-0.04	N3	DG_4	0.00	0.00	0.00	0.01

C2	$^{O}G_4$	0.00	0.03	0.02	-0.03	C2	DG_4	0.00	0.00	0.00	0.01
N1	$^{O}G_4$	0.00	0.04	0.02	-0.04	N1	DG_4	0.00	0.00	0.00	0.01
P8	DA_5	0.00	0.00	0.00	0.00	P8	DA_5	0.00	0.00	0.00	0.00
PO2	DA_5	0.00	0.00	0.00	0.00	PO2	DA_5	0.00	0.00	0.00	0.00
PO1	DA_5	0.00	0.00	0.00	0.00	PO1	DA_5	0.00	0.00	0.00	0.00
O5'	DA_5	0.00	0.00	0.00	0.00	O5'	DA_5	0.00	0.00	0.00	0.00
O3'	DA_5	0.00	0.00	0.00	0.00	O3'	DA_5	0.00	0.00	0.00	0.00
C1'	DA_5	0.00	0.00	0.00	0.00	C1'	DA_5	0.00	0.00	0.00	0.01
C2'	DA_5	0.00	0.01	0.00	-0.01	C2'	DA_5	0.00	0.00	0.00	0.00
C3'	DA_5	0.00	0.00	0.00	0.00	C3'	DA_5	0.00	0.00	0.00	0.00
C4'	DA_5	0.00	0.00	0.00	0.00	C4'	DA_5	0.00	0.00	0.00	0.00
C5'	DA_5	0.00	0.00	0.00	0.00	C5'	DA_5	0.00	0.00	0.00	0.00
O4'	DA_5	0.00	0.00	0.00	0.00	O4'	DA_5	0.00	0.00	0.00	0.01
C6	DA_5	0.00	0.00	0.00	0.00	C6	DA_5	0.00	0.00	0.00	0.00
C5	DA_5	0.00	0.00	0.00	0.00	C5	DA_5	0.00	0.00	0.00	0.00
N7	DA_5	0.00	0.00	0.00	0.00	N7	DA_5	0.00	0.00	0.00	0.01
C8	DA_5	0.00	0.01	0.00	0.00	C8	DA_5	0.00	0.00	0.00	0.00
N9	DA_5	0.00	0.00	0.00	0.00	N9	DA_5	0.00	0.00	0.00	0.00
C4	DA_5	0.00	0.00	0.00	0.00	C4	DA_5	0.00	0.00	0.00	0.00
N3	DA_5	0.00	0.01	0.00	0.00	N3	DA_5	0.00	0.00	0.00	0.00
C2	DA_5	0.00	0.01	0.01	-0.01	C2	DA_5	0.00	0.00	0.00	0.00
N1	DA_5	0.00	0.00	0.00	-0.01	N1	DA_5	0.00	0.00	0.00	0.00
N6	DA_5	0.00	0.01	0.01	-0.01	N6	DA_5	0.01	0.00	0.01	0.01

Table S3. The energies (in Hartree) of Neutral, Vertical Cation (VC^{NE}) (NE-non-equilibrated), Vertical Cation (VC^{EQ}) (EQ-equilibrated), Vertical Anion (VA^{NE}), Vertical Anion (VA^{EQ}), Adiabatic Cation (AC), Adiabatic Anion (AA) of complete DNA double helix calculated at the M06-2x/6-31++G** level of theory in the aqueous phase.

ds-oligo	**Neutral**	**VA^{NE}**	**VA^{EQ}**	**AA**	**VC^{NE}**	**VC^{EQ}**	**AC**
	**Complete DNA double helix**						
**^{O}GrAG-DNA**	-12926.83159	-12926.85829	-12926.88101	-12926.8808	-12926.61294	-12926.61294	-12926.58913
**GrA^{O}G-DNA**	-12926.84815	-12926.87719	-12926.9001	-12926.90191	-12926.59915	-12926.62337	-12926.62366
**rA^{O}G-DNA**	-13015.17373	-13015.19384	-13015.22415	-13015.2475	-13014.92526	-13014.9494	-13014.98224
**^{O}GA-DNA**	-12999.11582	-12999.15021	-12999.17147	-12999.2653	-12998.86919	-12998.89345	-12998.99121
**Energy (in Hartree) of 7,8-dihydro-8-oxo-2′-deoxyguanosine (^{O}G) calculated at M06-2x/6-31++G** level of theory in aqueous phase**							
Free nucleoside				Isolated from **^{O}GA-DNA**		Isolated from **GA^{O}G-DNA**	
syn:	-1038.5076590	anti:	-1038.5071990	syn:	-1038.5108420	anti:	-1038.5071990

Table S4. The energies (in Hartree) of Neutral, Vertical Cation (VC^{EQ}) (EQ-equilibrated), Vertical Anion (VA^{EQ}), Adiabatic Cation (AC), Adiabatic Anion (AA) of nucleosides pairs extracted from complete DNA double helix calculated at the M06-2x/6-31++G** level of theory in the aqueous phase.

Nucleosides pair	Neutral	VA^{EQ}	AA	VC^{EQ}	AC
		**rA^{O}G-DNA**			
**A_1T_5**	-1762.896561	-1762.946232	-1762.946574	-1762.654565	-1762.654166
**G_2C_4**	-1778.96083	-1779.014779	-1779.014593	-1778.734446	-1778.733805
**r$A_3$$^{O}G_3$**	-2001.731033	-2001.779837	-2001.784153	-2001.511085	-2001.541773
**G_4C_2**	-1778.948545	-1779.007178	-1779.022564	-1778.715957	-1778.718491
**A_5T_1**	-1762.8922	-1762.943455	-1762.942218	-1762.646272	-1762.646410
		**^{O}GA-DNA**			
**A_1T_5**	-1762.887282	-1762.946549	-1762.947385	-1762.644654	-1762.651008
**G_2C_4**	-1778.946932	-1779.006691	-1779.032554	-1778.720202	-1778.726941
**rA_3T_3**	-1838.08812	-1838.148661	-1838.155964	-1837.842571	-1837.850805
**G_4A_2**	-1926.530619	-1926.577542	-1926.584736	-1926.311298	-1926.347237
**A_5T_1**	-1762.886218	-1762.942907	-1762.944607	-1762.640285	-1762.64563
		**^{O}GrAG-DNA**			
**A_1T_5**	-1762.889595	-1762.944868	-1762.940169	-1762.646798	-1762.653658
**$^{O}G_2C_4$**	-1854.190122	-1854.247053	-1854.237312	-1853.971037	-1853.984141
**rA_3T_3**	-1838.104972	-1838.159465	-1838.146199	-1837.85915	-1837.852521
**G_4C_2**	-1778.949248	-1779.00694	-1779.025359	-1778.719461	-1778.723345
**A_5T_1**	-1762.893054	-1762.943734	-1762.941372	-1762.644403	-1762.645251
		**GrA^{O}G-DNA**			
**A_1T_5**	-1762.892446	-1762.947735	-1762.947866	-1762.649598	-1762.682082
**G_2C_4**	-1778.957658	-1779.01456	-1779.016265	-1778.7317	-1778.743985
**rA_3T_3**	-1838.109753	-1838.164325	-1838.163095	-1837.864774	-1837.866057
**$^{O}G_4C_2$**	-1854.187734	-1854.245876	-1854.259607	-1853.967916	-1853.99516
**A_5T_1**	-1762.892918	-1762.943601	-1762.941614	-1762.645515	-1762.68009

Table S5 Hirshfeld spin (S-H) and the charge model 5 based on the Hirshfeld methodology (Q-CM5), distribution of all have atoms presence in complete ds-DNAs, calculated at the M06-2x/6-31++G** level of theory in the aqueous phase. Vertical Cation (VC^{NC}) (NE-non-equilibrated), Vertical Cation (VC^{EQ}) (EQ-equilibrated), Adiabatic Cation (AC) and Vertical Anion (VA^{NE}), Vertical Anion (VA^{EQ}), Adiabatic Anion (AA)

^{O}GrAG-DNA														
**Atom number**	**Nucleotide**	**Neutral**	**^{NE}Vertical Anion**		**^{EQ}Vertical Anion**		**Adiabatic Anion**		**^{NE}Vertical Cation**		**^{EQ}Vertical Cation**		**Adiabatic C ation**	
		Q-CM5	S-H	Q-CM5	S-H	Q-CM5	S-H	Q-CM5	S-H	Q-CM5	S-H	Q-CM5	S-H	Q-CM5
O5'	DT_1	-0.0529	0.0005	-0.0537	0.0014	-0.0583	0.0000	-0.0557	0.0000	-0.0534	0.0000	-0.0526	0.0000	-0.0532
O3'	DT_1	-0.2402	0.0008	-0.2432	0.0001	-0.2425	0.0000	-0.2418	0.0000	-0.2394	0.0000	-0.2403	0.0000	-0.2404
C1'	DT_1	0.2375	0.0014	0.2378	0.0090	0.2304	0.0045	0.2349	0.0000	0.2383	0.0000	0.2377	0.0000	0.2389
C2'	DT_1	0.0481	0.0022	0.0496	0.0110	0.0390	0.0084	0.0450	0.0000	0.0462	0.0000	0.0478	0.0000	0.0493
C3'	DT_1	0.1397	0.0012	0.1365	0.0014	0.1334	0.0012	0.1356	0.0000	0.1403	0.0000	0.1397	0.0000	0.1403
C4'	DT_1	0.1365	0.0003	0.1329	0.0008	0.1304	0.0001	0.1316	0.0000	0.1382	0.0000	0.1367	0.0000	0.1366
C5'	DT_1	0.1738	0.0004	0.1685	0.0022	0.1646	0.0000	0.1695	0.0000	0.1753	0.0000	0.1737	0.0000	0.1741
O4'	DT_1	-0.2749	0.0003	-0.2778	0.0029	-0.2824	0.0001	-0.2800	0.0000	-0.2743	0.0000	-0.2747	0.0000	-0.2746
C4	DT_1	0.2551	0.0018	0.2549	0.0614	0.2214	0.0027	0.2527	0.0000	0.2557	0.0000	0.2555	0.0000	0.2553
N3	DT_1	-0.1080	0.0004	-0.1073	0.0137	-0.1214	0.0025	-0.1095	0.0000	-0.1083	0.0000	-0.1079	0.0000	-0.1086
C2	DT_1	0.3612	0.0008	0.3628	0.0065	0.3572	0.0032	0.3653	0.0000	0.3622	0.0000	0.3616	0.0000	0.3609
O2	DT_1	-0.3636	0.0003	-0.3633	0.0009	-0.3792	-0.0001	-0.3599	0.0000	-0.3627	0.0000	-0.3641	0.0000	-0.3629
N1	DT_1	-0.2742	0.0005	-0.2736	0.0120	-0.2800	0.0010	-0.2752	0.0000	-0.2732	0.0000	-0.2742	0.0000	-0.2744
C6	DT_1	0.1703	0.0022	0.1674	0.0948	0.1126	0.0007	0.1693	0.0000	0.1725	0.0000	0.1707	0.0000	0.1709
C5	DT_1	-0.0429	0.0015	-0.0452	0.0313	-0.0689	0.0022	-0.0472	0.0000	-0.0437	0.0000	-0.0429	0.0000	-0.0430
O4	DT_1	-0.3667	0.0021	-0.3711	0.0487	-0.4131	0.0052	-0.3724	0.0000	-0.3692	0.0000	-0.3673	0.0000	-0.3656
CM5	DT_1	0.0572	0.0006	0.0505	0.0063	0.0199	0.0005	0.0493	0.0000	0.0558	0.0000	0.0572	0.0000	0.0574
P1	DC_2	0.5354	0.0041	0.5312	0.0004	0.5329	0.0001	0.5315	0.0000	0.5361	0.0000	0.5354	0.0000	0.5354

PO2	DC_2	-0.4134	0.0028	-0.4193	0.0002	-0.4166	0.0000	-0.4179	0.0000	-0.4110	0.0000	-0.4132	0.0000	-0.4127
PO1	DC_2	0.0086	0.0061	0.0028	0.0004	0.0061	0.0001	0.0037	0.0000	0.0078	0.0000	0.0086	0.0000	0.0084
		0.1305	0.0130	0.1147	0.0011	0.1224	0.0003	0.1173	0.0000	0.1328	0.0000	0.1308	0.0000	0.1312
O5'	DC_2	-0.2365	0.0013	-0.2343	0.0006	-0.2366	0.0002	-0.2356	0.0000	-0.2374	0.0000	-0.2365	0.0000	-0.2356
O3'	DC_2	-0.2375	0.0039	-0.2439	0.0001	-0.2400	0.0000	-0.2398	0.0000	-0.2364	0.0000	-0.2374	0.0000	-0.2375
C1'	DC_2	0.2587	0.0038	0.2539	0.0099	0.2427	0.0157	0.2385	0.0000	0.2598	0.0000	0.2590	0.0000	0.2598
C2'	DC_2	0.0464	0.0054	0.0453	0.0102	0.0324	0.0099	0.0250	0.0000	0.0450	0.0000	0.0463	0.0000	0.0440
C3'	DC_2	0.1399	0.0048	0.1333	0.0017	0.1320	0.0011	0.1305	0.0000	0.1410	0.0000	0.1400	0.0000	0.1380
C4'	DC_2	0.1462	0.0014	0.1402	0.0004	0.1375	0.0007	0.1330	0.0000	0.1476	0.0000	0.1464	0.0000	0.1454
C5'	DC_2	0.2026	0.0029	0.1940	0.0011	0.1952	0.0010	0.1932	0.0000	0.2047	0.0000	0.2026	0.0000	0.2031
O4'	DC_2	-0.2321	0.0017	-0.2347	0.0050	-0.2329	0.0104	-0.2357	0.0000	-0.2315	0.0000	-0.2317	0.0000	-0.2335
N4	DC_2	0.0824	0.0170	0.0584	0.0512	0.0106	0.0612	-0.0435	0.0002	0.0758	0.0001	0.0826	0.0000	0.0801
C4	DC_2	0.2714	0.0295	0.2574	0.0985	0.2230	0.2052	0.1621	0.0000	0.2705	0.0000	0.2716	0.0000	0.2711
N3	DC_2	-0.3656	0.0096	-0.3712	0.0459	-0.3946	0.0886	-0.4040	0.0000	-0.3670	0.0000	-0.3656	0.0000	-0.3665
C2	DC_2	0.3542	0.0040	0.3529	0.0098	0.3431	0.0121	0.3313	0.0000	0.3550	0.0000	0.3545	0.0000	0.3533
O2	DC_2	-0.3562	0.0004	-0.3623	-0.0012	-0.3821	-0.0030	-0.4035	0.0000	-0.3534	0.0000	-0.3559	0.0000	-0.3565
N1	DC_2	-0.2626	0.0142	-0.2678	0.0533	-0.2850	0.1071	-0.2922	0.0000	-0.2612	0.0000	-0.2627	0.0000	-0.2629
C6	DC_2	0.2149	0.0435	0.1874	0.1774	0.1087	0.3882	0.0526	0.0000	0.2195	0.0000	0.2157	0.0000	0.2154
C5	DC_2	0.0116	0.0094	-0.0052	0.0365	-0.0517	0.0263	-0.0673	0.0000	0.0100	0.0000	0.0121	0.0000	0.0112
P2	DT_3	0.5405	0.0161	0.5262	0.0002	0.5384	0.0000	0.5348	0.0000	0.5416	0.0000	0.5406	0.0000	0.5393
PO2	DT_3	-0.4092	0.0097	-0.4235	0.0001	-0.4117	0.0000	-0.4142	0.0000	-0.4062	0.0000	-0.4087	0.0000	-0.4101
PO1	DT_3	-0.0082	0.0284	-0.0332	0.0003	-0.0105	0.0001	-0.0137	0.0000	-0.0087	0.0000	-0.0080	0.0000	-0.0093
O5'	DT_3	-0.2354	0.0036	-0.2331	0.0001	-0.2351	0.0000	-0.2406	0.0000	-0.2365	0.0000	-0.2355	0.0000	-0.2374
O3'	DT_3	-0.2394	0.0042	-0.2463	0.0000	-0.2403	0.0000	-0.2399	0.0000	-0.2385	0.0000	-0.2394	0.0000	-0.2390
C1'	DT_3	0.2586	0.0069	0.2519	0.0022	0.2549	0.0000	0.2545	0.0000	0.2595	0.0000	0.2592	0.0000	0.2597
C2'	DT_3	0.0469	0.0170	0.0391	0.0028	0.0426	0.0000	0.0517	0.0000	0.0458	0.0000	0.0472	0.0000	0.0470
C3'	DT_3	0.1375	0.0094	0.1273	0.0004	0.1351	0.0000	0.1422	0.0000	0.1394	0.0000	0.1379	0.0000	0.1385

C4'	DT$_3$	0.1418	0.0024	0.1328	0.0001	0.1391	0.0000	0.1433	0.0000	0.1435	0.0000	0.1423	0.0000	0.1435
C5'	DT$_3$	0.2048	0.0058	0.1906	0.0002	0.2013	0.0000	0.2019	0.0000	0.2074	0.0000	0.2050	0.0000	0.2075
O4'	DT$_3$	-0.2510	0.0023	-0.2558	0.0012	-0.2519	0.0001	-0.2564	0.0000	-0.2502	0.0000	-0.2505	0.0000	-0.2478
O4	DT$_3$	-0.3430	0.0243	-0.3666	0.0188	-0.3587	0.0009	-0.3419	0.0002	-0.3504	0.0002	-0.3438	0.0002	-0.3423
C4	DT$_3$	0.2576	0.0289	0.2448	0.0287	0.2467	0.0010	0.2585	0.0000	0.2580	0.0000	0.2583	0.0001	0.2574
N3	DT$_3$	-0.1002	0.0067	-0.1058	0.0091	-0.1079	0.0013	-0.1015	0.0001	-0.1035	0.0001	-0.0994	0.0002	-0.0999
C2	DT$_3$	0.3638	0.0059	0.3646	0.0028	0.3618	0.0006	0.3641	0.0000	0.3649	0.0000	0.3644	0.0000	0.3643
O2	DT$_3$	-0.3529	0.0011	-0.3638	0.0001	-0.3626	0.0005	-0.3474	0.0000	-0.3536	0.0000	-0.3526	0.0000	-0.3452
N1	DT$_3$	-0.2711	0.0097	-0.2720	0.0075	-0.2751	0.0001	-0.2767	0.0001	-0.2682	0.0000	-0.2706	0.0000	-0.2709
C6	DT$_3$	0.1924	0.0338	0.1695	0.0266	0.1714	0.0016	0.1844	0.0000	0.2001	0.0000	0.1951	0.0000	0.1940
C5	DT$_3$	-0.0401	0.0132	-0.0475	0.0061	-0.0420	0.0003	-0.0365	0.0000	-0.0404	0.0000	-0.0398	0.0000	-0.0390
CM5	DT$_3$	0.0528	0.0166	0.0247	0.0092	0.0302	0.0150	0.0492	0.0000	0.0560	0.0000	0.0548	0.0000	0.0565
P3	DC$_4$	0.5372	0.0163	0.5224	0.0001	0.5363	0.0000	0.5369	0.0000	0.5385	0.0000	0.5373	0.0000	0.5384
PO2	DC$_4$	-0.4129	0.0099	-0.4307	0.0000	-0.4138	0.0000	-0.4102	0.0000	-0.4092	0.0000	-0.4124	0.0000	-0.4111
PO1	DC$_4$	0.0086	0.0311	-0.0187	0.0001	0.0079	0.0000	0.0087	0.0000	0.0091	0.0000	0.0089	0.0000	0.0100
O5'	DC$_4$	-0.2338	0.0043	-0.2322	0.0000	-0.2337	0.0000	-0.2338	0.0000	-0.2346	0.0000	-0.2337	0.0000	-0.2340
O3'	DC$_4$	-0.2370	0.0017	-0.2411	0.0000	-0.2375	0.0000	-0.2375	0.0000	-0.2366	0.0000	-0.2369	0.0000	-0.2365
C1'	DC$_4$	0.2577	0.0089	0.2447	0.0011	0.2555	0.0000	0.2587	0.0000	0.2580	0.0000	0.2594	0.0000	0.2639
C2'	DC$_4$	0.0323	0.0109	0.0202	0.0012	0.0302	0.0000	0.0294	0.0000	0.0333	0.0000	0.0329	0.0000	0.0340
C3'	DC$_4$	0.1326	0.0055	0.1254	0.0002	0.1314	0.0000	0.1312	0.0000	0.1349	0.0000	0.1331	0.0000	0.1335
C4'	DC$_4$	0.1437	0.0027	0.1329	0.0000	0.1423	0.0000	0.1424	0.0000	0.1449	0.0000	0.1445	0.0000	0.1452
C5'	DC$_4$	0.2031	0.0068	0.1875	0.0002	0.2014	0.0000	0.2023	0.0000	0.2060	0.0000	0.2036	0.0000	0.2036
O4'	DC$_4$	-0.2386	0.0045	-0.2382	0.0007	-0.2383	0.0000	-0.2380	0.0000	-0.2383	0.0000	-0.2380	0.0000	-0.2363
N4	DC$_4$	0.0787	0.0342	0.0336	0.0038	0.0710	0.0001	0.0836	0.0006	0.0815	0.0004	0.0880	0.0001	0.0988
C4	DC$_4$	0.2698	0.0692	0.2388	0.0113	0.2664	0.0000	0.2712	0.0000	0.2708	0.0000	0.2715	0.0000	0.2746
N3	DC$_4$	-0.3681	0.0304	-0.3881	0.0061	-0.3714	0.0000	-0.3674	0.0000	-0.3741	-0.0001	-0.3691	-0.0001	-0.3627
C2	DC$_4$	0.3498	0.0070	0.3424	0.0009	0.3485	0.0000	0.3497	0.0000	0.3515	-0.0001	0.3516	0.0000	0.3578

O2	DC_4	-0.3649	-0.0002	-0.3919	-0.0003	-0.3698	0.0000	-0.3644	0.0004	-0.3668	0.0002	-0.3635	0.0000	-0.3565
N1	DC_4	-0.2646	0.0359	-0.2775	0.0072	-0.2672	0.0000	-0.2661	0.0002	-0.2605	0.0001	-0.2631	0.0000	-0.2601
C6	DC_4	0.2163	0.1189	0.1456	0.0212	0.2055	0.0000	0.2126	0.0000	0.2277	0.0000	0.2210	0.0000	0.2247
C5	DC_4	0.0158	0.0365	-0.0239	0.0014	0.0084	0.0001	0.0147	0.0003	0.0226	0.0001	0.0209	0.0000	0.0235
P4	DT_5	0.5374	0.0066	0.5299	0.0000	0.5371	0.0000	0.5363	0.0000	0.5391	0.0000	0.5376	0.0000	0.5382
PO2	DT_5	-0.4140	0.0039	-0.4243	0.0000	-0.4143	0.0000	-0.4132	0.0000	-0.4100	0.0000	-0.4135	0.0000	-0.4125
PO1	DT_5	-0.0126	0.0093	-0.0207	0.0000	-0.0129	0.0000	-0.0117	0.0000	-0.0116	0.0000	-0.0122	0.0000	-0.0099
O5'	DT_5	-0.2411	0.0012	-0.2419	0.0000	-0.2411	0.0000	-0.2421	0.0000	-0.2411	0.0000	-0.2410	0.0000	-0.2413
O3'	DT_5	-0.1031	0.0007	-0.1090	0.0000	-0.1032	0.0000	-0.1025	0.0000	-0.0999	0.0000	-0.1025	0.0000	-0.1016
C1'	DT_5	0.2613	0.0012	0.2575	0.0001	0.2609	0.0000	0.2618	0.0000	0.2595	0.0000	0.2620	0.0000	0.2644
C2'	DT_5	0.0709	0.0017	0.0635	0.0001	0.0702	0.0000	0.0724	0.0000	0.0726	0.0000	0.0718	0.0000	0.0739
C3'	DT_5	0.1188	0.0014	0.1156	0.0000	0.1184	0.0000	0.1182	0.0000	0.1214	0.0000	0.1191	0.0000	0.1189
C4'	DT_5	0.1334	0.0006	0.1293	0.0000	0.1331	0.0000	0.1330	0.0000	0.1333	0.0000	0.1337	0.0000	0.1340
C5'	DT_5	0.2061	0.0024	0.1995	0.0000	0.2055	0.0000	0.2061	0.0000	0.2081	0.0000	0.2063	0.0000	0.2064
O4'	DT_5	-0.2420	0.0006	-0.2398	0.0000	-0.2418	0.0000	-0.2415	0.0000	-0.2426	0.0000	-0.2417	0.0000	-0.2419
O4	DT_5	-0.3489	0.0031	-0.3496	0.0001	-0.3499	0.0000	-0.3451	0.0000	-0.3493	0.0000	-0.3485	0.0000	-0.3431
C4	DT_5	0.2592	0.0074	0.2600	0.0008	0.2598	0.0000	0.2590	0.0000	0.2606	0.0000	0.2601	0.0000	0.2624
N3	DT_5	-0.0999	0.0043	-0.1017	0.0006	-0.1000	0.0000	-0.1026	0.0001	-0.1039	0.0000	-0.0993	0.0002	-0.0956
C2	DT_5	0.3688	0.0013	0.3682	0.0001	0.3692	0.0000	0.3673	0.0001	0.3695	0.0000	0.3699	0.0003	0.3710
O2	DT_5	-0.3641	0.0002	-0.3752	0.0000	-0.3658	0.0000	-0.3629	0.0011	-0.3707	0.0010	-0.3643	0.0015	-0.3614
N1	DT_5	-0.2689	0.0035	-0.2725	0.0004	-0.2694	0.0000	-0.2708	0.0003	-0.2665	0.0002	-0.2681	0.0001	-0.2678
C6	DT_5	0.1977	0.0081	0.1852	0.0007	0.1966	0.0000	0.1991	0.0000	0.2050	0.0000	0.1999	0.0000	0.2019
C5	DT_5	-0.0378	0.0035	-0.0340	0.0001	-0.0373	0.0000	-0.0375	0.0002	-0.0361	0.0001	-0.0371	0.0001	-0.0368
C6	DT_5	0.0612	0.0194	0.0394	0.0009	0.0588	0.0000	0.0666	0.0001	0.0690	0.0000	0.0638	0.0000	0.0678
O5'	DA_1	-0.0500	0.0001	-0.0496	0.0000	-0.0496	0.0000	-0.0494	0.0000	-0.0485	0.0000	-0.0478	0.0000	-0.0468
O3'	DA_1	-0.2481	0.0000	-0.2491	0.0000	-0.2483	0.0000	-0.2484	0.0000	-0.2441	0.0000	-0.2463	0.0000	-0.2388

C1'	DA_1	0.2468	0.0000	0.2462	0.0000	0.2469	0.0000	0.2522	0.0002	0.2473	0.0001	0.2497	0.0001	0.2445
C2'	DA_1	0.0331	0.0000	0.0330	0.0000	0.0327	0.0000	0.0335	0.0017	0.0260	0.0015	0.0319	0.0012	0.0295
C3'	DA_1	0.1332	0.0000	0.1318	0.0000	0.1330	0.0000	0.1299	0.0001	0.1386	0.0001	0.1368	0.0001	0.1397
C4'	DA_1	0.1362	0.0000	0.1341	0.0000	0.1361	0.0000	0.1357	0.0001	0.1442	0.0001	0.1397	0.0000	0.1407
C5'	DA_1	0.1741	0.0001	0.1719	0.0000	0.1737	0.0000	0.1731	0.0001	0.1834	0.0001	0.1776	0.0001	0.1778
O4'	DA_1	-0.2742	0.0000	-0.2752	0.0000	-0.2742	0.0000	-0.2694	0.0002	-0.2682	0.0002	-0.2699	0.0002	-0.2721
N6	DA_1	0.0330	0.0045	0.0343	0.0004	0.0325	0.0000	0.0320	0.0163	0.0615	0.0105	0.0540	0.0092	0.0600
C6	DA_1	0.2545	0.0010	0.2564	0.0001	0.2547	0.0000	0.2547	0.0027	0.2547	0.0013	0.2549	0.0025	0.2531
C5	DA_1	0.0596	0.0002	0.0595	0.0000	0.0593	0.0000	0.0592	0.0146	0.0606	0.0106	0.0614	0.0198	0.0609
N7	DA_1	-0.4049	0.0003	-0.4037	0.0000	-0.4048	0.0000	-0.4063	0.0030	-0.3950	0.0012	-0.3944	0.0003	-0.3943
C8	DA_1	0.2824	0.0003	0.2770	0.0000	0.2821	0.0000	0.2800	0.0125	0.2975	0.0094	0.2945	0.0142	0.2952
N9	DA_1	-0.2637	0.0000	-0.2657	0.0000	-0.2637	0.0000	-0.2649	0.0014	-0.2652	0.0013	-0.2648	0.0015	-0.2632
C4	DA_1	0.2171	0.0003	0.2148	0.0000	0.2170	0.0000	0.2158	0.0075	0.2130	0.0054	0.2143	0.0105	0.2175
N3	DA_1	-0.3867	0.0001	-0.3923	0.0000	-0.3869	0.0000	-0.3881	0.0008	-0.3855	0.0002	-0.3826	0.0019	-0.3827
C2	DA_1	0.2887	0.0002	0.2867	0.0000	0.2880	0.0000	0.2855	0.0014	0.2948	0.0004	0.2948	0.0043	0.3060
N3	DA_1	-0.3565	0.0008	-0.3541	0.0000	-0.3568	0.0000	-0.3571	-0.0008	-0.3542	-0.0009	-0.3553	-0.0009	-0.3583
P5	$^{O}G_2$	0.5368	0.0000	0.5360	0.0000	0.5368	0.0000	0.5109	0.0000	0.5406	0.0000	0.5386	0.0000	0.5357
PO2	$^{O}G_2$	-0.4277	0.0000	-0.4298	0.0000	-0.4277	0.0000	-0.4467	0.0000	-0.4176	0.0000	-0.4249	0.0000	-0.4120
PO1	$^{O}G_2$	-0.0344	0.0000	-0.0342	0.0000	-0.0343	0.0000	-0.1046	0.0000	-0.0346	0.0000	-0.0318	0.0000	-0.0113
O5'	$^{O}G_2$	-0.2356	0.0000	-0.2356	0.0000	-0.2357	0.0000	-0.2433	0.0000	-0.2383	0.0000	-0.2362	0.0000	-0.2387
O3'	$^{O}G_2$	-0.2414	0.0000	-0.2426	0.0000	-0.2418	0.0000	-0.2414	0.0000	-0.2387	0.0000	-0.2396	0.0000	-0.2406
C1'	$^{O}G_2$	0.2574	0.0001	0.2555	0.0000	0.2572	0.0000	0.2551	-0.0002	0.2697	-0.0008	0.2738	-0.0013	0.2712
C2'	$^{O}G_2$	0.0436	0.0003	0.0416	0.0000	0.0432	0.0000	0.0489	0.0009	0.0535	0.0005	0.0551	-0.0001	0.0561
C3'	$^{O}G_2$	0.1540	0.0000	0.1523	0.0000	0.1539	0.0000	0.1596	0.0001	0.1617	0.0000	0.1604	-0.0001	0.1584
C4'	$^{O}G_2$	0.1502	0.0000	0.1488	0.0000	0.1500	0.0000	0.1518	0.0001	0.1582	0.0000	0.1577	0.0000	0.1564
C5'	$^{O}G_2$	0.2349	0.0000	0.2329	0.0000	0.2346	0.0000	0.1935	0.0000	0.2449	0.0000	0.2413	0.0000	0.2221
O4'	$^{O}G_2$	-0.2411	0.0000	-0.2406	0.0000	-0.2410	0.0000	-0.2430	0.0003	-0.2413	0.0000	-0.2377	-0.0003	-0.2342

N2	$^{O}G_2$	0.0434	0.0009	0.0379	0.0001	0.0414	0.0000	0.0653	0.0875	0.1468	0.0984	0.1563	0.0876	0.1432
O6	$^{O}G_2$	-0.3649	0.0015	-0.3594	0.0002	-0.3650	0.0000	-0.3605	0.0935	-0.2863	0.1044	-0.2808	0.0951	-0.2952
C6	$^{O}G_2$	0.2410	0.0038	0.2413	0.0002	0.2412	0.0000	0.2463	0.0342	0.2663	0.0322	0.2708	0.0360	0.2678
C5	$^{O}G_2$	0.0489	0.0006	0.0483	0.0000	0.0487	0.0000	0.0522	0.2243	0.1372	0.2479	0.1448	0.2641	0.1401
N7	$^{O}G_2$	-0.0460	0.0002	-0.0462	0.0000	-0.0461	0.0000	-0.0291	0.1196	0.0362	0.1342	0.0517	0.1436	0.0340
C8	$^{O}G_2$	0.3517	0.0004	0.3499	0.0000	0.3518	0.0000	0.3709	0.0218	0.3788	0.0163	0.3840	0.0244	0.3792
O8	$^{O}G_2$	-0.3757	0.0002	-0.3819	0.0000	-0.3768	0.0000	-0.3465	0.0940	-0.2854	0.1109	-0.2807	0.0927	-0.2857
N9	$^{O}G_2$	-0.2627	0.0006	-0.2654	0.0000	-0.2632	0.0000	-0.2575	0.0072	-0.2490	0.0021	-0.2473	-0.0041	-0.2531
C4	$^{O}G_2$	0.2277	0.0033	0.2224	0.0002	0.2273	0.0000	0.2292	0.0586	0.2631	0.0571	0.2693	0.0600	0.2704
N3	$^{O}G_2$	-0.3948	0.0003	-0.4029	0.0000	-0.3957	0.0000	-0.3953	0.0819	-0.3345	0.0848	-0.3277	0.0871	-0.3405
C2	$^{O}G_2$	0.3724	0.0014	0.3714	0.0001	0.3725	0.0000	0.3753	0.0248	0.3999	0.0217	0.4032	0.0241	0.3974
N1	$^{O}G_2$	-0.0870	0.0046	-0.0880	0.0004	-0.0878	0.0000	-0.0866	-0.0054	-0.0690	-0.0098	-0.0678	-0.0097	-0.0812
P6	rA_3	0.5290	0.0000	0.5285	0.0000	0.5293	0.0000	0.5284	0.0000	0.5320	0.0000	0.5309	0.0000	0.5306
PO2	rA_3	-0.4532	0.0000	-0.4546	0.0000	-0.4532	0.0000	-0.4525	0.0000	-0.4456	0.0000	-0.4509	0.0000	-0.4504
PO1	rA_3	0.0107	0.0000	0.0100	0.0000	0.0105	0.0000	0.0087	0.0000	0.0128	0.0000	0.0121	0.0000	0.0115
O5'	rA_3	-0.2366	0.0000	-0.2369	0.0000	-0.2367	0.0000	-0.2373	0.0000	-0.2368	0.0000	-0.2366	0.0000	-0.2359
O3'	rA_3	-0.2128	0.0000	-0.2130	0.0000	-0.2130	0.0000	-0.2131	0.0000	-0.2135	0.0000	-0.2128	0.0000	-0.2148
C1'	rA_3	0.2410	0.0001	0.2408	0.0000	0.2406	0.0000	0.2414	0.0000	0.2433	0.0000	0.2438	0.0000	0.2427
C2'	rA_3	0.1271	0.0001	0.1255	0.0000	0.1266	0.0000	0.1322	0.0000	0.1285	0.0000	0.1284	0.0000	0.1308
O2'	rA_3	-0.1070	0.0002	-0.1082	0.0000	-0.1075	0.0000	-0.0931	0.0001	-0.1029	0.0001	-0.1046	0.0001	-0.0821
C3'	rA_3	0.1758	0.0000	0.1734	0.0000	0.1753	0.0000	0.1554	0.0000	0.1812	0.0000	0.1778	0.0000	0.1588
C4'	rA_3	0.1610	0.0000	0.1603	0.0000	0.1606	0.0000	0.1563	0.0000	0.1639	0.0000	0.1631	0.0000	0.1607
C5'	rA_3	0.2276	0.0000	0.2256	0.0000	0.2272	0.0000	0.2266	0.0000	0.2319	0.0000	0.2300	0.0000	0.2297
O4'	rA_3	-0.2533	0.0000	-0.2536	0.0000	-0.2534	0.0000	-0.2530	0.0001	-0.2569	0.0000	-0.2517	0.0001	-0.2519
N6	rA_3	0.0644	0.0025	0.0627	0.0017	0.0609	0.0084	-0.0087	0.0142	0.0785	0.0069	0.0793	0.0026	0.0757
C6	rA_3	0.2615	0.0035	0.2614	0.0009	0.2615	0.0013	0.2577	0.0042	0.2554	0.0019	0.2563	0.0013	0.2584
C5	rA_3	0.0645	0.0008	0.0633	0.0002	0.0639	0.0001	0.0681	0.0185	0.0612	0.0124	0.0613	0.0071	0.0607

N7	rA_3	-0.3815	0.0008	-0.3825	0.0001	-0.3822	0.0002	-0.3774	0.0015	-0.3845	-0.0002	-0.3784	-0.0001	-0.3800
C8	rA_3	0.3133	0.0033	0.3026	0.0007	0.3105	0.0002	0.3142	0.0131	0.3338	0.0096	0.3285	0.0056	0.3229
N9	rA_3	-0.2600	0.0004	-0.2634	0.0001	-0.2609	0.0000	-0.2605	0.0006	-0.2554	0.0001	-0.2573	0.0002	-0.2572
C4	rA_3	0.2186	0.0003	0.2160	0.0001	0.2179	0.0000	0.2186	0.0103	0.2217	0.0077	0.2206	0.0063	0.2188
N3	rA_3	-0.3822	0.0022	-0.3885	0.0006	-0.3846	0.0001	-0.3836	0.0122	-0.3688	0.0080	-0.3721	0.0042	-0.3764
C2	rA_3	0.2916	0.0039	0.2928	0.0005	0.2891	0.0000	0.2920	0.0078	0.3067	0.0051	0.3039	0.0036	0.2990
N1	rA_3	-0.3529	0.0004	-0.3477	0.0001	-0.3526	0.0004	-0.3522	0.0041	-0.3497	0.0026	-0.3511	0.0016	-0.3521
P7	DG_4	0.5431	0.0000	0.5425	0.0000	0.5431	0.0000	0.5278	0.0000	0.5445	0.0000	0.5437	0.0000	0.5332
PO1	DG_4	-0.0233	0.0000	-0.0259	0.0000	-0.0237	0.0000	-0.0184	0.0000	-0.0192	0.0000	-0.0224	0.0000	-0.0104
PO2	DG_4	-0.3738	0.0000	-0.3735	0.0000	-0.3736	0.0000	-0.3873	0.0000	-0.3719	0.0000	-0.3731	0.0000	-0.3781
O5'	DG_4	-0.2464	0.0000	-0.2479	0.0000	-0.2466	0.0000	-0.2538	0.0000	-0.2434	0.0000	-0.2458	0.0000	-0.2483
O3'	DG_4	-0.2399	0.0000	-0.2394	0.0000	-0.2400	0.0000	-0.2390	0.0000	-0.2401	0.0000	-0.2398	0.0000	-0.2390
C1'	DG_4	0.2601	0.0002	0.2616	0.0000	0.2595	0.0000	0.2614	0.0000	0.2603	0.0000	0.2609	0.0000	0.2627
C2'	DG_4	0.0285	0.0000	0.0266	0.0000	0.0278	0.0000	0.0323	0.0000	0.0315	0.0000	0.0288	0.0000	0.0310
C3'	DG_4	0.1267	0.0000	0.1247	0.0000	0.1261	0.0000	0.1295	0.0000	0.1288	0.0000	0.1269	0.0000	0.1286
C4'	DG_4	0.1345	0.0002	0.1350	0.0000	0.1341	0.0000	0.1372	0.0000	0.1341	0.0000	0.1349	0.0000	0.1366
C5'	DG_4	0.2040	0.0001	0.2027	0.0000	0.2033	0.0000	0.1995	0.0000	0.2041	0.0000	0.2043	0.0000	0.2037
O4'	DG_4	-0.2516	0.0000	-0.2510	0.0000	-0.2517	0.0000	-0.2359	0.0000	-0.2552	0.0000	-0.2516	0.0000	-0.2444
N2	DG_4	0.0352	0.0010	0.0383	0.0009	0.0294	0.0011	0.0147	0.0004	0.0407	0.0001	0.0373	0.0000	0.0408
O6	DG_4	-0.3721	0.0003	-0.3728	0.0001	-0.3741	0.0000	-0.3693	0.0001	-0.3731	0.0002	-0.3712	0.0001	-0.3690
C6	DG_4	0.2443	0.0007	0.2447	0.0006	0.2436	0.0008	0.2411	0.0003	0.2420	0.0001	0.2435	0.0001	0.2434
C5	DG_4	0.0396	0.0001	0.0385	0.0000	0.0382	-0.0001	0.0346	0.0007	0.0358	0.0002	0.0380	0.0001	0.0365
N7	DG_4	-0.3878	0.0001	-0.3895	0.0001	-0.3893	0.0000	-0.3935	0.0001	-0.3907	0.0001	-0.3876	0.0001	-0.3944
C8	DG_4	0.2734	0.0001	0.2681	0.0000	0.2705	0.0000	0.2717	0.0002	0.2768	0.0000	0.2744	0.0000	0.2731
N9	DG_4	-0.2616	0.0000	-0.2630	0.0000	-0.2626	0.0000	-0.2643	0.0001	-0.2598	0.0001	-0.2611	0.0000	-0.2618
C4	DG_4	0.2199	0.0003	0.2195	0.0003	0.2188	0.0002	0.2128	0.0003	0.2203	0.0001	0.2200	0.0001	0.2184
N3	DG_4	-0.3987	0.0002	-0.4009	-0.0001	-0.4025	-0.0002	-0.4106	0.0010	-0.3958	0.0003	-0.3973	0.0001	-0.4011

C2	DG_4	0.3674	0.0002	0.3693	0.0005	0.3672	0.0006	0.3630	0.0001	0.3679	0.0000	0.3679	0.0000	0.3672
N1	DG_4	-0.0965	0.0011	-0.0948	0.0026	-0.0991	0.0049	-0.1018	0.0001	-0.0977	0.0000	-0.0967	0.0000	-0.0959
		-0.3207	0.0047	-0.3304	0.0050	-0.3475	0.0074	-0.3704	0.0034	-0.3136	0.0011	-0.3144	0.0006	-0.3120
P8	DA_5	0.5356	0.0000	0.5348	0.0000	0.5355	0.0000	0.5360	0.0000	0.5370	0.0000	0.5358	0.0000	0.5366
PO2	DA_5	-0.4150	0.0000	-0.4163	0.0000	-0.4151	0.0000	-0.4139	0.0000	-0.4119	0.0000	-0.4147	0.0000	-0.4147
PO1	DA_5	-0.0123	0.0000	-0.0141	0.0000	-0.0124	0.0000	-0.0130	0.0000	-0.0110	0.0000	-0.0121	0.0000	-0.0124
O5'	DA_5	-0.2397	0.0000	-0.2401	0.0000	-0.2397	0.0000	-0.2409	0.0000	-0.2391	0.0000	-0.2396	0.0000	-0.2405
O3'	DA_5	-0.1026	0.0001	-0.1034	0.0000	-0.1028	0.0000	-0.1018	0.0000	-0.1006	0.0000	-0.1023	0.0000	-0.1022
C1'	DA_5	0.2627	0.0002	0.2645	0.0000	0.2620	0.0000	0.2642	0.0000	0.2628	0.0000	0.2630	0.0000	0.2641
C2'	DA_5	0.0663	0.0001	0.0637	0.0000	0.0652	0.0000	0.0688	0.0000	0.0682	0.0000	0.0665	0.0000	0.0677
C3'	DA_5	0.1209	0.0000	0.1184	0.0000	0.1202	0.0000	0.1217	0.0000	0.1223	0.0000	0.1208	0.0000	0.1219
C4'	DA_5	0.1388	0.0006	0.1399	0.0000	0.1384	0.0000	0.1398	0.0000	0.1385	0.0000	0.1388	0.0000	0.1397
C5'	DA_5	0.2042	0.0004	0.2041	0.0000	0.2036	0.0000	0.2045	0.0000	0.2044	0.0000	0.2041	0.0000	0.2047
O4'	DA_5	-0.2413	0.0000	-0.2406	0.0000	-0.2411	0.0000	-0.2417	0.0000	-0.2425	0.0000	-0.2412	0.0000	-0.2409
C6	DA_5	0.2566	0.0008	0.2557	0.0002	0.2552	0.0003	0.2552	0.0000	0.2563	0.0000	0.2568	0.0000	0.2571
C5	DA_5	0.0657	0.0003	0.0640	0.0003	0.0637	0.0001	0.0650	0.0000	0.0648	0.0000	0.0654	0.0000	0.0657
N7	DA_5	-0.3866	0.0001	-0.3894	-0.0001	-0.3886	0.0000	-0.3857	0.0000	-0.3871	0.0000	-0.3862	0.0000	-0.3867
C8	DA_5	0.3161	0.0008	0.3113	0.0006	0.3124	0.0002	0.3137	0.0000	0.3185	0.0000	0.3165	0.0000	0.3153
N9	DA_5	-0.2534	0.0001	-0.2538	0.0002	-0.2544	0.0000	-0.2564	0.0000	-0.2526	0.0000	-0.2534	0.0000	-0.2537
C4	DA_5	0.2226	0.0001	0.2226	0.0001	0.2214	0.0000	0.2209	0.0000	0.2229	0.0000	0.2226	0.0000	0.2224
N3	DA_5	-0.3813	0.0007	-0.3806	0.0006	-0.3848	0.0001	-0.3855	0.0000	-0.3792	0.0000	-0.3809	0.0000	-0.3817
C2	DA_5	0.2894	0.0012	0.2926	0.0022	0.2890	0.0016	0.2883	0.0000	0.2908	0.0000	0.2895	0.0000	0.2902
N1	DA_5	-0.3539	0.0002	-0.3528	0.0008	-0.3526	0.0004	-0.3522	0.0000	-0.3542	0.0000	-0.3539	0.0000	-0.3534
N6	DA_5	0.0319	0.0005	0.0264	0.0015	0.0214	0.0003	0.0177	0.0000	0.0317	0.0000	0.0326	0.0000	0.0345

Table S5 continued														
GrA^{O}G-DNA														
Atom number	Nucleotide	Neutral	^{NE}Vertical Anion		^{EQ}Vertical Anion		Adiabatic Anion		^{NE}Vertical Cation		^{EQ}Vertical Cation		Adiabatic C ation	
		Q-CM5	S-H	Q-CM5	S-H	Q-CM5	S-H	Q-CM5	S-H	Q-CM5	S-H	Q-CM5	S-H	Q-CM5
O5'	DT_1	-0.052	0.001	-0.055	0.001	-0.057	0.000	-0.056	0.000	-0.052	0.000	-0.053	0.000	-0.052
O3'	DT_1	-0.241	0.002	-0.244	0.000	-0.242	0.000	-0.242	0.000	-0.239	0.000	-0.240	0.000	-0.240
C1'	DT_1	0.239	0.005	0.238	0.008	0.232	0.006	0.236	0.000	0.239	0.000	0.239	0.000	0.240
C2'	DT_1	0.050	0.006	0.052	0.010	0.042	0.010	0.045	0.000	0.049	0.000	0.050	0.000	0.050
C3'	DT_1	0.140	0.002	0.133	0.001	0.134	0.001	0.134	0.000	0.141	0.000	0.140	0.000	0.141
C4'	DT_1	0.137	0.001	0.130	0.001	0.131	0.000	0.132	0.000	0.139	0.000	0.137	0.000	0.137
C5'	DT_1	0.174	0.001	0.165	0.002	0.166	0.000	0.169	0.000	0.176	0.000	0.175	0.000	0.175
O4'	DT_1	-0.274	0.001	-0.279	0.003	-0.281	0.000	-0.281	0.000	-0.274	0.000	-0.274	0.000	-0.274
C4	DT_1	0.256	0.008	0.253	0.055	0.226	0.002	0.254	0.000	0.256	0.000	0.257	0.000	0.256
N3	DT_1	-0.110	0.001	-0.108	0.010	-0.119	0.002	-0.107	0.000	-0.110	0.000	-0.107	0.000	-0.108
C2	DT_1	0.360	0.003	0.363	0.007	0.358	0.004	0.367	0.000	0.362	0.000	0.362	0.000	0.361
O2	DT_1	-0.362	0.001	-0.365	0.002	-0.379	0.000	-0.367	0.000	-0.364	0.000	-0.365	0.000	-0.361
N1	DT_1	-0.276	0.002	-0.274	0.010	-0.279	0.002	-0.271	0.000	-0.273	0.000	-0.272	0.000	-0.274
C6	DT_1	0.171	0.010	0.162	0.084	0.121	0.000	0.171	0.000	0.175	0.000	0.173	0.000	0.173
C5	DT_1	-0.042	0.006	-0.047	0.026	-0.065	0.003	-0.048	0.000	-0.042	0.000	-0.042	0.000	-0.042
O4	DT_1	-0.364	0.009	-0.375	0.045	-0.408	0.005	-0.378	0.000	-0.368	0.000	-0.366	0.000	-0.363
CM5	DT_1	0.058	0.002	0.042	0.005	0.024	0.001	0.048	0.000	0.060	0.000	0.059	0.000	0.060
P1	DC_2	0.535	0.007	0.528	0.000	0.534	0.000	0.532	0.000	0.537	0.000	0.537	0.000	0.536
PO2	DC_2	-0.411	0.005	-0.423	0.000	-0.416	0.000	-0.418	0.000	-0.410	0.000	-0.414	0.000	-0.412
PO1	DC_2	0.008	0.010	-0.002	0.000	0.006	0.000	0.004	0.000	0.009	0.000	0.009	0.000	0.009
O5'	DC_2	-0.236	0.003	-0.233	0.001	-0.235	0.000	-0.234	0.000	-0.236	0.000	-0.235	0.000	-0.234
O3'	DC_2	-0.238	0.003	-0.243	0.000	-0.240	0.000	-0.240	0.000	-0.237	0.000	-0.237	0.000	-0.236

C1'	DC_2	0.260	0.011	0.247	0.011	0.242	0.016	0.236	0.000	0.260	0.000	0.261	0.000	0.265
C2'	DC_2	0.051	0.015	0.038	0.013	0.034	0.014	0.032	0.000	0.051	0.000	0.052	0.000	0.038
C3'	DC_2	0.139	0.006	0.128	0.002	0.131	0.001	0.131	0.000	0.141	0.000	0.139	0.000	0.136
C4'	DC_2	0.146	0.002	0.136	0.000	0.137	0.001	0.132	0.000	0.147	0.000	0.147	0.000	0.147
C5'	DC_2	0.204	0.005	0.190	0.001	0.196	0.001	0.194	0.000	0.206	0.000	0.204	0.000	0.204
O4'	DC_2	-0.234	0.005	-0.237	0.005	-0.236	0.011	-0.241	0.000	-0.234	0.000	-0.234	0.000	-0.231
N4	DC_2	0.077	0.056	0.009	0.060	-0.006	0.068	-0.087	0.000	0.077	0.001	0.090	0.000	0.100
C4	DC_2	0.273	0.099	0.224	0.105	0.221	0.189	0.167	0.000	0.274	0.000	0.275	0.000	0.276
N3	DC_2	-0.363	0.036	-0.391	0.047	-0.392	0.086	-0.401	0.000	-0.366	0.000	-0.366	0.000	-0.362
C2	DC_2	0.355	0.011	0.344	0.011	0.343	0.010	0.336	0.000	0.356	0.000	0.356	0.000	0.359
O2	DC_2	-0.355	0.000	-0.373	-0.001	-0.383	-0.005	-0.402	0.000	-0.356	0.000	-0.356	0.000	-0.354
N1	DC_2	-0.262	0.043	-0.284	0.052	-0.284	0.099	-0.290	0.000	-0.260	0.000	-0.257	0.000	-0.259
C6	DC_2	0.216	0.168	0.114	0.192	0.099	0.409	0.052	0.000	0.224	0.000	0.222	0.000	0.226
C5	DC_2	0.014	0.033	-0.035	0.037	-0.050	0.030	-0.064	0.000	0.018	0.000	0.019	0.000	0.025
P2	DT_3	0.539	0.011	0.528	0.000	0.538	0.000	0.537	0.000	0.541	0.000	0.541	0.000	0.539
PO2	DT_3	-0.408	0.007	-0.423	0.000	-0.413	0.000	-0.414	0.000	-0.406	0.000	-0.410	0.000	-0.412
PO1	DT_3	-0.008	0.016	-0.024	0.000	-0.011	0.000	-0.012	0.000	-0.008	0.000	-0.008	0.000	-0.011
O5'	DT_3	-0.237	0.002	-0.236	0.000	-0.236	0.000	-0.238	0.000	-0.238	0.000	-0.236	0.000	-0.241
O3'	DT_3	-0.240	0.002	-0.243	0.000	-0.240	0.000	-0.240	0.000	-0.239	0.000	-0.239	0.000	-0.239
C1'	DT_3	0.258	0.004	0.253	0.002	0.254	0.000	0.253	0.000	0.257	0.000	0.259	0.000	0.259
C2'	DT_3	0.047	0.008	0.039	0.003	0.042	0.000	0.046	0.000	0.048	0.000	0.048	0.000	0.049
C3'	DT_3	0.138	0.004	0.132	0.000	0.135	0.000	0.138	0.000	0.140	0.000	0.138	0.000	0.140
C4'	DT_3	0.143	0.001	0.136	0.000	0.140	0.000	0.142	0.000	0.144	0.000	0.144	0.000	0.147
C5'	DT_3	0.207	0.004	0.197	0.000	0.203	0.000	0.202	0.000	0.209	0.000	0.207	0.000	0.208
O4'	DT_3	-0.250	0.002	-0.249	0.001	-0.252	0.000	-0.259	0.000	-0.252	0.000	-0.251	0.000	-0.245
O4	DT_3	-0.344	0.021	-0.360	0.021	-0.366	0.002	-0.345	0.000	-0.348	0.000	-0.345	0.000	-0.325
C4	DT_3	0.256	0.023	0.247	0.028	0.246	0.002	0.260	0.000	0.257	0.000	0.257	0.000	0.258

N3	DT_3	-0.106	0.008	-0.113	0.011	-0.112	0.001	-0.105	0.000	-0.110	0.000	-0.107	0.000	-0.100
C2	DT_3	0.361	0.003	0.360	0.002	0.360	0.000	0.362	0.000	0.361	0.001	0.361	0.000	0.365
O2	DT_3	-0.348	0.000	-0.362	-0.001	-0.362	0.000	-0.360	0.001	-0.351	0.002	-0.349	0.002	-0.343
N1	DT_3	-0.272	0.006	-0.273	0.009	-0.274	0.000	-0.269	0.000	-0.270	0.000	-0.268	0.000	-0.269
C6	DT_3	0.190	0.040	0.164	0.051	0.158	0.002	0.181	0.000	0.198	0.000	0.194	0.000	0.199
C5	DT_3	-0.039	0.015	-0.045	0.012	-0.045	0.001	-0.033	0.000	-0.038	0.000	-0.039	0.000	-0.038
CM5	DT_3	0.047	0.017	0.022	0.009	0.020	0.012	0.029	0.000	0.054	0.000	0.052	0.000	0.063
P3	DC_4	0.536	0.007	0.529	0.000	0.536	0.000	0.537	0.000	0.538	0.000	0.538	0.000	0.538
PO2	DC_4	-0.411	0.005	-0.423	0.000	-0.414	0.000	-0.413	0.000	-0.409	0.000	-0.413	0.000	-0.411
PO1	DC_4	0.008	0.013	-0.004	0.000	0.008	0.000	0.008	0.000	0.010	0.000	0.009	0.000	0.009
O5'	DC_4	-0.236	0.002	-0.236	0.000	-0.235	0.000	-0.236	0.000	-0.236	0.000	-0.235	0.000	-0.235
O3'	DC_4	-0.239	0.001	-0.240	0.000	-0.239	0.000	-0.237	0.000	-0.238	0.000	-0.238	0.000	-0.238
C1'	DC_4	0.260	0.002	0.256	0.000	0.258	0.000	0.257	0.000	0.258	0.000	0.260	0.000	0.260
C2'	DC_4	0.047	0.003	0.041	0.001	0.045	0.000	0.046	0.000	0.048	0.000	0.048	0.000	0.044
C3'	DC_4	0.138	0.002	0.134	0.000	0.137	0.000	0.139	0.000	0.141	0.000	0.138	0.000	0.138
C4'	DC_4	0.145	0.001	0.141	0.000	0.144	0.000	0.146	0.000	0.145	0.000	0.145	0.000	0.146
C5'	DC_4	0.203	0.003	0.196	0.000	0.202	0.000	0.204	0.000	0.205	0.000	0.204	0.000	0.205
O4'	DC_4	-0.235	0.001	-0.233	0.000	-0.235	0.000	-0.231	0.000	-0.236	0.000	-0.236	0.000	-0.235
N4	DC_4	0.077	0.009	0.065	0.001	0.072	0.000	0.071	0.000	0.081	0.000	0.078	0.000	0.085
C4	DC_4	0.270	0.016	0.264	0.003	0.270	0.000	0.272	0.000	0.271	0.000	0.270	0.000	0.271
N3	DC_4	-0.365	0.008	-0.371	0.003	-0.365	0.000	-0.362	0.000	-0.368	0.000	-0.365	0.000	-0.366
C2	DC_4	0.354	0.002	0.352	0.000	0.354	0.000	0.355	0.000	0.354	0.000	0.354	0.000	0.353
O2	DC_4	-0.354	0.000	-0.363	0.000	-0.359	0.000	-0.353	0.000	-0.358	0.000	-0.356	0.000	-0.351
N1	DC_4	-0.263	0.009	-0.266	0.003	-0.262	0.000	-0.261	0.000	-0.261	0.000	-0.260	0.000	-0.263
C6	DC_4	0.216	0.033	0.196	0.009	0.211	0.000	0.211	0.000	0.223	0.000	0.217	0.000	0.215
C5	DC_4	0.013	0.011	0.003	0.001	0.009	0.000	0.015	0.000	0.019	0.000	0.013	0.000	0.019

P4	DT_5	0.539	0.003	0.536	0.000	0.540	0.000	0.544	0.000	0.541	0.000	0.541	0.000	0.542
PO2	DT_5	-0.409	0.002	-0.416	0.000	-0.411	0.000	-0.406	0.000	-0.407	0.000	-0.411	0.000	-0.408
PO1	DT_5	-0.010	0.004	-0.014	0.000	-0.010	0.000	-0.008	0.000	-0.008	0.000	-0.010	0.000	-0.009
O5'	DT_5	-0.237	0.000	-0.236	0.000	-0.236	0.000	-0.234	0.000	-0.236	0.000	-0.236	0.000	-0.235
O3'	DT_5	-0.105	0.000	-0.108	0.000	-0.105	0.000	-0.102	0.000	-0.102	0.000	-0.105	0.000	-0.102
C1'	DT_5	0.261	0.000	0.259	0.000	0.260	0.000	0.261	0.000	0.259	0.000	0.261	0.000	0.262
C2'	DT_5	0.068	0.001	0.064	0.000	0.068	0.000	0.070	0.000	0.071	0.000	0.069	0.000	0.072
C3'	DT_5	0.117	0.001	0.115	0.000	0.117	0.000	0.115	0.000	0.120	0.000	0.117	0.000	0.116
C4'	DT_5	0.131	0.000	0.129	0.000	0.131	0.000	0.129	0.000	0.130	0.000	0.131	0.000	0.131
C5'	DT_5	0.205	0.001	0.202	0.000	0.204	0.000	0.200	0.000	0.206	0.000	0.205	0.000	0.204
O4'	DT_5	-0.252	0.000	-0.251	0.000	-0.252	0.000	-0.258	0.000	-0.254	0.000	-0.253	0.000	-0.256
O4	DT_5	-0.347	0.003	-0.351	0.000	-0.351	0.000	-0.353	0.000	-0.348	0.000	-0.351	0.000	-0.348
C4	DT_5	0.260	0.004	0.260	0.001	0.261	0.000	0.261	0.000	0.261	0.000	0.261	0.000	0.260
N3	DT_5	-0.103	0.002	-0.103	0.001	-0.101	0.000	-0.103	0.000	-0.105	0.000	-0.100	0.000	-0.100
C2	DT_5	0.367	0.000	0.368	0.000	0.369	0.000	0.365	0.000	0.368	0.000	0.369	0.000	0.369
O2	DT_5	-0.370	0.000	-0.378	0.000	-0.375	0.000	-0.380	0.001	-0.374	0.000	-0.374	0.000	-0.367
N1	DT_5	-0.270	0.001	-0.269	0.001	-0.268	0.000	-0.270	0.000	-0.268	0.000	-0.266	0.000	-0.269
C6	DT_5	0.192	0.006	0.186	0.002	0.191	0.000	0.186	0.000	0.197	0.000	0.193	0.000	0.192
C5	DT_5	-0.038	0.002	-0.037	0.000	-0.038	0.000	-0.038	0.000	-0.037	0.000	-0.038	0.000	-0.038
C6	DT_5	0.048	0.005	0.042	0.000	0.046	0.000	0.047	0.000	0.055	0.000	0.048	0.000	0.055
O5'	DA_1	-0.049	0.000	-0.048	0.000	-0.049	0.000	-0.049	0.000	-0.048	0.000	-0.050	0.000	-0.050
O3'	DA_1	-0.239	0.000	-0.240	0.000	-0.239	0.000	-0.238	0.000	-0.237	0.000	-0.238	0.000	-0.239
C1'	DA_1	0.240	0.000	0.240	0.000	0.240	0.000	0.240	0.000	0.240	0.000	0.240	0.000	0.240
C2'	DA_1	0.045	0.000	0.046	0.000	0.045	0.000	0.046	0.000	0.041	0.000	0.045	0.000	0.044
C3'	DA_1	0.139	0.000	0.138	0.000	0.139	0.000	0.140	0.000	0.143	0.000	0.139	0.000	0.140
C4'	DA_1	0.139	0.000	0.137	0.000	0.139	0.000	0.139	0.000	0.143	0.000	0.139	0.000	0.139
C5'	DA_1	0.175	0.000	0.173	0.000	0.175	0.000	0.175	0.000	0.180	0.000	0.175	0.000	0.175

O4'	DA_1	-0.275	0.000	-0.276	0.000	-0.275	0.000	-0.275	0.000	-0.272	0.000	-0.275	0.000	-0.275
N6	DA_1	0.026	0.001	0.028	0.000	0.026	0.000	0.025	0.004	0.038	0.000	0.027	0.000	0.034
C6	DA_1	0.253	0.000	0.253	0.000	0.253	0.000	0.253	0.001	0.253	0.000	0.252	0.000	0.253
C5	DA_1	0.059	0.000	0.059	0.000	0.059	0.000	0.059	0.008	0.061	0.000	0.059	0.000	0.059
N7	DA_1	-0.407	0.000	-0.407	0.000	-0.408	0.000	-0.408	0.001	-0.403	0.000	-0.408	0.000	-0.408
C8	DA_1	0.279	0.000	0.278	0.000	0.279	0.000	0.280	0.007	0.290	0.000	0.280	0.000	0.279
N9	DA_1	-0.264	0.000	-0.264	0.000	-0.263	0.000	-0.263	0.001	-0.263	0.000	-0.262	0.000	-0.263
C4	DA_1	0.219	0.000	0.219	0.000	0.220	0.000	0.220	0.004	0.220	0.000	0.220	0.000	0.219
N3	DA_1	-0.380	0.000	-0.382	0.000	-0.380	0.000	-0.377	0.002	-0.377	0.000	-0.380	0.000	-0.379
C2	DA_1	0.287	0.000	0.286	0.000	0.287	0.000	0.287	0.001	0.290	0.000	0.287	0.000	0.289
N3	DA_1	-0.360	0.000	-0.360	0.000	-0.360	0.000	-0.359	0.001	-0.359	0.000	-0.360	0.000	-0.360
P5	DG_2	0.536	0.000	0.536	0.000	0.536	0.000	0.537	0.000	0.539	0.000	0.537	0.000	0.536
PO2	DG_2	-0.412	0.000	-0.415	0.000	-0.414	0.000	-0.413	0.000	-0.408	0.000	-0.415	0.000	-0.413
PO1	DG_2	-0.008	0.000	-0.008	0.000	-0.008	0.000	-0.009	0.000	-0.008	0.000	-0.008	0.000	-0.009
O5'	DG_2	-0.241	0.000	-0.240	0.000	-0.241	0.000	-0.241	0.000	-0.243	0.000	-0.241	0.000	-0.242
O3'	DG_2	-0.247	0.000	-0.248	0.000	-0.247	0.000	-0.240	0.000	-0.243	0.000	-0.246	0.000	-0.240
C1'	DG_2	0.259	0.000	0.258	0.000	0.259	0.000	0.264	0.000	0.264	0.000	0.260	0.000	0.261
C2'	DG_2	0.045	0.000	0.046	0.000	0.045	0.000	0.050	0.001	0.044	0.000	0.046	0.000	0.053
C3'	DG_2	0.141	0.000	0.139	0.000	0.140	0.000	0.140	0.000	0.145	0.000	0.141	0.000	0.144
C4'	DG_2	0.148	0.000	0.146	0.000	0.147	0.000	0.147	0.000	0.153	0.000	0.148	0.000	0.148
C5'	DG_2	0.209	0.000	0.207	0.000	0.209	0.000	0.209	0.000	0.216	0.000	0.210	0.000	0.209
O4'	DG_2	-0.228	0.000	-0.228	0.000	-0.228	0.000	-0.231	0.000	-0.227	0.000	-0.228	0.000	-0.229
N2	DG_2	0.046	0.000	0.044	0.000	0.045	0.000	0.043	0.067	0.104	0.000	0.048	0.000	0.040
O6	DG_2	-0.375	0.000	-0.371	0.000	-0.377	0.000	-0.372	0.023	-0.348	0.000	-0.375	0.000	-0.373
C6	DG_2	0.247	0.000	0.248	0.000	0.247	0.000	0.247	0.011	0.260	0.000	0.247	0.000	0.245
C5	DG_2	0.036	0.000	0.036	0.000	0.036	0.000	0.038	0.086	0.075	0.001	0.037	0.000	0.036
N7	DG_2	-0.396	0.000	-0.396	0.000	-0.398	0.000	-0.395	0.020	-0.375	0.000	-0.397	0.000	-0.396

C8	DG_2	0.286	0.000	0.282	0.000	0.286	0.000	0.288	0.056	0.336	0.000	0.289	0.000	0.290
N9	DG_2	-0.257	0.000	-0.257	0.000	-0.256	0.000	-0.257	0.004	-0.249	0.000	-0.255	0.000	-0.257
C4	DG_2	0.218	0.000	0.218	0.000	0.219	0.000	0.220	0.033	0.236	0.000	0.219	0.000	0.220
N3	DG_2	-0.400	0.000	-0.404	0.000	-0.401	0.000	-0.399	0.081	-0.358	0.001	-0.399	0.000	-0.399
C2	DG_2	0.371	0.000	0.371	0.000	0.371	0.000	0.371	0.016	0.384	0.000	0.370	0.000	0.370
N1	DG_2	-0.095	0.000	-0.094	0.000	-0.095	0.000	-0.093	-0.002	-0.086	0.000	-0.095	0.000	-0.097
P6	rA_3	0.540	0.000	0.541	0.000	0.541	0.000	0.540	0.000	0.544	0.000	0.542	0.000	0.540
PO2	rA_3	-0.418	0.000	-0.421	0.000	-0.419	0.000	-0.407	0.000	-0.412	0.000	-0.419	0.000	-0.406
PO1	rA_3	0.013	0.000	0.015	0.000	0.014	0.000	0.017	0.000	0.013	0.000	0.014	0.000	0.019
O5'	rA_3	-0.229	0.000	-0.227	0.000	-0.228	0.000	-0.228	0.000	-0.230	0.000	-0.228	0.000	-0.230
O3'	rA_3	-0.229	0.000	-0.228	0.000	-0.228	0.000	-0.230	0.000	-0.228	0.000	-0.227	0.000	-0.230
C1'	rA_3	0.244	0.000	0.244	0.000	0.244	0.000	0.243	0.000	0.245	0.000	0.246	0.000	0.242
C2'	rA_3	0.119	0.000	0.118	0.000	0.119	0.000	0.114	0.000	0.121	0.000	0.122	0.000	0.120
O2'	rA_3	-0.134	0.000	-0.135	0.000	-0.134	0.000	-0.142	0.002	-0.127	0.004	-0.123	0.004	-0.124
C3'	rA_3	0.167	0.000	0.165	0.000	0.167	0.000	0.164	0.000	0.173	0.000	0.171	0.000	0.166
C4'	rA_3	0.156	0.000	0.154	0.000	0.156	0.000	0.155	0.000	0.161	0.000	0.160	0.000	0.159
C5'	rA_3	0.228	0.000	0.226	0.000	0.228	0.000	0.208	0.000	0.233	0.000	0.231	0.000	0.210
O4'	rA_3	-0.261	0.000	-0.261	0.000	-0.261	0.000	-0.263	0.000	-0.262	0.000	-0.258	0.000	-0.258
N6	rA_3	0.062	0.002	0.064	0.001	0.058	0.001	0.047	0.037	0.093	0.009	0.084	0.004	0.062
C6	rA_3	0.259	0.000	0.259	0.000	0.259	0.000	0.258	0.007	0.261	0.002	0.255	0.001	0.253
C5	rA_3	0.063	0.000	0.062	0.000	0.063	0.000	0.063	0.026	0.072	0.015	0.064	0.018	0.064
N7	rA_3	-0.377	0.000	-0.380	0.000	-0.380	0.000	-0.379	0.009	-0.372	0.003	-0.370	0.000	-0.374
C8	rA_3	0.301	0.000	0.294	0.000	0.299	0.000	0.306	0.023	0.325	0.016	0.320	0.016	0.322
N9	rA_3	-0.260	0.000	-0.260	0.000	-0.259	0.000	-0.258	0.003	-0.257	0.000	-0.258	0.001	-0.260
C4	rA_3	0.219	0.000	0.219	0.000	0.219	0.000	0.221	0.011	0.224	0.007	0.220	0.011	0.220
N3	rA_3	-0.383	0.000	-0.387	0.000	-0.385	0.000	-0.381	0.029	-0.365	0.014	-0.371	0.005	-0.376
C2	rA_3	0.293	0.000	0.294	0.000	0.291	0.000	0.293	0.008	0.309	0.001	0.304	0.003	0.305

N1	rA_3	-0.355	0.000	-0.351	0.000	-0.353	0.000	-0.354	0.009	-0.347	0.008	-0.353	0.000	-0.354
P7	$^{O}G_4$	0.518	0.000	0.517	0.000	0.518	0.000	0.517	0.000	0.520	0.000	0.519	0.000	0.519
PO2	$^{O}G_4$	-0.024	0.000	-0.026	0.000	-0.024	0.000	-0.024	0.000	-0.020	0.000	-0.022	0.000	-0.020
PO1	$^{O}G_4$	-0.457	0.000	-0.459	0.000	-0.457	0.000	-0.457	0.000	-0.453	0.000	-0.456	0.000	-0.454
O5'	$^{O}G_4$	-0.241	0.000	-0.241	0.000	-0.241	0.000	-0.241	0.000	-0.240	0.000	-0.240	0.000	-0.242
O3'	$^{O}G_4$	-0.235	0.000	-0.235	0.000	-0.234	0.000	-0.237	0.000	-0.233	0.000	-0.232	0.000	-0.236
C1'	$^{O}G_4$	0.253	0.000	0.254	0.000	0.253	0.000	0.254	0.000	0.257	-0.001	0.266	-0.001	0.266
C2'	$^{O}G_4$	0.030	0.000	0.028	0.000	0.029	0.000	0.026	0.000	0.035	0.000	0.040	0.000	0.039
C3'	$^{O}G_4$	0.150	0.000	0.148	0.000	0.150	0.000	0.134	0.000	0.153	0.000	0.155	0.000	0.143
C4'	$^{O}G_4$	0.142	0.000	0.141	0.000	0.141	0.000	0.138	0.000	0.146	0.000	0.149	0.000	0.146
C5'	$^{O}G_4$	0.219	0.000	0.217	0.000	0.218	0.000	0.214	0.000	0.223	0.000	0.225	0.000	0.220
O4'	$^{O}G_4$	-0.242	0.000	-0.242	0.000	-0.243	0.000	-0.240	0.000	-0.245	0.000	-0.240	0.000	-0.238
N2	$^{O}G_4$	0.054	0.003	0.050	0.001	0.047	0.001	0.028	0.047	0.104	0.112	0.164	0.102	0.155
O6	$^{O}G_4$	-0.365	0.002	-0.366	0.000	-0.370	0.000	-0.379	0.049	-0.331	0.113	-0.277	0.098	-0.291
C6	$^{O}G_4$	0.244	0.005	0.243	0.001	0.244	0.001	0.239	0.014	0.258	0.024	0.273	0.034	0.269
C5	$^{O}G_4$	0.053	0.001	0.051	0.000	0.051	0.000	0.049	0.098	0.094	0.243	0.149	0.267	0.146
N7	$^{O}G_4$	-0.039	0.000	-0.042	0.000	-0.041	0.000	-0.042	0.039	-0.007	0.108	0.048	0.128	0.041
O8	$^{O}G_4$	-0.367	0.000	-0.376	0.000	-0.371	0.000	-0.376	0.036	-0.338	0.099	-0.288	0.084	-0.289
C8	$^{O}G_4$	0.356	0.000	0.354	0.000	0.355	0.000	0.353	0.014	0.370	0.023	0.388	0.031	0.385
N9	$^{O}G_4$	-0.260	0.000	-0.260	0.000	-0.259	0.000	-0.259	0.003	-0.253	-0.002	-0.246	-0.007	-0.252
C4	$^{O}G_4$	0.229	0.004	0.226	0.001	0.228	0.000	0.223	0.030	0.248	0.055	0.268	0.058	0.270
N3	$^{O}G_4$	-0.386	0.000	-0.392	0.000	-0.391	0.000	-0.398	0.047	-0.358	0.104	-0.317	0.103	-0.331
C2	$^{O}G_4$	0.374	0.002	0.373	0.001	0.373	0.001	0.368	0.014	0.387	0.021	0.403	0.024	0.399
N1	$^{O}G_4$	-0.088	0.007	-0.088	0.004	-0.088	0.005	-0.095	-0.003	-0.079	-0.015	-0.072	-0.011	-0.081
P8	DA_5	0.532	0.000	0.531	0.000	0.533	0.000	0.535	0.000	0.536	0.000	0.535	0.000	0.538
PO2	DA_5	-0.426	0.000	-0.430	0.000	-0.428	0.000	-0.418	0.000	-0.421	0.000	-0.425	0.000	-0.413

PO1	DA_5	-0.032	0.000	-0.033	0.000	-0.032	0.000	-0.016	0.000	-0.031	0.000	-0.030	0.000	-0.011
	DA_5													
O5'	DA_5	-0.248	0.000	-0.247	0.000	-0.247	0.000	-0.244	0.000	-0.247	0.000	-0.247	0.000	-0.242
O3'	DA_5	-0.102	0.000	-0.104	0.000	-0.102	0.000	-0.103	0.000	-0.098	0.000	-0.100	0.000	-0.100
C1'	DA_5	0.265	0.000	0.266	0.000	0.264	0.000	0.263	0.000	0.264	0.000	0.269	0.000	0.267
C2'	DA_5	0.068	0.000	0.066	0.000	0.067	0.000	0.068	0.000	0.073	0.000	0.074	0.000	0.073
C3'	DA_5	0.123	0.000	0.119	0.000	0.122	0.000	0.122	0.000	0.126	0.000	0.125	0.000	0.124
C4'	DA_5	0.141	0.000	0.140	0.000	0.140	0.000	0.140	0.000	0.141	0.000	0.143	0.000	0.142
C5'	DA_5	0.202	0.000	0.199	0.000	0.201	0.000	0.201	0.000	0.204	0.000	0.204	0.000	0.205
O4'	DA_5	-0.237	0.000	-0.236	0.000	-0.237	0.000	-0.241	0.000	-0.240	0.000	-0.238	0.000	-0.240
C6	DA_5	0.259	0.006	0.255	0.000	0.257	0.000	0.256	0.001	0.259	0.001	0.258	0.001	0.257
C5	DA_5	0.065	0.001	0.062	0.000	0.063	0.000	0.064	0.003	0.064	0.007	0.063	0.005	0.061
N7	DA_5	-0.385	0.001	-0.391	0.000	-0.389	0.000	-0.387	0.001	-0.386	0.001	-0.383	0.002	-0.382
C8	DA_5	0.314	0.007	0.302	0.001	0.311	0.000	0.316	0.001	0.319	0.003	0.322	0.002	0.322
N9	DA_5	-0.255	0.001	-0.256	0.000	-0.255	0.000	-0.256	0.001	-0.254	0.001	-0.253	0.002	-0.253
C4	DA_5	0.221	0.001	0.221	0.000	0.221	0.000	0.222	0.002	0.221	0.004	0.221	0.004	0.221
N3	DA_5	-0.381	0.005	-0.385	0.001	-0.385	0.000	-0.382	0.001	-0.380	0.001	-0.377	0.001	-0.379
C2	DA_5	0.291	0.007	0.294	0.003	0.291	0.002	0.292	0.001	0.297	0.003	0.300	0.003	0.299
N1	DA_5	-0.353	0.001	-0.351	0.001	-0.351	0.001	-0.353	0.000	-0.351	-0.001	-0.350	0.000	-0.353
N6	DA_5	0.043	0.003	0.033	0.001	0.032	0.000	0.027	0.004	0.051	0.010	0.064	0.005	0.063

Table S5 continued

rA^{O}G-DNA

Atom number	Nucleotide	Neutral	^{NE}Vertical Anion		^{EQ}Vertical Anion		Adiabatic Anion		^{NE}Vertical Cation		^{EQ}Vertical Cation		Adiabatic C ation	
		Q-CM5	S-H	Q-CM5	S-H	Q-CM5	S-H	Q-CM5	S-H	Q-CM5	S-H	Q-CM5	S-H	Q-CM5
O5'	DT$_1$	-0.053068	0.002302	-0.056015	0.001813	-0.060538	0.000018	-0.056653	0	-0.053441	0	-0.053076	0	-0.052636
O3'	DT$_1$	-0.240497	0.003321	-0.245274	0.000038	-0.242798	0.00002	-0.24161	0.000004	-0.237278	0.000004	-0.239236	0.000002	-0.240755
C1'	DT$_1$	0.236405	0.003863	0.235462	0.011585	0.227393	0.004962	0.232593	0.000067	0.236582	0.000043	0.236491	0.000056	0.240027
C2'	DT$_1$	0.047362	0.007607	0.046904	0.01449	0.036269	0.008697	0.044043	0.000156	0.040068	0.000131	0.046961	0.000113	0.048109
C3'	DT$_1$	0.139059	0.003967	0.132468	0.00198	0.131334	0.00139	0.134092	0.000009	0.14082	0.000006	0.139769	0.000003	0.140209
C4'	DT$_1$	0.136243	0.00128	0.129805	0.001099	0.128495	0.000125	0.130846	0.000014	0.140769	0.000007	0.137314	0.000003	0.136741
C5'	DT$_1$	0.174145	0.001466	0.164715	0.003179	0.162832	0.000014	0.16887	0.000004	0.179554	0.000001	0.17543	0.000001	0.174181
O4'	DT$_1$	-0.275023	0.000892	-0.279992	0.003664	-0.285021	0.000107	-0.282031	0.000061	-0.273027	0.000025	-0.275152	0.000003	-0.273425
C4	DT$_1$	0.255309	0.003136	0.254297	0.077343	0.213774	0.002479	0.251414	0.000044	0.256173	0.000007	0.256435	-0.000012	0.255254
N3	DT$_1$	-0.108132	0.001641	-0.107463	0.01517	-0.123169	0.002518	-0.107088	0.000151	-0.107671	0.000086	-0.105713	-0.000008	-0.109929
C2	DT$_1$	0.360553	0.002016	0.3624	0.006562	0.355737	0.00284	0.366029	0.000155	0.361282	0.000085	0.361517	0.000076	0.359856
O2	DT$_1$	-0.361929	0.000325	-0.363583	-0.00023	-0.381834	-0.00010	-0.365932	0.000196	-0.36413	0.000058	-0.365548	0.000018	-0.36151
N1	DT$_1$	-0.277521	0.001106	-0.275619	0.016271	-0.283384	0.001084	-0.273714	0.00099	-0.274806	0.000598	-0.273332	0.000231	-0.276984
C6	DT$_1$	0.169558	0.003052	0.166291	0.113944	0.100744	0.00004	0.165538	0.000096	0.170976	0.000008	0.171164	0.000021	0.170768
C5	DT$_1$	-0.042422	0.002808	-0.045545	0.037173	-0.074106	0.002311	-0.047684	0.000282	-0.043065	0.000138	-0.042411	0.000024	-0.042352
O4	DT$_1$	-0.36325	0.003882	-0.371162	0.061275	-0.419727	0.004122	-0.382297	-0.000019	-0.361479	-0.00002	-0.364037	-0.000015	-0.362907
CM5	DT$_1$	0.056196	0.002151	0.044436	0.00753	0.010663	0.000726	0.041038	0.000067	0.060323	0.000025	0.058513	0.000006	0.057795
P1	DC$_2$	0.534575	0.015681	0.520422	0.000407	0.532471	0.000103	0.531771	0.000004	0.536676	-0.00000	0.536473	-0.00000	0.535188
PO2	DC$_2$	-0.412695	0.009851	-0.426333	0.000235	-0.417921	0.000032	-0.419152	0.000001	-0.407767	0.000006	-0.414343	0.000003	-0.410881
PO1	DC$_2$	0.007149	0.023831	-0.014552	0.00038	0.004099	0.000081	0.004081	0.000012	0.005639	0.000009	0.007946	0.000009	0.008373
O5'	DC$_2$	-0.235523	0.004593	-0.234588	0.000356	-0.235053	0.000144	-0.23516	0.000011	-0.238912	0.000009	-0.234991	0.000004	-0.234602
O3'	DC$_2$	-0.239253	0.006535	-0.246493	0.000012	-0.241513	-0.00001	-0.239667	0.000003	-0.235163	0.000006	-0.236793	0.000011	-0.238668

C1'	DC_2	0.253923	0.00815	0.24327	0.010906	0.236523	0.015907	0.232139	0.000497	0.258278	0.000261	0.257752	0.000152	0.256444
C2'	DC_2	0.029414	0.013978	0.020398	0.009311	0.017551	0.012084	0.019012	0.00124	0.022419	0.00093	0.030478	0.000779	0.03065
C3'	DC_2	0.134296	0.011065	0.122477	0.000942	0.125867	0.000955	0.128822	0.000083	0.138044	0.000065	0.138003	0.000054	0.133722
C4'	DC_2	0.142788	0.003146	0.132557	0.000422	0.132365	0.00054	0.131503	0.00008	0.148742	0.00005	0.14616	0.000016	0.143568
C5'	DC_2	0.201499	0.007604	0.185973	0.001345	0.192835	0.001162	0.191682	0.000052	0.20883	0.000032	0.204351	0.000009	0.203009
O4'	DC_2	-0.240471	0.004257	-0.245113	0.007001	-0.24358	0.011779	-0.246543	0.000505	-0.237552	0.000364	-0.239138	0.000142	-0.241388
N4	DC_2	0.052644	0.032277	0.012366	0.04753	-0.015695	0.069263	-0.106727	0.003149	0.059904	0.001397	0.061117	0.000103	0.066993
C4	DC_2	0.267263	0.061825	0.235557	0.105074	0.220455	0.196754	0.166309	0.001062	0.259507	0.000463	0.260106	0.001632	0.264408
N3	DC_2	-0.369426	0.02534	-0.389573	0.054625	-0.401552	0.082219	-0.40408	0.001503	-0.37081	0.001573	-0.370256	0.001743	-0.369505
C2	DC_2	0.353275	0.005701	0.345895	0.00815	0.34164	0.011548	0.331811	0.001304	0.35277	0.000814	0.352839	0.001379	0.35118
O2	DC_2	-0.35802	-0.000498	-0.37085	-0.002412	-0.38983	-0.003467	-0.406839	0.001283	-0.347228	0.000669	-0.349739	0.000346	-0.352623
N1	DC_2	-0.264242	0.029912	-0.279367	0.059028	-0.28668	0.101312	-0.291545	0.009011	-0.259429	0.007065	-0.259199	0.002423	-0.263487
C6	DC_2	0.203378	0.112457	0.13616	0.206326	0.08828	0.380678	0.05084	0.010315	0.213427	0.009343	0.213992	0.009503	0.212101
C5	DC_2	0.005643	0.029526	-0.030351	0.041428	-0.060018	0.027108	-0.062029	0.011911	0.007208	0.00757	0.010092	0.001787	0.006822
P2	$^{O}G_3$	0.536913	0.028662	0.512165	0.000127	0.535301	0.00008	0.536677	0.000002	0.541256	-0.00002	0.540076	-0.00002	0.537477
PO2	$^{O}G_3$	-0.410769	0.014239	-0.431869	0.000037	-0.415298	0.000026	-0.413728	0.000004	-0.402644	0.00002	-0.410834	0.000024	-0.409337
PO1	$^{O}G_3$	-0.011198	0.064916	-0.069683	0.000108	-0.013824	0.000081	-0.015899	0.000002	-0.010472	0	-0.009011	0	-0.010392
O5'	$^{O}G_3$	-0.234863	0.007126	-0.235907	0.000012	-0.234474	-0.000004	-0.232479	0.000034	-0.237611	0.000015	-0.233916	-0.000003	-0.236853
O3'	$^{O}G_3$	-0.239814	0.006968	-0.245492	0.000012	-0.240312	0.000014	-0.240032	0.000162	-0.235025	0.000105	-0.236072	0.000002	-0.238995
C1'	$^{O}G_3$	0.249798	0.001348	0.24505	0.000878	0.246542	0.000152	0.243534	0.00005	0.260265	-0.000887	0.263679	-0.000896	0.256079
C2'	$^{O}G_3$	0.026641	0.011721	0.01551	0.000563	0.023667	0.000337	0.02409	0.001024	0.03078	0.000321	0.0365	-0.00033	0.030323
C3'	$^{O}G_3$	0.133392	0.01799	0.119635	0.000107	0.131224	0.000027	0.133296	0.000331	0.139281	0.000145	0.140477	-0.00005	0.136005
C4'	$^{O}G_3$	0.141122	0.00231	0.133192	0.000069	0.137696	0.000042	0.13847	0.000035	0.149644	-0.000031	0.149612	-0.000065	0.146072
C5'	$^{O}G_3$	0.209399	0.007491	0.194391	0.000085	0.205907	0.000065	0.203684	0.000141	0.220281	0.000079	0.216728	0.000009	0.212785
O4'	$^{O}G_3$	-0.247968	0.000411	-0.247918	0.000486	-0.246867	0.000344	-0.264937	0.000936	-0.245319	0.000463	-0.24347	-0.000102	-0.242016
N2	$^{O}G_3$	0.11953	0.062646	0.058018	0.00238	0.10337	0.005067	0.093968	0.074126	0.222011	0.084748	0.233184	0.076761	0.221765
O6	$^{O}G_3$	-0.368394	0.003695	-0.378053	0.00391	-0.378891	0.004135	-0.368948	0.098203	-0.282199	0.116721	-0.274813	0.092039	-0.305399

C6	$^{O}G_3$	0.243501	0.006821	0.240159	0.005359	0.242898	0.004124	0.248182	0.038842	0.275223	0.033592	0.2801	0.051678	0.269167
C5	$^{O}G_3$	0.055318	0.001884	0.054336	0.001436	0.059939	0.003348	0.058658	0.204987	0.13908	0.232166	0.144711	0.170785	0.106148
N7	$^{O}G_3$	-0.105417	0.001926	-0.108276	0.001163	-0.104754	0.001305	-0.107734	0.145966	-0.02694	0.159147	-0.021409	0.200703	-0.325459
C8	$^{O}G_3$	0.344984	0.00285	0.342722	0.00289	0.343852	0.002069	0.342253	0.025987	0.376173	0.017281	0.377951	0.046451	0.34558
N9	$^{O}G_3$	-0.263341	0.003611	-0.263777	0.002289	-0.262131	0.000502	-0.26322	0.014669	-0.24299	0.00643	-0.242546	0.001942	-0.257571
C4	$^{O}G_3$	0.230287	0.014976	0.225297	0.011549	0.229185	0.005259	0.231062	0.069246	0.272779	0.066699	0.277359	0.091692	0.266549
N3	$^{O}G_3$	-0.365224	0.010704	-0.368826	0.008732	-0.369634	0.010079	-0.369133	0.060801	-0.318084	0.066716	-0.314778	0.043061	-0.342574
C2	$^{O}G_3$	0.381025	0.011242	0.374007	0.004911	0.375912	0.006294	0.377699	0.031979	0.413442	0.027961	0.419807	0.041462	0.4096
N1	$^{O}G_3$	-0.021212	0.010961	-0.033884	0.002986	-0.030324	0.002281	-0.024363	-0.006084	0.010841	-0.014302	0.018893	-0.009892	0.000796
O8	$^{O}G_3$	-0.370684	0.000864	-0.379578	0.000508	-0.380313	0.001296	-0.387215	0.131714	-0.269231	0.151414	-0.26627	0.147232	-0.293702
P3	DC_4	0.537386	0.025359	0.516289	-0.000005	0.537386	0.000004	0.536435	0.000012	0.541512	-0.000017	0.540731	-0.000042	0.538503
PO2	DC_4	-0.409345	0.014549	-0.428025	0.000016	-0.411602	-0.000002	-0.412965	0.000002	-0.399936	0.000012	-0.408577	0.000033	-0.408154
PO1	DC_4	0.014258	0.05679	-0.035139	0.000016	0.013629	0.00001	0.012163	0.000007	0.014713	0.000003	0.016906	0.000005	0.014204
O5'	DC_4	-0.236847	0.005367	-0.239876	0.000003	-0.236451	0	-0.237659	0.000003	-0.237614	0.000001	-0.235874	-0.000002	-0.237695
O3'	DC_4	-0.232447	0.001403	-0.233938	0.000012	-0.232505	0.000001	-0.232443	0.000032	-0.230055	0.00003	-0.230838	0.000112	-0.232598
C1'	DC_4	0.260798	0.000519	0.258689	0.000139	0.259991	0.000023	0.258258	0.001195	0.26101	0.001095	0.26414	0.002662	0.259336
C2'	DC_4	0.048107	0.00051	0.042184	0.0001	0.047138	0.000008	0.04244	0.000218	0.055061	0.000195	0.052772	0.000991	0.049161
C3'	DC_4	0.131849	0.002846	0.13004	0.000105	0.131588	0.000001	0.132667	0.000051	0.132988	0.000043	0.133299	0.000158	0.1341
C4'	DC_4	0.148333	0.001306	0.14333	-0.000014	0.147458	-0.000001	0.149494	0.00003	0.15303	0.000035	0.151844	0.000037	0.15182
C5'	DC_4	0.200172	0.005887	0.189344	0.000008	0.19917	0	0.200587	0.000007	0.206686	0.000002	0.203907	0.000006	0.203793
O4'	DC_4	-0.232325	0.000211	-0.230728	0.000026	-0.232441	0.000002	-0.227101	0.000104	-0.238126	0.00009	-0.234602	0.000193	-0.22841
N4	DC_4	0.070148	0.007341	0.06198	0.000174	0.066823	0.000012	0.067213	0.005903	0.075613	0.005204	0.083539	0.000858	0.089748
C4	DC_4	0.270678	0.007554	0.267343	0.000802	0.270355	0.000005	0.269444	0.000958	0.263593	0.000701	0.265476	0.000624	0.270808
N3	DC_4	-0.362491	0.002835	-0.366012	0.000633	-0.362631	0.000015	-0.364045	0.00476	-0.360525	0.003628	-0.361484	0.001376	-0.36529
C2	DC_4	0.354189	0.000679	0.353646	0.000133	0.354633	0.000019	0.352342	0.00085	0.353329	0.000473	0.352689	0.001658	0.351963
O2	DC_4	-0.356414	0.000093	-0.362272	-0.00007	-0.359281	0.000045	-0.360163	0.003117	-0.344733	0.002596	-0.34991	0.000996	-0.345296
N1	DC_4	-0.265994	0.002837	-0.265652	0.001271	-0.263977	0.00006	-0.263981	0.00017	-0.26561	-0.000137	-0.264863	0.005598	-0.26688

C6	DC$_4$	0.201024	0.011083	0.195873	0.003753	0.199466	0.000089	0.203534	0.001773	0.206568	0.001595	0.204486	0.003916	0.207363
C5	DC$_4$	0.012444	0.012433	0.00389	0.000008	0.00989	0.000102	0.011173	0.00124	0.005252	0.000961	0.014487	0.001692	0.020182
P4	DT$_5$	0.54568	0.004971	0.541463	-0.000017	0.546169	0.000006	0.546011	0.000003	0.548567	-0.000009	0.547776	-0.000008	0.546591
PO2	DT$_5$	-0.401885	0.003541	-0.40923	0.000035	-0.403361	-0.000006	-0.40475	0.000004	-0.397311	0.000013	-0.403453	0.000019	-0.400838
PO1	DT$_5$	0.036899	0.010407	0.029077	-0.000001	0.036885	0	0.03756	0	0.037359	0	0.038003	0.000004	0.038269
O5'	DT$_5$	-0.224812	0.00082	-0.22474	-0.000004	-0.224409	0	-0.225175	0.000003	-0.224342	0.000002	-0.223912	0.000006	-0.224803
O3'	DT$_5$	-0.097534	0.00075	-0.100886	0.000002	-0.097918	0	-0.09718	0.000001	-0.094095	0.000005	-0.097466	0.000003	-0.097024
C1'	DT$_5$	0.260799	0.000046	0.259917	0.000002	0.26063	0.000001	0.261484	0.000009	0.261396	0	0.261137	-0.000001	0.261054
C2'	DT$_5$	0.07488	0.000266	0.070527	0.000007	0.074621	0	0.075976	0.000009	0.078188	0.000005	0.075453	0.000005	0.075499
C3'	DT$_5$	0.117044	0.000668	0.116124	0.000002	0.117167	0.000001	0.117697	0.000001	0.11753	-0.000006	0.117519	-0.000004	0.117548
C4'	DT$_5$	0.132685	0.000196	0.130501	0	0.132671	0	0.132536	0	0.134575	-0.000002	0.133111	-0.000001	0.132468
C5'	DT$_5$	0.202615	0.001258	0.199108	0.000001	0.202453	0	0.2022	0	0.204491	-0.000002	0.203191	-0.000001	0.203166
O4'	DT$_5$	-0.25189	0.00003	-0.251215	0.000002	-0.252424	0	-0.250843	0.000002	-0.254362	0.000003	-0.252686	0.000001	-0.253258
O4	DT$_5$	-0.354184	0.000538	-0.354275	-0.000102	-0.355538	-0.000023	-0.353194	0.00002	-0.358658	0.000035	-0.357109	0.000032	-0.355387
C4	DT$_5$	0.257378	0.000485	0.258494	-0.000009	0.257635	-0.000002	0.258175	0.000025	0.257625	0.000003	0.257896	0	0.256639
N3	DT$_5$	-0.104512	0.000158	-0.10442	0.000063	-0.103243	0.000011	-0.101586	0.000017	-0.102515	-0.000006	-0.101739	-0.000008	-0.106874
C2	DT$_5$	0.362806	0.000081	0.363176	0.000008	0.362988	0.000001	0.36639	0.000047	0.365092	-0.00002	0.364427	-0.000014	0.362961
O2	DT$_5$	-0.378593	0.000009	-0.383789	0.000001	-0.380652	-0.000002	-0.37527	0.000014	-0.376503	0.000043	-0.381461	0.000036	-0.374844
N1	DT$_5$	-0.272965	0.000054	-0.272075	0.000111	-0.271238	0.000016	-0.26995	0.000117	-0.270047	0.000043	-0.268737	0.000002	-0.272186
C6	DT$_5$	0.170516	0.000287	0.168927	0.000139	0.170406	0.000015	0.174088	0.000085	0.173729	0.000033	0.172074	0.000025	0.171275
C5	DT$_5$	-0.042858	0.000464	-0.042103	-0.000126	-0.042962	-0.000015	-0.043199	0.000089	-0.045627	0.000018	-0.044226	0.000023	-0.04402
CM5	DT$_5$	0.015623	0.006279	0.011328	0.000048	0.015057	-0.000002	0.02013	0.000041	0.015883	0.00002	0.017823	0.00013	0.018789
O5'	DA$_1$	-0.049896	0.000024	-0.049113	0	-0.050061	0	-0.049821	0	-0.050258	-0.000001	-0.050116	-0.000001	-0.049842
O3'	DA$_1$	-0.238942	0.000001	-0.239521	0	-0.238674	-0.000001	-0.238928	0	-0.237532	-0.000002	-0.238232	0	-0.238737
C1'	DA$_1$	0.241352	0.000011	0.241288	0.000001	0.241353	0.000001	0.240682	0.000005	0.242223	-0.000004	0.241563	-0.000001	0.240626
C2'	DA$_1$	0.043911	0.000021	0.044451	0	0.044219	-0.000001	0.044772	0.000011	0.04206	0.000002	0.043962	0	0.042994

C3'	DA$_1$	0.138971	0.000005	0.137916	0	0.138925	0	0.138453	0	0.139843	-0.000003	0.138992	-0.000001	0.139724
C4'	DA$_1$	0.138868	0.000001	0.137162	0	0.138819	0	0.138807	0	0.14104	-0.000003	0.139114	-0.000001	0.139136
C5'	DA$_1$	0.174669	0.00001	0.172743	0.000001	0.17473	0	0.174846	0.000002	0.176629	0.000001	0.175054	0.000001	0.174215
O4'	DA$_1$	-0.274523	0	-0.275892	0	-0.275196	0	-0.275194	0.000003	-0.273967	0.000001	-0.275155	-0.000001	-0.27445
N6	DA$_1$	0.03191	0.001056	0.03352	0.000013	0.031932	-0.000002	0.032173	0.000231	0.028837	-0.000005	0.032697	-0.000007	0.036755
C6	DA$_1$	0.253133	0.000103	0.253366	0.000002	0.252866	0.000001	0.252664	0.000064	0.251896	-0.00001	0.252791	-0.000005	0.25441
C5	DA$_1$	0.05846	0.00004	0.057953	0.000001	0.058349	0.000001	0.057711	0.000419	0.057629	0.000015	0.058291	0.000004	0.059643
N7	DA$_1$	-0.406313	0.000061	-0.406522	0.000001	-0.407323	0.000002	-0.407576	0.000071	-0.408653	0.00002	-0.40808	0.000005	-0.404472
C8	DA$_1$	0.279069	0.00005	0.277598	0.000002	0.279266	0	0.278564	0.000363	0.282943	0.000019	0.27998	0.000015	0.281113
N9	DA$_1$	-0.264411	0.000008	-0.264007	-0.000002	-0.263646	-0.000003	-0.263646	0.000049	-0.261889	-0.00004	-0.26235	-0.000023	-0.264427
C4	DA$_1$	0.218154	0.000019	0.218049	0	0.218342	-0.000001	0.217863	0.00018	0.21973	-0.000017	0.218838	-0.000008	0.218108
N3	DA$_1$	-0.382294	0.000035	-0.383254	0	-0.382485	0.000002	-0.383461	0.000165	-0.378137	0.000027	-0.38186	0.000011	-0.380652
C2	DA$_1$	0.287767	0.000037	0.286926	0.000001	0.287643	0.000001	0.287895	0.00005	0.289462	-0.000015	0.288118	-0.000003	0.288971
N1	DA$_1$	-0.359635	0.000032	-0.359543	0	-0.359603	0.000002	-0.360486	0.000032	-0.361159	0.000029	-0.359844	0.000017	-0.359934
P5	DG$_2$	0.536014	0.000008	0.536088	0.000006	0.536615	0.000008	0.537022	0	0.537864	-0.000015	0.537412	0.000001	0.536383
PO2	DG$_2$	-0.411477	0.000001	-0.41393	-0.000005	-0.412764	-0.000007	-0.412249	0	-0.409868	0.000017	-0.41393	-0.000001	-0.411606
PO1	DG$_2$	-0.009525	0.000034	-0.008935	0	-0.009389	-0.000001	-0.008997	0	-0.009875	0.000001	-0.009379	0	-0.007925
O5'	DG$_2$	-0.24041	0.000003	-0.239264	0	-0.239775	0	-0.239872	0	-0.240657	-0.000001	-0.239407	0	-0.240712
O3'	DG$_2$	-0.223477	0.000001	-0.224282	0	-0.223244	-0.000001	-0.222337	-0.000001	-0.221857	0	-0.222529	0.000001	-0.222221
C1'	DG$_2$	0.261042	0.000013	0.260341	0	0.260649	0.000001	0.262973	-0.000013	0.262451	-0.000003	0.261603	-0.000001	0.262046
C2'	DG$_2$	0.032995	0.000074	0.033398	-0.000001	0.03313	0.000001	0.035764	0.00003	0.031631	0.000004	0.03324	0.000002	0.045691
C3'	DG$_2$	0.1486	0.000006	0.147318	0	0.148395	0.000001	0.14882	0	0.149842	-0.000001	0.148758	-0.000001	0.154658
C4'	DG$_2$	0.152747	0.000003	0.151145	0	0.152388	0	0.152304	0	0.154137	0	0.152825	0	0.156538
C5'	DG$_2$	0.212547	0.000001	0.210042	0	0.212215	0	0.212044	0	0.215143	0	0.212731	0.000001	0.215081
O4'	DG$_2$	-0.228137	0.000004	-0.228608	0	-0.22851	0	-0.228062	0.000001	-0.227283	-0.000001	-0.228181	0	-0.223805
N2	DG$_2$	0.042761	0.00017	0.040626	0.000048	0.041896	0.000004	0.036749	0.00242	0.048401	0.000007	0.045671	0.000006	0.049722
O6	DG$_2$	-0.370217	0.000382	-0.367397	-0.000006	-0.371159	0.00002	-0.375418	0.002815	-0.376222	0.000391	-0.372058	0.000215	-0.373972

C6	DG$_2$	0.24707	0.001243	0.247411	0.000016	0.247298	0.000017	0.247099	0.00021	0.246463	-0.000026	0.247393	0.000021	0.244656
C5	DG$_2$	0.038972	0.00016	0.038671	0.000018	0.03929	0.000012	0.038545	0.003958	0.039298	0.000095	0.039264	0.000018	0.037202
N7	DG$_2$	-0.391923	0.00027	-0.393058	-0.000003	-0.393388	0	-0.395348	0.000745	-0.393939	-0.000005	-0.393575	0.000005	-0.394087
C8	DG$_2$	0.288854	0.000332	0.285152	0.000026	0.288486	0.000016	0.288405	0.002649	0.299574	0.000081	0.292202	0.00002	0.290266
N9	DG$_2$	-0.258993	0.000056	-0.258462	-0.000004	-0.258154	-0.000003	-0.258054	0.000092	-0.254398	-0.000018	-0.256256	-0.000014	-0.258722
C4	DG$_2$	0.220716	0.000551	0.220175	0.000017	0.220996	0.000016	0.221097	0.001464	0.223979	0.000034	0.221917	0.000009	0.220463
N3	DG$_2$	-0.397619	0.000134	-0.400311	0.000013	-0.398542	0.00001	-0.399517	0.003988	-0.390331	0.000147	-0.396002	0.000028	-0.396215
C2	DG$_2$	0.370794	0.000176	0.371096	0.000021	0.370708	0.000003	0.370502	0.00059	0.371047	-0.000017	0.370452	-0.000004	0.368398
N1	DG$_2$	-0.094377	0.001037	-0.094042	0.000066	-0.093258	0.000008	-0.094193	-0.000152	-0.096927	0.000036	-0.093775	0.000038	-0.097584
P6	rA$_3$	0.54742	0.00001	0.547241	0	0.547864	0.000006	0.54827	0	0.549624	-0.000009	0.548828	-0.000001	0.549498
PO2	rA$_3$	-0.412937	0	-0.41599	0.000003	-0.414257	-0.000005	-0.413942	0	-0.410491	0.000007	-0.415009	-0.000001	-0.406206
PO1	rA$_3$	0.030935	0.000054	0.032594	0.000001	0.031293	0	0.031165	0	0.030128	-0.000001	0.031552	0.000001	0.0311
O5'	rA$_3$	-0.235187	0.000002	-0.235778	-0.000001	-0.235044	-0.000001	-0.234404	0	-0.233695	0.000001	-0.23454	0	-0.232544
O3'	rA$_3$	-0.220497	0.000017	-0.22029	0	-0.220137	-0.000001	-0.215483	0.000004	-0.220772	0.000001	-0.21974	0	-0.214124
C1'	rA$_3$	0.247863	0.000032	0.247803	0.00002	0.24727	0.000001	0.246023	0.000022	0.248145	-0.000003	0.24895	-0.000001	0.248983
C2'	rA$_3$	0.111958	0.000075	0.110708	0.000007	0.111829	0.000001	0.112498	0.000006	0.113262	0.000001	0.112595	0.000001	0.115111
O2'	rA$_3$	-0.166675	0.000144	-0.167177	0.00001	-0.167474	0.000001	-0.152292	0.00004	-0.165006	0.000001	-0.165829	0.000001	-0.144875
C3'	rA$_3$	0.15965	0.000027	0.157238	0.000001	0.159224	0	0.160161	0.000002	0.162468	-0.000001	0.160177	0.000001	0.160537
C4'	rA$_3$	0.152514	0.000013	0.151189	0.000003	0.152035	0	0.153457	0	0.153735	0	0.153023	0	0.157887
C5'	rA$_3$	0.215911	0.000006	0.213387	0.000001	0.215485	0	0.215714	0.000001	0.218391	0	0.216407	0	0.215783
O4'	rA$_3$	-0.247029	0.000011	-0.247315	0.000009	-0.247627	0.000001	-0.248793	0	-0.246874	0.000001	-0.246755	0.000004	-0.256283
N6	rA$_3$	0.048857	0.002432	0.048524	0.001299	0.04697	0.001402	0.059517	0.000557	0.050921	0.000343	0.057647	0.00056	0.123115
C6	rA$_3$	0.260788	0.004418	0.260141	0.000781	0.261345	0.000775	0.261479	-0.000101	0.25967	-0.000092	0.261419	0.00011	0.293873
C5	rA$_3$	0.066443	0.000341	0.065256	0.000087	0.065619	0.000033	0.064556	0.000384	0.069513	0.000028	0.06893	-0.00003	0.077016
N7	rA$_3$	-0.386852	0.000758	-0.388788	0.000042	-0.389128	0.000043	-0.388401	0.000076	-0.384118	-0.000004	-0.385218	-0.000011	-0.370837
C8	rA$_3$	0.307795	0.001063	0.301711	-0.000034	0.305424	0.000042	0.303805	0.000306	0.321201	0.000071	0.314334	0.000054	0.316488
N9	rA$_3$	-0.260157	0.000093	-0.260937	0.000129	-0.260248	0.000008	-0.260478	0.000007	-0.255556	0.000006	-0.256772	0.000024	-0.250353

C4	rA_3	0.225062	0.001252	0.223206	0.000328	0.223915	0.000126	0.222837	0.000213	0.229885	0.000118	0.22799	0.00014	0.237542
N3	rA_3	-0.373695	0.000522	-0.376782	-0.000009	-0.376616	-0.000008	-0.380256	0.00003	-0.363184	-0.00003	-0.367398	-0.000062	-0.344896
C2	rA_3	0.29514	0.00075	0.296123	0.000776	0.29351	0.000119	0.293072	0.000236	0.287887	-0.000026	0.296412	0.000105	0.358755
N1	rA_3	-0.350977	0.000825	-0.348835	0.000574	-0.349189	0.000188	-0.353103	-0.000298	-0.362587	-0.00061	-0.355934	0.002108	-0.058273
P7	DG_4	0.549124	0.000076	0.548499	0.000006	0.549005	0.000001	0.551829	0.000008	0.551223	0	0.549851	-0.000004	0.552556
PO2	DG_4	-0.00071	0.000108	-0.003904	0	-0.001201	-0.000001	-0.013736	-0.000001	0.003399	0.000002	-0.000022	0.000002	-0.011664
PO1	DG_4	-0.375204	0.000059	-0.37568	0.000004	-0.376287	0.000004	-0.372356	0.000005	-0.375553	-0.000002	-0.376858	0.000003	-0.371021
O5'	DG_4	-0.243318	0.000018	-0.244756	-0.000001	-0.243508	-0.000001	-0.240592	0.000005	-0.240969	0	-0.24271	-0.000001	-0.238749
O3'	DG_4	-0.238856	0.000016	-0.238913	0.000001	-0.238683	0	-0.240033	0	-0.238503	0	-0.238203	0	-0.240599
C1'	DG_4	0.228628	0.000237	0.228987	0.00003	0.228171	0.000008	0.243543	-0.000011	0.229282	-0.000002	0.229227	-0.000001	0.249838
C2'	DG_4	0.036114	0.000276	0.034384	0.000033	0.035899	0.000008	0.032506	0.000003	0.037875	0.000002	0.036783	0	0.033964
C3'	DG_4	0.129488	0.000055	0.12701	0.000003	0.12907	0	0.128185	-0.000001	0.131916	-0.000001	0.129794	0	0.130279
C4'	DG_4	0.137987	0.000028	0.136541	0.000002	0.137482	0.000001	0.132814	0.000002	0.139486	0	0.138384	0	0.134457
C5'	DG_4	0.196589	0.000116	0.196621	0.000023	0.1962	0	0.201028	0.00007	0.194849	0.000001	0.196858	0	0.205307
O4'	DG_4	-0.274372	0.000077	-0.274488	0.00001	-0.275598	0.000006	-0.272676	-0.000006	-0.276829	0	-0.275081	0	-0.266917
N2	DG_4	0.02624	0.001089	0.024417	0.000791	0.017663	0.00085	0.000961	0.005961	0.027548	0.001397	0.031016	0.001574	0.03791
O6	DG_4	-0.352086	0.001635	-0.35215	0.000275	-0.353983	0.000004	-0.371473	0.001471	-0.352725	0.000064	-0.353925	-0.000011	-0.358458
C6	DG_4	0.250268	0.004813	0.248708	0.001704	0.24851	0.000877	0.242481	0.000419	0.249478	0.000033	0.251272	0.000055	0.245854
C5	DG_4	0.045207	0.000722	0.043815	0.000054	0.044075	-0.000053	0.040883	0.003388	0.046572	0.000077	0.046157	0.000048	0.042441
N7	DG_4	-0.357032	0.000689	-0.359637	0.000199	-0.359387	0.000041	-0.368389	0.00087	-0.357641	0.000023	-0.358657	0.000007	-0.36726
C8	DG_4	0.27499	0.000519	0.269007	0.000143	0.271876	0.000011	0.270856	0.002149	0.284988	0.000048	0.278184	0.000028	0.280233
N9	DG_4	-0.264759	0.000257	-0.264867	0.000018	-0.264898	0.000025	-0.262864	0.000119	-0.260195	0.000007	-0.261689	0	-0.261824
C4	DG_4	0.217253	0.002425	0.215489	0.000675	0.215389	0.00026	0.214987	0.001113	0.219819	0.000024	0.219313	-0.000003	0.221706
N3	DG_4	-0.398887	0.00015	-0.4033	-0.000129	-0.403763	-0.000127	-0.407213	0.003261	-0.391134	0.000136	-0.395775	0.000089	-0.395348
C2	DG_4	0.367997	0.001102	0.367802	0.000555	0.36644	0.000514	0.360332	0.000451	0.365355	0.000106	0.367971	0.000155	0.369633
N1	DG_4	-0.091455	0.005009	-0.092332	0.003625	-0.09413	0.004663	-0.103463	0.000038	-0.100592	0.000147	-0.092872	0.000432	-0.091179

P8	DA_5	0.53464	0.000093	0.534248	0.000002	0.535176	-0.000007	0.534974	0	0.53693	-0.000005	0.536171	0	0.534816
PO2	DA_5	-0.413717	0.00004	-0.416947	-0.000003	-0.415258	0.000007	-0.415157	0	-0.412025	0.000005	-0.416298	0	-0.412449
PO1	DA_5	-0.008486	0.000267	-0.009917	0.000001	-0.008647	0	-0.011898	0	-0.006819	0	-0.008142	0	-0.010071
O5'	DA_5	-0.240349	0.000039	-0.239604	0	-0.239798	0.000001	-0.239893	0	-0.239345	-0.000001	-0.239218	0	-0.23953
O3'	DA_5	-0.099436	0.000025	-0.101474	0.000001	-0.100058	0	-0.102862	0.000001	-0.097016	0.000003	-0.09939	0	-0.101136
C1'	DA_5	0.26964	0.000133	0.270414	0.000021	0.268574	-0.000001	0.262203	0.000004	0.268504	-0.000003	0.269783	0	0.265786
C2'	DA_5	0.068771	0.000331	0.066356	0.000021	0.067827	0.000006	0.065789	0.000003	0.070622	0.000003	0.069154	0.000001	0.066851
C3'	DA_5	0.121784	0.000119	0.119318	0.000003	0.121465	0	0.120486	0.000001	0.12367	-0.000003	0.122047	0	0.120272
C4'	DA_5	0.14126	0.000038	0.140911	0.000002	0.140715	0.000001	0.137403	0	0.140619	-0.000002	0.141128	0	0.138009
C5'	DA_5	0.201594	0.000043	0.19998	0.000002	0.201118	-0.000001	0.202169	0	0.202052	0	0.201555	0	0.20203
O4'	DA_5	-0.234365	0.000033	-0.233594	0.000008	-0.234624	-0.000001	-0.24099	0.000004	-0.235384	0.000004	-0.234574	0.000001	-0.241819
C6	DA_5	0.256491	0.005039	0.253731	0.000204	0.254539	0.000307	0.254277	0.000015	0.25639	-0.000004	0.256411	-0.000004	0.25688
C5	DA_5	0.064373	0.001001	0.062121	0.000191	0.062659	0.00007	0.063099	0.000028	0.064791	0.000013	0.064565	0.000004	0.064617
N7	DA_5	-0.384022	0.00098	-0.388355	-0.000113	-0.387272	-0.000028	-0.388971	0.00006	-0.383062	0.000014	-0.385682	0.000004	-0.385713
C8	DA_5	0.315048	0.004958	0.305983	0.000464	0.311436	0.000236	0.314543	0.000012	0.32198	0.000001	0.317084	-0.000002	0.316988
N9	DA_5	-0.255728	0.000442	-0.256128	0.00015	-0.255837	-0.000015	-0.254417	0.000043	-0.253275	-0.000003	-0.253231	0.000002	-0.254772
C4	DA_5	0.221269	0.000501	0.220321	0.000063	0.219974	0.000051	0.221182	0.000029	0.221982	-0.000004	0.221972	-0.000001	0.221038
N3	DA_5	-0.382738	0.002977	-0.385343	0.000328	-0.387064	0.00007	-0.384599	0.000026	-0.381276	0.000042	-0.382162	0.000029	-0.381834
C2	DA_5	0.289847	0.004914	0.291266	0.002153	0.288462	0.001774	0.287936	0.000083	0.28583	0.000033	0.28947	0.000025	0.288261
N1	DA_5	-0.354894	0.000501	-0.35379	0.000627	-0.353399	0.000495	-0.355183	0.000035	-0.358057	0.000011	-0.355497	0.000004	-0.354776
N6	DA_5	0.038225	0.002786	0.030959	0.001789	0.026502	0.000257	0.027943	0.000026	0.042422	0.000002	0.04039	0	0.039404

Table S5 continued

^{O}GA-DNA

Atom number	Nucleotide	Neutral	^{NE}Vertical Anion		^{EQ}Vertical Anion		Adiabatic Anion		^{NE}Vertical Cation		^{EQ}Vertical Cation		Adiabatic Cation	
		Q-CM5	S-H	Q-CM5	S-H	Q-CM5	S-H	Q-CM5	S-H	Q-CM5	S-H	Q-CM5	S-H	Q-CM5
O5'	DT$_1$	-0.052489	0.000001	-0.052155	0.000001	-0.053319	0	-0.052028	0	-0.052082	-0.000001	-0.052169	0	-0.051434
O3'	DT$_1$	-0.240882	0.000001	-0.242488	0.000002	-0.240656	0.000002	-0.241169	0	-0.240195	0	-0.240508	0.000001	-0.240668
C1'	DT$_1$	0.239294	0.000004	0.239103	0.000017	0.238091	0	0.241888	0	0.239563	-0.000001	0.239518	-0.000001	0.242822
C2'	DT$_1$	0.049226	0.000004	0.052199	0.000029	0.050164	0.000001	0.048537	0	0.048589	0.000002	0.049537	0	0.048917
C3'	DT$_1$	0.139433	0.000001	0.138276	0.000004	0.1393	-0.000001	0.14128	0	0.141226	-0.000001	0.139738	-0.000001	0.141926
C4'	DT$_1$	0.136873	0.000001	0.134299	0.000003	0.136064	0	0.137148	0	0.138968	0	0.137308	0	0.137934
O4	DT$_1$	-0.375352	0.000013	-0.373314	0.000175	-0.374211	0.000002	-0.354505	0.000018	-0.37764	0.000052	-0.375386	0.000025	-0.356277
C5'	DT$_1$	0.173943	0.000001	0.170684	0.000005	0.174078	0	0.173576	0	0.176133	0.000001	0.174417	0	0.174857
O4'	DT$_1$	-0.275848	0.000001	-0.277073	0.000005	-0.275657	0	-0.27394	0	-0.27496	0	-0.275222	0	-0.273118
C4	DT$_1$	0.254084	0.000012	0.254344	0.00015	0.25244	0	0.259719	0.000003	0.25381	-0.000009	0.254301	-0.000003	0.259155
N3	DT$_1$	-0.100588	0.000014	-0.101043	0	-0.101642	-0.000003	-0.109051	-0.000004	-0.102968	-0.000041	-0.100322	-0.000015	-0.109992
C2	DT$_1$	0.358227	0.000012	0.357898	0.000049	0.356459	0	0.362592	0.000002	0.35788	-0.000005	0.358069	-0.000003	0.362027
O2	DT$_1$	-0.378433	0.000011	-0.376752	0.000028	-0.376083	0.000002	-0.369138	0.000006	-0.376222	0.000017	-0.378146	0.000012	-0.366988
N1	DT$_1$	-0.265056	0.000006	-0.266807	0.000019	-0.266195	0.000001	-0.274576	0.000002	-0.264752	-0.000009	-0.264509	-0.000004	-0.273852
C6	DT$_1$	0.170404	0.000009	0.168167	0.000134	0.169164	0.000004	0.171284	-0.000003	0.175019	0	0.172138	0.000002	0.173256
C5	DT$_1$	-0.043266	0.000002	-0.042811	-0.000008	-0.043215	-0.000003	-0.043539	0.000003	-0.04286	0.000002	-0.042479	-0.000001	-0.042955
CM5	DT$_1$	0.054087	0.000022	0.052239	0.000055	0.053086	0	0.056367	0.000001	0.056554	0	0.055925	0	0.058859
P1	DA$_2$	0.537265	0.00001	0.53625	0.000003	0.536693	-0.000002	0.536148	0	0.538092	-0.000008	0.537626	-0.000003	0.537435
PO2	DA$_2$	-0.412087	0.000005	-0.414398	0.000004	-0.41346	0.000001	-0.410015	0	-0.408947	0.000004	-0.412421	0.000001	-0.408716
PO1	DA$_2$	0.008246	0.00001	0.008426	0.000008	0.007723	0	0.008224	0	0.008854	0.000002	0.008481	0	0.009018

O5'	DA_2	-0.230926	0.000003	-0.229443	0.000002	-0.231271	0.000001	-0.23212	0	-0.231806	0	-0.230837	0	-0.231397
O3'	DA_2	-0.237219	0.000068	-0.239695	0.000043	-0.238188	0.000003	-0.23849	0	-0.236649	0.000001	-0.236751	0.000001	-0.237497
C1'	DA_2	0.261918	0.000297	0.260821	0.000939	0.258339	0.000009	0.268004	-0.000001	0.262467	-0.000005	0.26354	-0.000004	0.267659
C2'	DA_2	0.038789	0.001773	0.042341	0.005946	0.037816	0.000035	0.051449	0.000002	0.039443	0.000005	0.039668	0.000003	0.052099
C3'	DA_2	0.132216	0.000146	0.12988	0.000471	0.129629	-0.000002	0.137026	0.000001	0.134329	0	0.132825	-0.000001	0.137211
C4'	DA_2	0.143067	0.000057	0.140148	0.000103	0.140502	0.000001	0.144694	0.000001	0.144309	0	0.143975	-0.000001	0.146309
C5'	DA_2	0.20383	0.000028	0.199422	0.000044	0.202534	0	0.205439	0	0.206217	0.000001	0.204425	0.000001	0.207062
O4'	DA_2	-0.242602	0.000024	-0.244773	0.000005	-0.244357	0.000001	-0.239636	0.000002	-0.24231	0.000004	-0.242168	0.000005	-0.24255
N6	DA_2	0.059253	0.000588	0.05653	0.001603	0.05063	0.000021	0.027411	0.00051	0.058438	0.000473	0.066702	0.000445	0.108592
C6	DA_2	0.263285	0.000207	0.265947	0.000799	0.265999	0.000028	0.263345	-0.00004	0.262083	-0.000105	0.263406	0.000028	0.291219
C5	DA_2	0.062277	0.000369	0.065082	0.000976	0.0673	0.000008	0.068758	0.000057	0.063824	0.000035	0.063776	-0.000043	0.076299
N7	DA_2	-0.367342	0.000363	-0.365732	0.002008	-0.368618	0.000032	-0.374719	-0.000013	-0.364889	-0.000015	-0.364621	0.000001	-0.35443
C8	DA_2	0.311683	0.000268	0.306679	0.00069	0.305806	0.000006	0.310784	0.000079	0.323329	0.000105	0.317975	0.000017	0.337327
N9	DA_2	-0.249004	0.000712	-0.250367	0.001814	-0.249172	0.000007	-0.253757	0.000013	-0.245882	0.000008	-0.247265	0.000008	-0.24329
C4	DA_2	0.216979	0.000638	0.217808	0.001639	0.218522	0.000027	0.222653	0.000102	0.220587	0.000148	0.219318	0.000061	0.232838
N3	DA_2	-0.386171	0.00042	-0.38841	0.000455	-0.391632	0.000056	-0.38543	-0.000011	-0.375888	-0.000017	-0.378455	-0.000042	-0.344853
C2	DA_2	0.29209	0.000848	0.289763	0.001084	0.285863	0.000057	0.293129	0.000189	0.288729	0.000059	0.298117	0.000028	0.362564
N1	DA_2	-0.348595	0.000026	-0.347502	0.000182	-0.346732	0.000013	-0.350213	0.000006	-0.357421	-0.000507	-0.353137	0.001785	-0.065176
P2	DT_3	0.538321	0.000268	0.535849	0.000176	0.536447	-0.000006	0.538606	0	0.539496	-0.000009	0.538826	-0.000017	0.539061
PO2	DT_3	-0.412986	0.000148	-0.418374	0.000087	-0.416293	0.000008	-0.40935	0	-0.40928	0.000006	-0.413319	0.000022	-0.408705
PO1	DT_3	-0.011772	0.000386	-0.011737	0.000387	-0.013806	0.000004	-0.007952	0	-0.011037	0.000002	-0.011343	-0.000002	-0.007981
O5'	DT_3	-0.240996	0.000168	-0.238536	0.000242	-0.241299	0.000005	-0.238069	0	-0.241584	0	-0.240764	-0.000003	-0.240934
O3'	DT_3	-0.238678	0.000499	-0.24332	0.000013	-0.240691	0.000034	-0.241331	0	-0.238115	0	-0.238235	0	-0.238385
C1'	DT_3	0.251925	0.005418	0.246464	0.009378	0.237301	0.002512	0.254151	0	0.250805	-0.000003	0.252645	-0.000002	0.257859
C2'	DT_3	0.049202	0.006243	0.050979	0.014346	0.032625	0.00937	0.045022	0.000001	0.050471	0.000003	0.050187	0.000002	0.04399
C3'	DT_3	0.139699	0.000564	0.13315	0.001083	0.132604	0.000874	0.134811	0	0.142591	0	0.140339	-0.000001	0.137346

C4'	DT_3	0.146438	0.000849	0.139869	0.000804	0.138292	0.000126	0.140628	0	0.146824	-0.000001	0.14716	-0.000001	0.14753
C5'	DT_3	0.209908	0.000488	0.201224	0.000652	0.203641	0.000044	0.203706	0	0.212109	0	0.210408	0	0.21018
O4'	DT_3	-0.242734	0.002096	-0.247331	0.004672	-0.24747	-0.000074	-0.259905	0	-0.242682	0.000002	-0.242382	0.000001	-0.239654
O4	DT_3	-0.35489	0.039107	-0.382177	0.083938	-0.422402	0.0001	-0.336948	-0.000024	-0.35268	-0.000004	-0.352766	0.000019	-0.346553
C4	DT_3	0.255133	0.047493	0.234504	0.123533	0.196097	0.001622	0.259812	0.000017	0.254569	0.000007	0.254329	-0.000007	0.251388
N3	DT_3	-0.098186	0.007981	-0.100821	0.029878	-0.120721	0.003874	-0.100697	-0.000027	-0.103551	-0.000019	-0.100605	0.000025	-0.11012
C2	DT_3	0.35791	0.010862	0.355633	0.00706	0.347979	0.002199	0.367642	0.000015	0.356512	0.000003	0.356792	0.000019	0.357139
O2	DT_3	-0.358371	0.002701	-0.370906	-0.001952	-0.380259	-0.000132	-0.369163	0.000044	-0.360271	0.000062	-0.356442	0.000092	-0.342972
N1	DT_3	-0.260523	0.031232	-0.273228	0.059164	-0.286308	0.00339	-0.270562	0.000005	-0.260384	-0.000001	-0.260436	0	-0.274457
C6	DT_3	0.191822	0.049861	0.154369	0.179309	0.08067	0.001363	0.187895	0.000038	0.198198	0.000029	0.194588	0.000005	0.19217
C5	DT_3	-0.03184	0.007327	-0.038059	0.028861	-0.061948	0.001828	-0.038318	0.000005	-0.030161	-0.000007	-0.031464	-0.000003	-0.035108
CM5	DT_3	0.057233	0.001457	0.03365	0.005351	0.002955	0.000527	0.046355	0.000004	0.064783	0	0.061346	0.000001	0.064605
P3	DC_4	0.538071	0.002053	0.533482	0.000154	0.53562	0.000163	0.533491	0	0.539272	-0.000009	0.538552	-0.000008	0.536648
PO2	DC_4	-0.412263	0.001247	-0.422644	0.000017	-0.416699	0.00006	-0.415782	0	-0.408377	0.000007	-0.412538	0.000005	-0.411919
PO1	DC_4	0.009736	0.003164	0.005824	0.000265	0.006755	0.000136	0.003986	0	0.01142	0.000002	0.010132	0.000002	0.009022
O5'	DC_4	-0.236905	0.001539	-0.232908	0.000241	-0.237999	0.00027	-0.233949	0	-0.237396	0	-0.236727	0.000001	-0.236329
O3'	DC_4	-0.237631	0.000554	-0.241308	0.000215	-0.239195	-0.000013	-0.240512	0.000001	-0.238967	0.000001	-0.237394	0.000001	-0.237856
C1'	DC_4	0.256948	0.008431	0.243276	0.004132	0.247539	0.014273	0.236405	0.000016	0.252551	-0.000033	0.25692	0.000013	0.259596
C2'	DC_4	0.045446	0.009998	0.033642	0.005436	0.036191	0.015357	0.02698	0.000011	0.046876	-0.000001	0.04648	0.000002	0.053399
C3'	DC_4	0.136002	0.003279	0.128328	0.001131	0.130894	0.001691	0.129508	0.000002	0.13885	-0.000001	0.136538	-0.000001	0.141566
C4'	DC_4	0.146968	0.000532	0.137028	0.000098	0.140433	0.00057	0.132216	0.000005	0.145037	0.000003	0.147503	0.000011	0.147253
C5'	DC_4	0.202064	0.00087	0.190314	0.000175	0.197224	0.000941	0.19551	0	0.203759	0	0.202458	0.000001	0.203832
O4'	DC_4	-0.230194	0.002278	-0.227353	0.000794	-0.229166	0.009886	-0.245582	0.000004	-0.230371	0.000001	-0.230358	0.000003	-0.23889
N4	DC_4	0.076337	0.059081	-0.006944	0.026552	0.022401	0.069601	-0.064235	0.000117	0.083519	0.000022	0.079647	0	0.083003
C4	DC_4	0.272265	0.110536	0.219556	0.055086	0.245724	0.198572	0.162421	0.000028	0.273358	0.000012	0.272261	-0.000002	0.271034
N3	DC_4	-0.35452	0.050607	-0.384776	0.02595	-0.370161	0.088593	-0.402995	-0.000019	-0.358191	0.000011	-0.35583	0.000003	-0.364954
C2	DC_4	0.350021	0.010551	0.338898	0.005678	0.343225	0.010373	0.331591	0.000076	0.348293	0.00003	0.34896	-0.000016	0.35397

O2	DC$_4$	-0.355517	-0.001587	-0.389177	-0.001171	-0.371802	-0.004428	-0.402697	0.000349	-0.357687	0.000026	-0.35602	-0.000002	-0.354273
N1	DC$_4$	-0.250627	0.059944	-0.272905	0.04061	-0.266018	0.100222	-0.291769	0.00006	-0.251808	-0.000007	-0.250622	-0.000003	-0.263025
C6	DC$_4$	0.20732	0.213313	0.081743	0.135223	0.129807	0.393757	0.04889	0.000008	0.212098	-0.000001	0.208728	-0.000002	0.215863
C5	DC$_4$	0.012444	0.045101	-0.044253	0.025876	-0.031779	0.031438	-0.069233	0.000064	0.020072	0.000002	0.014786	0.000001	0.014252
P4	DT$_5$	0.538597	0.000862	0.534196	0.000029	0.536813	0.000088	0.538319	0	0.539648	-0.00001	0.539033	-0.000009	0.542685
PO2	DT$_5$	-0.41201	0.000401	-0.421189	0.000048	-0.415207	0.000003	-0.410148	0	-0.408524	0.000009	-0.412467	0.000007	-0.404463
PO1	DT$_5$	-0.008357	0.002538	-0.012194	0.000161	-0.010151	0.000205	-0.013411	0	-0.005633	0.000001	-0.007914	0	-0.007898
O5'	DT$_5$	-0.237606	0.000317	-0.23638	0.000037	-0.238184	0.000014	-0.237354	0	-0.237484	-0.000001	-0.237384	0.000001	-0.2346
O3'	DT$_5$	-0.105791	0.000265	-0.113253	0.00002	-0.10794	0	-0.102084	0.000001	-0.103106	0.000002	-0.105516	0	-0.103332
C1'	DT$_5$	0.255852	0.001792	0.249914	0.000858	0.251906	0.000009	0.25917	0.000032	0.25453	0	0.256001	-0.000001	0.26006
C2'	DT$_5$	0.06738	0.003757	0.058344	0.001791	0.063359	0.000018	0.071012	0.000016	0.071321	0.000002	0.068136	0.000001	0.069671
C3'	DT$_5$	0.118874	0.000826	0.115077	0.00019	0.118057	0.000002	0.11369	0.000004	0.121666	-0.000002	0.119119	0	0.115641
C4'	DT$_5$	0.131811	0.000197	0.127087	0.000039	0.130296	0	0.127498	0.000001	0.130816	-0.000001	0.131984	0	0.128652
C5'	DT$_5$	0.203459	0.000358	0.198108	0.00007	0.202469	0.000009	0.198117	0.000001	0.202374	0	0.20333	0	0.200772
O4'	DT$_5$	-0.252225	0.000888	-0.249557	0.000561	-0.252952	0.000007	-0.261267	0.000012	-0.255241	0.000001	-0.25241	0	-0.258361
O4	DT$_5$	-0.367055	0.019799	-0.376581	0.012808	-0.37638	0.002044	-0.358363	-0.000003	-0.362989	0.000028	-0.36673	0.000017	-0.348862
C4	DT$_5$	0.256222	0.027214	0.243716	0.019023	0.247255	0.001989	0.257991	0.000024	0.255479	-0.000019	0.255716	-0.000009	0.259946
N3	DT$_5$	-0.097976	0.011312	-0.109743	0.006881	-0.105386	0.001153	-0.106621	0.000184	-0.101292	0.000003	-0.098378	-0.000005	-0.105167
C2	DT$_5$	0.359433	0.002169	0.353963	0.000702	0.354028	0.000845	0.364333	0.000267	0.358068	-0.000002	0.359111	-0.000007	0.363518
O2	DT$_5$	-0.388529	-0.000275	-0.402513	-0.000614	-0.39386	0.000737	-0.378689	0.000175	-0.385675	0.00003	-0.388	0.000013	-0.377418
N1	DT$_5$	-0.259624	0.008328	-0.267653	0.005522	-0.262366	-0.000031	-0.274895	0.000291	-0.260895	0.000009	-0.259258	-0.000001	-0.271385
C6	DT$_5$	0.191474	0.043459	0.158747	0.033878	0.173738	0.001308	0.178431	-0.000001	0.19364	-0.000003	0.192264	0	0.189837
C5	DT$_5$	-0.036362	0.0118	-0.037509	0.001997	-0.038814	0.000598	-0.034523	0.000152	-0.035815	0.000005	-0.036576	-0.000001	-0.037714
CM5	DT$_5$	0.043302	0.012923	0.024146	0.003739	0.026811	0.015098	0.033622	0.000033	0.048668	0	0.04448	0	0.045811
O5'	DA$_1$	-0.04865	0.000002	-0.048719	0	-0.049534	0.000001	-0.050008	0	-0.048505	0	-0.04859	0	-0.049397
O3'	DA$_1$	-0.238282	0	-0.239387	0	-0.237939	0	-0.239013	0	-0.236696	0	-0.237956	0.000003	-0.238441

C1'	DA_1	0.240957	0.000032	0.2402	0.000004	0.239507	0.000002	0.241721	0.000027	0.240956	-0.000002	0.240964	-0.000001	0.241008
C2'	DA_1	0.042243	0.000017	0.04275	0.000004	0.043114	0.000002	0.04336	0.000051	0.039665	0.000001	0.04213	0.000002	0.043851
C3'	DA_1	0.13987	-0.000001	0.137892	0	0.140026	0	0.139765	-0.000002	0.14199	-0.000001	0.139975	-0.000001	0.140857
C4'	DA_1	0.1395	0.000001	0.136876	0	0.139125	0	0.13809	0.000002	0.142578	0	0.139761	0	0.139
C5'	DA_1	0.174538	0.000004	0.171602	0.000002	0.175067	0.000002	0.1738	0.00001	0.178375	0	0.174804	0	0.174329
O4'	DA_1	-0.274538	0.000008	-0.275401	0.000002	-0.274373	0	-0.275145	0.000018	-0.272433	0.000002	-0.274172	0.000004	-0.274007
N6	DA_1	0.051265	0.002155	0.052078	0.000585	0.04707	0.004724	0.001175	0.000345	0.055042	-0.000008	0.052003	0.000001	0.028469
C6	DA_1	0.256257	0.001105	0.257105	0.000573	0.255761	0.000469	0.25184	0.000362	0.256012	0	0.256296	0.000003	0.253106
C5	DA_1	0.054207	0.000191	0.053944	-0.00002	0.05506	0.000046	0.060041	0.002325	0.055345	0.000051	0.05454	0.000007	0.06025
N7	DA_1	-0.402409	0.000265	-0.402012	0.00011	-0.402293	0.000002	-0.40776	0.000612	-0.399217	0.000004	-0.401901	-0.000026	-0.404693
C8	DA_1	0.276638	0.00006	0.269069	0.000028	0.274431	0.000077	0.277743	0.002431	0.282646	0.000053	0.27707	-0.000003	0.28112
N9	DA_1	-0.257141	0.000176	-0.26026	0.000013	-0.258355	-0.00001	-0.264653	0.000307	-0.256894	0.000009	-0.257111	0.00003	-0.264168
C4	DA_1	0.214887	0.000586	0.212079	0.00023	0.213764	0.000059	0.218969	0.001085	0.214924	0.000019	0.21482	-0.000003	0.219119
N3	DA_1	-0.37906	0.000044	-0.385563	-0.000043	-0.382238	0.00016	-0.381877	0.00131	-0.376668	0.000027	-0.379347	-0.000011	-0.377523
C2	DA_1	0.288282	0.000757	0.286586	0.000291	0.286928	-0.000039	0.287304	0.00036	0.2889	0.000007	0.288367	0.000001	0.28851
N1	DA_1	-0.356812	0.000653	-0.353571	0.000088	-0.354386	0.000106	-0.356201	0.000397	-0.357481	0.000006	-0.356372	0.000003	-0.360303
P5	DG_2	0.537015	0.000001	0.535634	0.000003	0.536524	0.000003	0.535352	0	0.53818	-0.00001	0.537345	-0.000024	0.535318
PO2	DG_2	-0.413257	0	-0.415567	-0.000003	-0.414345	-0.000002	-0.412107	0	-0.409177	0.000005	-0.413765	0.000028	-0.412579
PO1	DG_2	-0.008275	0.000001	-0.008616	0	-0.008261	0	-0.009992	0	-0.008271	0.000002	-0.008143	-0.000001	-0.008329
O5'	DG_2	-0.240604	0.000001	-0.240202	0	-0.240841	0	-0.24022	0	-0.242512	0.000002	-0.240498	-0.000004	-0.243048
O3'	DG_2	-0.23421	0.000002	-0.235541	0.000001	-0.233774	0.000001	-0.240082	0.000005	-0.230433	0.000003	-0.232954	0.000002	-0.238118
C1'	DG_2	0.256067	0.000109	0.255674	0.00002	0.254887	0.000001	0.261304	-0.000032	0.256661	-0.000013	0.256327	0.000022	0.259773
C2'	DG_2	0.06648	0.000055	0.065333	0.000006	0.066664	0.000008	0.049686	0.000166	0.064641	0.000022	0.067221	0.000154	0.051062
C3'	DG_2	0.152802	0.000005	0.149451	0.000001	0.152529	0.000001	0.138676	0.000005	0.157107	0.000002	0.153536	0.000008	0.14609
C4'	DG_2	0.149619	0.000012	0.148245	0.000002	0.149432	0.000001	0.145494	-0.000011	0.153111	0	0.150462	0.000001	0.149265
C5'	DG_2	0.210826	0.000003	0.207879	0.000001	0.211261	0.000001	0.209688	0.000003	0.215767	0	0.211366	0	0.210126
O4'	DG_2	-0.234086	0.000017	-0.234575	0.000005	-0.235056	0.000005	-0.235119	0.000031	-0.231857	0.00001	-0.233726	0.000001	-0.228265

N2	DG_2	0.026092	0.002521	0.022884	0.001145	0.021821	0.001187	0.022396	0.035138	0.050642	0.002405	0.027627	0.000146	0.021325
O6	DG_2	-0.387772	0.001769	-0.384992	0.000416	-0.39156	0.000294	-0.374394	0.021813	-0.367094	0.00157	-0.385882	0.000041	-0.364297
C6	DG_2	0.244561	0.005167	0.242601	0.001002	0.243387	0.000876	0.241794	0.004705	0.249994	0.000153	0.245071	0.000011	0.246807
C5	DG_2	0.032321	0.000797	0.03085	0.000106	0.032712	-0.000056	0.036506	0.035379	0.049545	0.001215	0.034145	0.000018	0.040074
N7	DG_2	-0.391428	0.001007	-0.392905	0.000105	-0.39241	0.000043	-0.395098	0.008226	-0.37874	0.000355	-0.389382	-0.000013	-0.389195
C8	DG_2	0.288281	0.000152	0.274068	-0.000074	0.282658	-0.000006	0.281845	0.022618	0.312487	0.000454	0.291361	0.000037	0.291126
N9	DG_2	-0.250431	0.000444	-0.255117	0.000122	-0.252408	0.000012	-0.260911	0.002002	-0.246715	0.00016	-0.24981	0.000013	-0.257324
C4	DG_2	0.222571	0.002654	0.217956	0.000484	0.221159	0.000242	0.21624	0.008528	0.227711	-0.000169	0.222966	0.000074	0.22064
N3	DG_2	-0.37689	-0.000181	-0.384823	-0.000167	-0.380242	-0.000201	-0.407446	0.045799	-0.355534	0.003495	-0.375746	0.000099	-0.390234
C2	DG_2	0.372141	0.00275	0.37211	0.000975	0.371944	0.000637	0.365255	0.004866	0.3754	-0.000046	0.370848	0.000105	0.36976
N1	DG_2	-0.088221	0.00916	-0.088531	0.003355	-0.086556	0.005402	-0.102852	-0.002119	-0.087261	-0.000059	-0.088916	0.000081	-0.094601
P6	rA_3	0.546458	0.000009	0.544712	0	0.545196	0.000001	0.538896	0.000017	0.547835	-0.00002	0.547858	-0.000007	0.54179
PO2	rA_3	-0.379672	0.000001	-0.381981	0	-0.380052	-0.000002	-0.405812	-0.000003	-0.373527	0.00003	-0.378222	0.000008	-0.399814
PO1	rA_3	0.026186	0.000005	0.024126	0	0.025526	0.000001	0.013086	0.0001	0.026379	0.000125	0.028025	0.00002	0.019967
O5'	rA_3	-0.231161	0.000011	-0.230511	0	-0.232716	0.000001	-0.229763	-0.000009	-0.236132	-0.000015	-0.232706	0.000013	-0.23156
O3'	rA_3	-0.234859	0.000001	-0.234577	0.000001	-0.234795	0	-0.231351	0.000013	-0.232972	0.000018	-0.232931	0.000007	-0.232253
C1'	rA_3	0.251207	0.000072	0.250409	0.000023	0.248702	0.000001	0.247603	0.00009	0.25106	0.000044	0.251578	0.000091	0.254983
C2'	rA_3	0.104858	0.000038	0.103629	0.000008	0.104925	0.000004	0.120475	0.00145	0.103925	0.001483	0.105647	0.000618	0.130578
O2'	rA_3	-0.125922	0.000005	-0.128389	0.000001	-0.126282	0.000002	-0.114341	0.001575	-0.118806	0.001835	-0.116706	0.000621	-0.087278
C3'	rA_3	0.137057	0.000008	0.134497	0.000004	0.137382	0	0.162965	0.000058	0.142337	0.000053	0.140886	0.000017	0.163217
C4'	rA_3	0.160247	0.000039	0.158986	0.000006	0.159472	0	0.158023	0.00001	0.168422	0.000023	0.16528	0.000004	0.166595
C5'	rA_3	0.195839	0.000035	0.195695	0.000007	0.196249	0	0.211216	-0.000039	0.201226	0.000033	0.198849	0.00002	0.210037
O4'	rA_3	-0.253596	0.000045	-0.251321	0.000023	-0.253545	-0.000002	-0.260302	0.000241	-0.249676	0.000042	-0.249161	0.000025	-0.238368
N6	rA_3	0.057159	0.004894	0.054178	0.004166	0.04383	0.000395	0.043009	0.027435	0.090662	0.012135	0.081687	0.001361	0.063117
C6	rA_3	0.2627	0.001688	0.26587	0.002603	0.26322	0.000468	0.25836	0.006541	0.267595	0.002314	0.265131	0.000279	0.260161
C5	rA_3	0.058041	0.000916	0.058185	0.000231	0.059341	-0.000012	0.065033	0.041128	0.07218	0.027624	0.066359	0.008343	0.064874
N7	rA_3	-0.3768	0.000003	-0.375989	0.000162	-0.375462	0.000013	-0.369709	0.009416	-0.363315	0.001626	-0.364192	-0.00073	-0.367643

C8	rA_3	0.287154	0.000923	0.274934	-0.000138	0.278937	0.000137	0.303948	0.028998	0.309043	0.019574	0.3035	0.007611	0.307107
N9	rA_3	-0.260237	0.0004	-0.265922	0.000159	-0.265237	-0.000032	-0.262119	0.001301	-0.262507	-0.000059	-0.263324	0.000038	-0.264655
C4	rA_3	0.218296	0.000296	0.214536	0.000746	0.21428	0.000106	0.218647	0.01824	0.218561	0.009413	0.214463	0.003569	0.21747
N3	rA_3	-0.377409	0.00057	-0.386934	-0.000117	-0.387606	-0.000069	-0.384999	0.005787	-0.375952	0.001971	-0.377129	0.001571	-0.375519
C2	rA_3	0.298607	0.003151	0.301591	0.001174	0.296511	0.000907	0.291896	0.006164	0.307847	0.000452	0.303918	0.00089	0.300489
N1	rA_3	-0.346757	0.000542	-0.337094	0.000401	-0.339169	0.000443	-0.349939	-0.001309	-0.341814	0.000701	-0.343929	0.002112	-0.350391
P7	$^{O}G_4$	0.518994	0.000002	0.517745	-0.000002	0.517708	0.000002	0.518955	0.000023	0.520916	0	0.520832	-0.000011	0.52303
PO2	$^{O}G_4$	-0.049381	0.000002	-0.051456	0	-0.048979	0.000001	-0.017703	0.000002	-0.04238	0.000003	-0.046524	0.000001	-0.007207
PO1	$^{O}G_4$	-0.465513	0	-0.468095	0.000003	-0.467091	-0.000003	-0.451865	0.000048	-0.460717	0.000073	-0.462826	0.00001	-0.446665
O5'	$^{O}G_4$	-0.244707	0	-0.245796	0	-0.244519	0	-0.241867	0.000008	-0.244816	0.000009	-0.243908	-0.000002	-0.24131
O3'	$^{O}G_4$	-0.24079	0.000003	-0.240676	0	-0.240521	0	-0.239675	0.000072	-0.238311	0.000068	-0.238282	0.000013	-0.24037
C1'	$^{O}G_4$	0.251786	0.00004	0.252197	0.000004	0.250796	0	0.254222	0.000245	0.260592	-0.000465	0.266213	-0.000785	0.25962
C2'	$^{O}G_4$	0.040442	0.000005	0.037881	0	0.040866	-0.000001	0.034971	0.000781	0.045556	0.000535	0.050332	-0.000183	0.04351
C3'	$^{O}G_4$	0.136613	0.000003	0.134819	0	0.136844	0	0.135751	0.000234	0.142978	0.00017	0.143116	-0.000039	0.140564
C4'	$^{O}G_4$	0.142705	0.000034	0.143244	0.000002	0.14281	0	0.140701	0.000033	0.149887	-0.000008	0.151092	-0.000063	0.145497
C5'	$^{O}G_4$	0.219039	0.000009	0.219584	0.000002	0.219156	0.000001	0.21603	0.00007	0.226578	0.00005	0.22568	0.000004	0.221551
O4'	$^{O}G_4$	-0.256604	0.000013	-0.254392	0.000002	-0.257125	0	-0.247278	0.000635	-0.256999	0.00042	-0.253004	-0.000154	-0.251495
N2	$^{O}G_4$	0.139981	0.000028	0.133466	0.000003	0.138386	0.000004	0.089318	0.045417	0.208002	0.070451	0.235645	0.071189	0.181035
O6	$^{O}G_4$	-0.360807	0.000159	-0.357487	0.000058	-0.358927	-0.000001	-0.356102	0.05521	-0.305319	0.090509	-0.285048	0.081195	-0.298018
C6	$^{O}G_4$	0.245651	0.000079	0.246318	0.000023	0.245067	0.000003	0.24801	0.026727	0.263605	0.034902	0.278423	0.054178	0.270225
C5	$^{O}G_4$	0.056813	0.000023	0.058988	0.000019	0.058102	0.000005	0.05893	0.142091	0.109172	0.207786	0.135394	0.178271	0.107988
N7	$^{O}G_4$	-0.101129	0.000125	-0.098623	0.00013	-0.101115	0.000025	-0.104956	0.103023	-0.051701	0.148602	-0.027922	0.191995	-0.326517
C8	$^{O}G_4$	0.34104	0.000094	0.340898	0.000028	0.339599	0.000006	0.343688	0.020897	0.362821	0.019233	0.374488	0.047032	0.347426
O8	$^{O}G_4$	-0.40471	0.000161	-0.402665	0.000036	-0.404224	0.000001	-0.393784	0.098356	-0.318821	0.149714	-0.288546	0.150881	-0.321626
N9	$^{O}G_4$	-0.255677	0.000018	-0.257838	-0.000003	-0.257021	-0.000001	-0.26462	0.012672	-0.242796	0.008907	-0.237072	0.0036	-0.258134
C4	$^{O}G_4$	0.234703	0.000009	0.232285	-0.000002	0.232247	0.000001	0.231345	0.057662	0.264499	0.072106	0.280324	0.095761	0.269527
N3	$^{O}G_4$	-0.354971	0	-0.357409	0.000001	-0.356534	0.000002	-0.361845	0.036289	-0.329187	0.053208	-0.317782	0.043001	-0.345182

C2	$^{O}G_4$	0.385162	0.000015	0.38371	0	0.383776	0.000002	0.380335	0.019367	0.405621	0.021822	0.417237	0.041046	0.403144
N1	$^{O}G_4$	-0.013522	0.000031	-0.015777	-0.000004	-0.014133	0	-0.020551	-0.002795	0.007736	-0.008821	0.023166	-0.008417	-0.007645
P8	DA_5	0.534786	0.000007	0.533465	0.000001	0.534286	0.000003	0.53373	0.000006	0.537758	-0.000023	0.537047	-0.000019	0.535818
PO2	DA_5	-0.415736	0.000003	-0.41762	0	-0.416832	-0.000003	-0.414482	0.000004	-0.40669	0.000019	-0.41333	0.000009	-0.410962
PO1	DA_5	-0.011445	0.000007	-0.012867	0	-0.011641	-0.000001	-0.014452	0.000004	-0.009445	0.000007	-0.009028	0.000002	-0.009667
O5'	DA_5	-0.239987	0.000002	-0.240704	0	-0.24038	0.000001	-0.24211	0.000003	-0.240755	0.000002	-0.239531	0	-0.240214
O3'	DA_5	-0.102928	0.000004	-0.104753	0.000001	-0.103545	0	-0.102674	0.000036	-0.096081	0.000039	-0.099896	0.000019	-0.100948
C1'	DA_5	0.258798	0.000021	0.259497	0.000002	0.258069	0.000001	0.262216	0.000509	0.261061	0.000494	0.263743	0.000392	0.263968
C2'	DA_5	0.063571	0.000008	0.060989	0.000001	0.063823	0	0.06432	0.000348	0.071251	0.00043	0.070439	0.00025	0.068752
C3'	DA_5	0.119368	0.000003	0.1175	0	0.119685	0	0.120673	0.000113	0.124003	0.000117	0.122293	0.000058	0.12141
C4'	DA_5	0.137279	0.000042	0.137906	0.000004	0.137573	0.000001	0.139321	0.000084	0.140537	0.00008	0.141665	0.000062	0.140032
C5'	DA_5	0.20163	0.000029	0.20157	0.000001	0.202304	0	0.202846	0.000037	0.205873	0.000032	0.205058	0.000018	0.204718
O4'	DA_5	-0.241656	0.000003	-0.240491	0	-0.242537	0	-0.241426	0.000665	-0.24852	0.000833	-0.245519	0.000766	-0.244385
C6	DA_5	0.260431	0.000061	0.26094	0.00003	0.260478	0	0.256857	0.002133	0.261467	0.002583	0.262168	0.001405	0.257308
C5	DA_5	0.062261	0.000009	0.062587	0	0.063469	0.000001	0.069245	0.002154	0.058163	0.001532	0.059542	0.00034	0.06713
N7	DA_5	-0.376306	0.000008	-0.376017	0.000008	-0.375975	0.000002	-0.376555	0.006607	-0.378929	0.008204	-0.373183	0.004182	-0.374822
C8	DA_5	0.311624	0.000054	0.307784	0.000003	0.308839	0	0.313445	0.007093	0.313663	0.008225	0.318515	0.004192	0.32259
N9	DA_5	-0.245773	0.000003	-0.247141	-0.000003	-0.24664	-0.000001	-0.253498	0.004002	-0.245611	0.005158	-0.244611	0.003631	-0.250553
C4	DA_5	0.219562	0.000007	0.219289	0.000008	0.21913	0.000001	0.224997	0.004213	0.220172	0.004853	0.221536	0.002973	0.225052
N3	DA_5	-0.382705	0.000033	-0.383895	0.000016	-0.385076	0.000001	-0.380628	0.001991	-0.376242	0.001609	-0.375919	-0.00012	-0.376657
C2	DA_5	0.293468	0.000102	0.293602	0.000082	0.293248	0.000001	0.293671	0.003317	0.309199	0.004106	0.305593	0.001859	0.297619
N1	DA_5	-0.350364	0.000003	-0.349437	0.00001	-0.347934	0.000001	-0.352692	0.000231	-0.344089	0.000025	-0.346092	-0.000007	-0.355127
N6	DA_5	0.042463	0.000048	0.040128	0.000035	0.0401	0	0.002609	0.002502	0.050549	0.002894	0.05586	0.001705	0.032323

Table S6. Local riboadenosine sugar-pucker and conformational descriptors for the neutral, adiabatic cation, and adiabatic anion structures. ν0–ν4 are the five endocyclic furanose torsion angles; glicosidic torsion angle χ = C4–N9–C1′–O4′; dihedral angle indicated 2′-OH position denoted as ε* = N9–C1′–C2′–O2′; pseudorotation phase and amplitude are reported in degrees [^{O}].

Form	Dihedral angels							Phase	Amplitude
	υ0 C4′-O4′-C1′-C2′	υ1 O4′- C1′- C2′- C3′	υ2 C1′-C2′-C3′-C4′	υ3 C2′-C3′-C4′-O4′	υ4 C3′- C4′- O4′- C1′	χ C4-N9-C1′-O4′	ε* N9-C1′-C2′-O2′		
**^{O}GrAG-DNA**									
Neutral	-18.71	34.57	-36.89	27.48	-5.52	-90.22	-87.93	169.87	37.47
Cation	-21.1	38.56	-40.83	29.9	-5.53	-85.09	-84.85	169.09	41.58
Anion	-21.72	38.91	-40.81	29.54	-4.3	-92.07	-82.97	161.11	43.13
**^{O}GrAG-DNA**									
Neutral	-37.54	47.25	-37.77	18.45	11.9	-99.09	-72.49	146.06	45.53
Cation	-40.84	50.35	-40.35	18.61	13.58	-97.09	-72.73	145.25	49.11
Anion	-42.17	49.64	-38.56	15.89	16.13	-79.36	-115.91	142.20	48.80
**rA^{O}G-DNA**									
Neutral	-40.73	49.01	-38.43	15.93	15.51	-105.28	-66.96	142.94	48.16
Cation	-33.12	46.03	-40.96	23.03	6.35	-103.02	-73	153.64	45.71
Anion	-40.23	49.49	-39.76	17.59	14.21	-102.76	-69	144.80	48.66
**^{O}GA-DNA**									
Neutral	5.68	16.7	-31.84	35.39	-26.05	-98.44	-106.2	207.23	35.81
Cation	-11.8	31.67	-38.52	32.63	-13.3	-90.6	-84.56	181.19	38.53
Anion	-29.15	42.39	-38.34	28.19	3.69	-98.58	-76.03	158.27	41.27

Figure S1. Graphical representation of investigated d[AGrAGA]*d[TCTCT] duplex (example) was divided into two levels of calculation using the ONIOM (Our own N-layered Integrated Molecular Orbital and Molecular Mechanics) method, i.e., High Level, M06-2X/D95**-HL (all the Base Pair Ladder), and low level, M06-2X/sto-3G-LL (sugar–phosphate backbones) in the aqueous phase.

The graphical represantation of Our own N-layered Integrated Molecular Orbital and Molecular Mechanics (ONIOM) method.

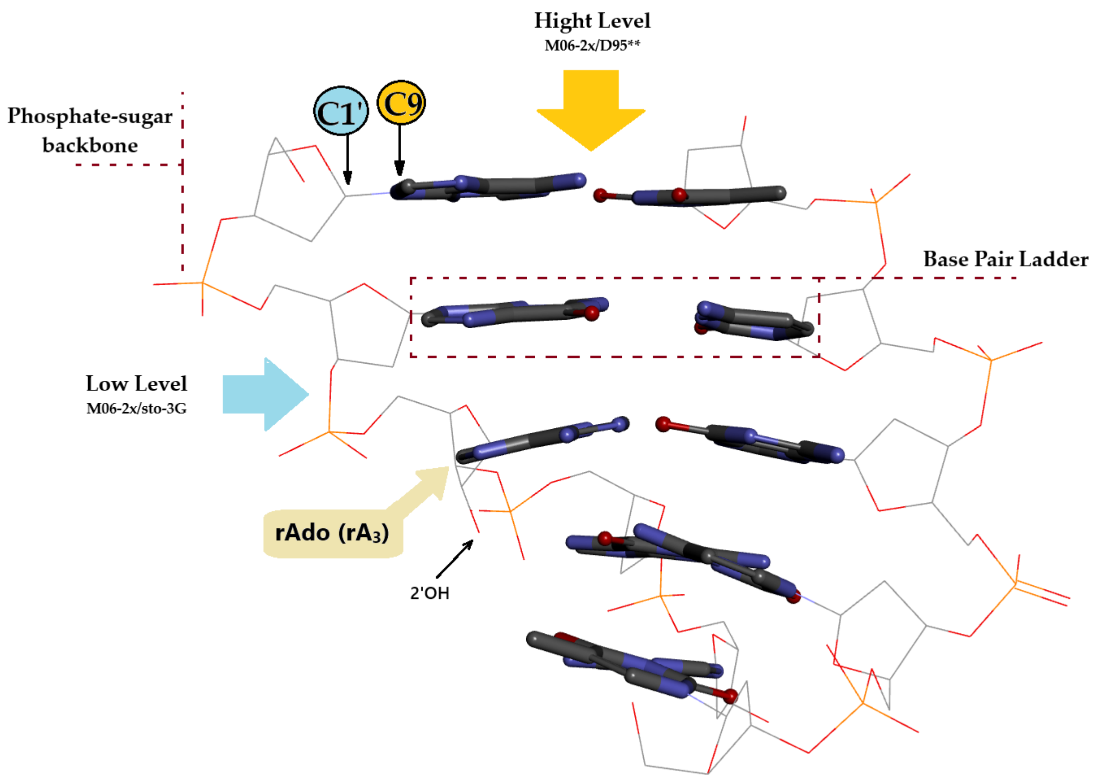

Supplement: Supplementary file 1 [file molecules-31-03673-s001.zip › Suplementary materials.pdf]
